# The *Xenopus* alcohol dehydrogenase gene family: characterization and comparative analysis incorporating amphibian and reptilian genomes

**DOI:** 10.1186/1471-2164-15-216

**Published:** 2014-03-20

**Authors:** Emma Borràs, Ricard Albalat, Gregg Duester, Xavier Parés, Jaume Farrés

**Affiliations:** 1 Department of Biochemistry and Molecular Biology, Universitat Autònoma de Barcelona, E-08193, Bellaterra, Barcelona, Spain; 2 Departament de Gènetica, Facultat de Biologia and Institut de Recerca de la Biodiversitat (IRBio), Universitat de Barcelona, Av. Diagonal, E-08028, Barcelona, Spain; 3 Development, Aging and Regeneration Program, Sanford-Burnham Medical Research Institute, 10901 North Torrey Pines Road, La Jolla, CA 92037, USA

**Keywords:** Alcohol dehydrogenase, Enzymogenesis, Gene family, Vertebrate evolution

## Abstract

**Background:**

The alcohol dehydrogenase (ADH) gene family uniquely illustrates the concept of enzymogenesis. In vertebrates, tandem duplications gave rise to a multiplicity of forms that have been classified in eight enzyme classes, according to primary structure and function. Some of these classes appear to be exclusive of particular organisms, such as the frog ADH8, a unique NADP^+^-dependent ADH enzyme. This work describes the ADH system of *Xenopus*, as a model organism, and explores the first amphibian and reptilian genomes released in order to contribute towards a better knowledge of the vertebrate *ADH* gene family.

**Results:**

*Xenopus* cDNA and genomic sequences along with expressed sequence tags (ESTs) were used in phylogenetic analyses and structure-function correlations of amphibian ADHs. Novel ADH sequences identified in the genomes of *Anolis carolinensis* (anole lizard) and *Pelodiscus sinensis* (turtle) were also included in these studies. Tissue and stage-specific libraries provided expression data, which has been supported by mRNA detection in *Xenopus laevis* tissues and regulatory elements in promoter regions. Exon-intron boundaries, position and orientation of *ADH* genes were deduced from the amphibian and reptilian genome assemblies, thus revealing syntenic regions and gene rearrangements with respect to the human genome. Our results reveal the high complexity of the ADH system in amphibians, with eleven genes, coding for seven enzyme classes in *Xenopus tropicalis*. Frogs possess the amphibian-specific ADH8 and the novel ADH1-derived forms ADH9 and ADH10. In addition, they exhibit ADH1, ADH2, ADH3 and ADH7, also present in reptiles and birds. Class-specific signatures have been assigned to ADH7, and ancestral ADH2 is predicted to be a mixed-class as the ostrich enzyme, structurally close to mammalian ADH2 but with class-I kinetic properties. Remarkably, many ADH1 and ADH7 forms are observed in the lizard, probably due to lineage-specific duplications. ADH4 is not present in amphibians and reptiles.

**Conclusions:**

The study of the ancient forms of ADH2 and ADH7 sheds new light on the evolution of the vertebrate ADH system, whereas the special features showed by the novel forms point to the acquisition of new functions following the *ADH* gene family expansion which occurred in amphibians.

## Background

Vertebrate alcohol dehydrogenases (ADH, EC1.1.1.1) are dimeric zinc-containing enzymes with a 40-kDa subunit and 373–383 amino acid residues. Structurally, they belong to the medium-chain dehydrogenase/reductase (MDR) superfamily [1]. ADHs catalyze the reversible oxidation of a wide range of alcohol substrates to the corresponding aldehydes or ketones, and can be grouped in eight enzyme classes (ADH1-8, class I to VIII) [2], according to their primary structure and function. The human *ADH* gene nomenclature used throughout the text is the enzyme class-based nomenclature currently used for vertebrate ADH [2] and differs from that approved by the Human Genome Organization (HUGO) Gene Nomenclature Committee [3], as the former facilitates comparisons with ADHs from other mammals and lower vertebrate species.

Tandem gene duplications gave rise to the multiplicity of forms in the ADH family, including isoenzymes and allelic forms in particular lineages. ADH3 is the most ancient form and the only class present before chordates. It is a glutathione-dependent formaldehyde dehydrogenase (FDH), a highly conserved and ubiquitous detoxifying enzyme. Duplication of the ancestral *ADH3* gene near the agnathan/gnathostome split originated ADH1, which evolved independently in the fish and tetrapod lines becoming the classical hepatic ethanol dehydrogenase [4,5]. In tetrapods, a second duplication of the gene coding for ADH3 generated ADH2, also hepatic but active at higher ethanol concentrations [6]. Close to the origin of mammals, ADH1 duplicated giving rise to ADH4, a highly retinoid-active enzyme [7,8] present in eye, skin and gastric tissues [9-11]. The most evolutionarily recent classes in mammals are ADH5 and ADH6 [12], the latter being absent in primates [13]. These two classes, identified at DNA level, are the most divergent within mammalian ADHs. On the other hand, ADH7, previously named ADH-F due to its fetal expression, is a steroid/retinoid dehydrogenase that was first described in chicken [14]. Finally, ADH8 is a unique NADP^+^-dependent ADH isolated from the stomach of the frog *Rana perezi* and its proposed function is the reduction of retinaldehyde to retinol [15].

Studies on amphibian ADH genetics have been scarce. Isozyme patterns of *X. laevis* liver ethanol dehydrogenase suggested the existence of two polymorphic genes encoding ADH subunits that did not form heterodimers and were located in different linkage groups [16,17]. The enzymes ADH1, ADH3 and ADH8 from the frog *Rana perezi* were purified and characterized by our group, and the ADH1 and ADH8 proteins were also sequenced [15,18]. The cloning of the cDNA of *R. perezi* ADH8 [15] allowed to perform mutagenesis studies on coenzyme specificity [19] and to obtain the crystal structure of the enzyme [20,21]. Partial cDNAs of *X. laevis* ADH1 and an ADH4-like form were cloned and used for expression analysis in embryonic and adult tissues [22]. Later, two reviews on MDR-ADH evolution [4,23], which included genomic data, provided some partial information on the amphibian ADH system.

Here the ADH system of the development model frog *X. laevis* has been further investigated, especially the retinaldehyde-active ADH8. Tetraploidy of *X. laevis* (2n = 36) was a handicap for genetic studies, thus the present work was restricted to expression patterns and extended with additional information from expressed sequence tag (EST) collections. On the other hand, its diploid relative *X. tropicalis* (2n = 20), the subject of the only amphibian genome project, was used for a genomic approach to the amphibian ADH family. Since the reptile genome of the anole lizard (*Anolis carolinensis*) and the turtle (*Pelodiscus sinensis*) had been sequenced at the time of the study, the *ADH* gene sequences from these organisms could be identified and used in phylogenetic analyses and genomic comparisons.

The joint analysis of *Xenopus* genome-wide data and the results of the expression analysis described herein provide an integrated view of the amphibian ADH system. Moreover, since this organism occupies a key phylogenetic position, this work provides insight into the molecular evolution of the *ADH* gene family in vertebrates.

## Methods

### Animal tissues

Tissues were obtained from adult *X. laevis* females (130 mm long) provided by Horst Kähler (Hamburg, Germany). The animals were kept in an ice bath for 15 min to diminish their metabolism prior to euthanasia. After decapitation, the head was immersed in liquid nitrogen to assure total unconsciousness, as recommended [24]. The organs were then removed, cleaned, rinsed in distilled water and stored at –80°C. Prior to analysis, frozen tissues were pulverized in liquid nitrogen and homogenized. This study was approved by the Ethical Committee of the Universitat Autònoma de Barcelona.

### Isolation and cloning of *X. laevis* cDNAs

Stomach poly(A)^+^ RNA (2 μg) was isolated with the “QuickPrep Micro mRNA purification kit” (GE Healthcare) and a cDNA pool was synthesized using the “First Strand cDNA Synthesis kit for RT-PCR (AMV)” (Roche) with the oligo (dT)_17_R_I_R_O_ primer adaptor [25]. Nested-PCRs combined degenerate primers based on *R. perezi* ADH8 (Table 1) and amplification products were cloned into pBluescript II SK(+) (Stratagene) and sequenced. The partial cloned sequences were later identified as *ADH1B* and *ADH3*, whereas the *ADH8B* and *ADH9* partial cDNAs were obtained as described [21]. The 3′-ends were amplified by rapid amplification of cDNA ends [25] combining the adaptor-specific primers R_O_ and R_I_ with specific forward primers (Table 1), and then cloned and sequenced as described above.

**Table 1:** **Oligonucleotide primers for amplification of *X. laevis *** **ADHs**

**Name**	**Nucleotide sequence**^**1**^	**Amino acid residues**^**2**^
Degenerate primers (based on *Rana perezi ADH8*)		
Degenerate forward outer	5′-ATGTGCACTGCGGGIAARGWIATHA-3′	1-8
Degenerate reverse outer	5′-TAGTCTTTTGGRTTIADRCAYTC-3′	237-244
Degenerate forward inner	5′-ATTACATGTAAGGCIGCIGTIGC-3′	7-14
Degenerate reverse inner	5′-GGCTTTTGGRAAYTTRTCYTTRT-3′	223-230
Specific forward primers (used in combination with 3′-end adaptors)		
*ADH1B* outer	5′-GTATAGTGGAAAGTGTGGGAGAG-3′	72-79
*ADH1B* inner	5′-CATACATTGGACTCTTGTTGGAC-3′	119-126
*ADH3* outer	5′-CTGAATACACTGTTGTAGC-A-3′	148-154
*ADH3* inner	5′-CAACTGGTTATGGAGCTGTG-3′	178-184
*ADH8B* outer	5′-AGCACTTTTACAGAATACAG-3′	144-150
*ADH8B* inner	5′-AGATTCCTCCAGGATCTACG-3′	186-192
*ADH9* outer	5′-GCTAAAGTACAGCAAGGTAG-3′	189-194
*ADH9* inner	5′-GAATCATTGGAGTAGACATT-3′	219-225
Specific reverse primers for *ADH8B* (used in combination with the above specific forward primers)		
*ADH8B* reverse outer	5′-TCAACAGGATGTCAGGCTGCAAATG-3′	Nucleotides 61–37 of non-coding 3′-end
*ADH8B* reverse inner	5′-AATGACCGTAGTGGACTTCACACGA-3′	Nucleotides 40–16 of non-coding 3′-end

^1^Degenerate nucleotides are R (A or G), W (A or T), H (A or C or T) and I (inosine, able to base pair with any natural nucleotide). ^2^Numbering refers to amino acid residues unless otherwise indicated.

### Northern blot analysis of *X. laevis ADH1B*, *ADH3* and *ADH9*

Total RNA from stomach, liver, kidney and intestine was isolated by the acid guanidinium thiocyanate method [26]. Samples (15 μg) were electrophoresed on a 1% agarose gel containing 2.6 M formaldehyde and transferred onto a Nylon filter. 18S rRNA (1.8 kb) and 28S rRNA (4.1 kb) were used to check the integrity and amount of loaded RNA and to estimate the size of the RNA hybrids. Probes included *X. laevis ADH1B*, *ADH3* and *ADH9* cDNAs (their 3′-end moieties of ~700 bp), labeled with [α-^32^P]dCTP (GE Healthcare) by a random hexamer-primed method using the “Prime-a-Gene Labeling System” (Promega) except for the Klenow enzyme (Invitrogen). After a 45-min prehybridization at 60°C in 0.2 M sodium phosphate, pH 7.2, 1 mM EDTA, 1% BSA and 1% SDS, filters were hybridized for 18–24 h at 60°C in the presence of 10^6^ cpm/ml of radiolabeled probe. Final 30-min washes at 60°C were performed twice in 40 mM sodium phosphate, pH 7.2, 1 mM EDTA and 1% SDS. Autoradiography was carried out at –80°C for 2–5 days with Hyperfilm-MP (GE Healthcare) using an intensifying screen. Hybridization signals were then scanned in a Bio-Rad GS-700 imaging densitometer.

### RT-PCR of *X. laevis ADH8B*

cDNA pools from esophagus, stomach, intestine and liver were prepared from 3–8 μg of total RNA using the *ADH8B*-specific reverse outer primer described in Table 1. First PCR amplification combined this primer with the *ADH8B* forward outer primer, and a second round used the inner primer pair (Table 1), generating a 603-bp *ADH8B* cDNA fragment.

### Starch gel electrophoresis and activity staining of *X. laevis* ADH1 and ADH3

Tissues were homogenized (1:1, w/v) in 30 mM Tris–HCl, pH 7.6, 0.5 mM dithiothreitol, centrifuged at 27000 ×g for 30 min and supernatants were used for analysis by starch gel electrophoresis [27]. Total protein was determined by the Bio-Rad protein assay method, using bovine serum albumin as standard. In order to discriminate between the NAD^+^-dependent classes ADH1 and ADH3, gel slices were stained for ADH activity with 0.1 M 2-buten-1-ol and 0.6 mM NAD^+^ (grade AA1, Sigma), to mainly detect ADH1, and for glutathione-dependent FDH activity with 4.8 mM formaldehyde and 1 mM glutathione, to specifically stain ADH3.

### Identification of *Xenopus* ADH sequences in protein and expression databases

Several ADH protein sequences from both *Xenopus* species were gathered at UniProt [28], first by name search and then by exploring clusters with 90% or 50% identity. ESTs of *X. laevis* and *X. tropicalis* were obtained by BLAST (Basic Local Alignment Search Tool) [29] search, using *X. laevis* and other vertebrate ADHs as protein queries against translated nucleotide sequences (TBLASTN), at the Sanger Institute *X. tropicalis* EST Project [30] and TGI Gene Indices [31] websites. Additional information on the expression sites of *Xenopus* ADH clustered transcripts was obtained by name search at the NCBI UniGene data bank [32].

### Identification and analysis of *ADH* genes in *Xenopus tropicalis, Anolis carolinensis* and *Pelodiscus sinensis* genomes

*X. tropicalis* genome assembly 4.2, *A. carolinensis* AnoCar2.0 and *P. sinensis* PelSin_1.0 were interrogated with BLAST-based search tools to identify possible *ADH* gene locations. For *X. tropicalis*, TBLASTN searches were undertaken at the Joint Genome Institute *X. tropicalis* Genome Assembly 4.1 website [33] using *X. laevis* ADHs as protein queries. For *A. carolinensis* and *P. sinensis*, TBLASTN searches were conducted using the Ensembl genome browser [34] to compare *X. tropicalis* ADHs against genomic databases, allowing some local mismatch. Protein BLAT (BLAST-Like Alignment Tool) analyses [35] using the UCSC web browser [36] were also performed and the same scaffolds producing significant alignments were obtained.

These *ADH*-containing scaffolds were then exported with Ensembl, 3-frame translated in both orientations, and manually screened for the presence of *ADH* genes either using exon sequences derived from *X. tropicalis* ESTs or consensus ADH sequences. The Ensembl Genome Browser [37] was also used to align syntenic regions of the genomes studied. Exon-intron boundaries were determined according to the general GT/AG consensus, and intron lengths and discontinuities in the DNA sequence were annotated. The first 600 bp of the 5′-non-coding regions of *X. tropicalis ADH* genes were checked for potential transcription factor binding sites using MATCH [38], based on the TRANSFAC database of position weight matrices, using 100% coincidence for the core and 95% for the whole matrix. In addition, the first 650 bp of the 3′-non-coding regions were screened for polyadenylation signals.

### Sequence alignment and phylogenetic analyses

Sequence analysis and manipulation were carried out using BioEdit Sequence Alignment Editor, version 7.0.4.1 [39]. Multiple sequence alignments were performed with Clustal Omega [40,41]. Gaps and missing positions were not removed from the alignment and trees were constructed considering partial deletions for the pairwise comparisons. Phylogenetic analyses were conducted using MEGA version 5 [42]. Unrooted phylogenetic trees were constructed by Neighbour-joining (NJ) [43] using the JTT (Jones-Taylor-Thornton) matrix [44] for amino acid distance calculations. Evolutionary rates among sites were considered γ-distributed and the α parameter was calculated with TREE-PUZZLE 5.2 [45]. Bootstrap analysis [46] with 1000 replicates was performed to assess the relative confidence on the topology obtained. A second tree was constructed following the Maximum-likelihood (ML) method [47], by using the PHYML 2.4.5 program [48] included in the MacGDE software package. The tree parameters were the same as those used for the NJ tree; in this case, the reliability of the inferred phylogeny was assessed by 500 bootstrap repetitions.

### Accession numbers of ADH sequences

The accession numbers of the vertebrate ADH sequences used in alignments and phylogenetic analyses are listed in Table 2, except for those of *X. laevis*, *X. tropicalis*, *A. carolinensis* and *P. sinensis* ADHs, which are provided in Tables 3 and 4.

**Table 2:** Accession numbers of vertebrate ADHs used in alignments and phylogenetic analyses

**Class**	**Organism**	**Protein**	**Accession**^**1**^
ADH1	*Homo sapiens* (human)	ADH1B1	P00325
	*Rattus norvegicus* (rat)	ADH1	P06757
	*Mus musculus* (mouse)	ADH1	P00329
	*Oryctolagus cuniculus* (rabbit)	ADH1	Q03505
	*Gallus gallus* (chicken)	ADH1	P23991
	*Struthio camelus* (ostrich)	ADH1	P80338
	*Coturnix japonica* (quail)	ADH1	P19631
	*Alligator mississippiensis* (alligator)	ADH1	P80222
	*Naja naja* (cobra)	ADH1	P80512
	*Uromastyx hardwickii* (spiny-tailed lizard)	ADH1A	P25405
	*Uromastyx hardwickii* (spiny-tailed lizard)	ADH1B	P25406
	*Rana perezi* (frog)	ADH1	P22797
ADH2	*Homo sapiens* (human)	ADH2	P08319
	*Callithrix jacchus* (marmoset)	ADH2	F7CDN6
	*Bos taurus* (bovine)	ADH2	A6QPF3
	*Rattus norvegicus* (rat)	ADH2	Q64563
	*Mus musculus* (mouse)	ADH2	Q9QYY9
	*Oryctolagus cuniculus* (rabbit)	ADH2A	O466649
	*Oryctolagus cuniculus* (rabbit)	ADH2B	O46650
	*Struthio camelus* (ostrich)	ADH2	P80468
ADH3	*Homo sapiens* (human)	ADH3	P11766
	*Rattus norvegicus* (rat)	ADH3	P12711
	*Mus musculus* (mouse)	ADH3	P28474
	*Oryctolagus cuniculus* (rabbit)	ADH3	O19053
	*Gallus gallus* (chicken)	ADH3	Q5ZK81
	*Uromastyx hardwickii* (spiny-tailed lizard)	ADH3	P80467
ADH4	*Homo sapiens* (human)	ADH4	P40394
	*Rattus norvegicus* (rat)	ADH4	P41682
	*Mus musculus* (mouse)	ADH4	Q64437
ADH5	*Homo sapiens* (human)	ADH5	P28332
	*Bos taurus* (bovine)	ADH5	Q2KII0
	*Rattus norvegicus* (rat)	ADH5	Q5XI95
	*Peromyscus maniculatus* (deer mouse)	ADH5	P41681
	*Oryctolagus cuniculus* (rabbit)	ADH5	G1SCD6
ADH6	*Equus caballus* (horse)	ADH6	F6UA46
	*Bos taurus* (bovine)	ADH6	Q0P581
	*Canis familaris* (dog)	ADH6	E2RHR8
	*Ailuropoda melanoleuca* (panda)	ADH6	G1L5H7
	*Rattus norvegicus* (rat)	ADH6A	D3ZT84
	*Rattus norvegicus* (rat)	ADH6B	GenBank:XP_003749455
	*Mus musculus* (mouse)	ADH6A	Q9D932
	*Mus musculus* (mouse)	ADH6B	GenBank:XP_003688830
ADH7	*Columba livia* (feral pigeon)	ADH7	P86883
	*Taeniopygia guttata* (zebra finch)	ADH7	GenBank:XP_002187852
	*Gallus gallus* (chicken)	ADH7	O42483
ADH8	*Rana perezi* (frog)	ADH8	O57380

The table lists the accession numbers for the protein sequences, other than those of *X. laevis, X. tropicalis, A. carolinensis* and *P. sinensis,* used in alignments and phylogenetic analyses. ^1^Accession numbers refer to UniProt database, unless otherwise indicated.

**Table 3:** **ADH forms in *Xenopus laevis *** **and *Xenopus tropicalis***

		***X. laevis***		***X. tropicalis***	
**Class**	**Gene**	**Accession**	**Expression**	**Accession**	**Expression**
ADH1	*ADH1A*			**HF569235**	Str.72514, 27790, 68808: intestine, oviduct, spleen, adipose tissue, liver, lung, stomach, tadpole
				Q5I0S0	
	*ADH1A1*	n.a.	CF521684 (GenBank): kidney		
	*ADH1A2*	Q6IRQ3	Xl.18983: dorsal lip, heart, kidney, blastula		
	*ADH1B*	Q6DKD6	Xl.80710: liver	**HF569236**	Str.15510, 69558: adipose tissue, head, intestine, limb, liver, lung, ovary, oviduct, tail, tailbud embryo, tadpole, metamorphosis
		O93331^1^	**Intestine, kidney, liver, stomach**	Q5I0R0	
			Intestine, kidney, liver, air sac, tadpole (pronephros, liver)^2^		
	*ADH1C*	Q6IRQ0	Xl.9060: kidney, fat body	**HF569237**	Str.33643, 83367: intestine, lung, ovary, skin
ADH2	*ADH2*	Not found	Not found	**HF569238**	Str.5773: heart, limb, skin, intestine, tadpole, metamorphosis
ADH3	*ADH3*	**AJ575267**	Xl.23916: brain, digestive, head, limb, ovary, spleen, testis, oocyte, gastrula, tadpole, metamorphosis	**HF569239**	Str.16550: head, heart, intestine, kidney, lung, ovary, oviduct, skin, spleen, testis, thymus, gastrula, neurula, tailbud embryo, tadpole
		Q4V813		Q5HZT1	
			**Liver, stomach, kidney, intestine, oocyte, ovary**		
ADH7	*ADH7*	n.a.	CB592872^3^ (GenBank): testis	**HF569240**	Str.27783, 70262, 73157: brain, gastrula, tadpole
				Q5M7K9	
ADH8	*ADH8A*	n.a.	Xl.21891: neurula	**HF569241**	Str.72032: head, tadpole
	*ADH8B*	**AJ566764**	Xl.53979: limb, metamorphosis	**HF569242**	Str.88986: stomach, skin, limb
		Q4R0Y8	**Esophagus, intestine, laringe, stomach, skin**		
ADH9	*ADH9*	Q7SYU6	Xl.21584: brain, metamorphosis	**HF569243**	
			**Esophagus, stomach**		
			Esophagus, skin, stomach^2^		
ADH10	*ADH10A*	Q6P7G1	Xl.81589, 34490: kidney, testis, metamorphosis	**HF569244**	Str.26581: kidney, head, intestine, liver, spleen, tailbud embryo, tadpole
	*ADH10B*	Q6AZL8	Xl.48167: kidney, testis	**HF569245**	

All *Xenopus* ADHs supported by evidence at the protein, transcript or gene level, are included, together with their accession numbers and expression sites. A single *ADH1A* gene exists in *X. tropicalis* while two genes are found in *X. laevis*. In contrast, no *X. laevis ADH2* sequence has been found to the date. Accession numbers are taken from UniProt or EMBL (all the EMBL sequences, in bold, are from this study). Expression sites were mostly obtained from UniGene (cluster numbers are provided), but also from GenBank (where indicated). For *X. laevis*, results of expression experiments from our group (in bold) and other sources, are also included. ^1^Partial sequence, probably another allele of this gene; ^2^Hoffmann *et al.*[22]; ^3^Partial sequence, containing frameshift mutations; n.a.: not available.

**Table 4:** **ADH forms in *A. carolinensis *** **and *P. sinensis***

	***A. carolinensis***		***P. sinensis***	
**Class**	**Gene**	**Accession**	**Gene**	**Accession**
ADH1	*ADH1A*	HF569253	*ADH1*	HF571257
	*ADH1B*	HF569252		
	*ADH1C*	HF569251		
	*ADH1D*	HF569250		
	*ADH1E*	HF569249		
	*ADH1F*	HF569248		
	*ADH1G*	HF569247		
	*ADH1H*	HF569246		
ADH2	Not found	Not found	*ADH2*	HF571258
ADH3	*ADH3*	HF569254	*ADH3*	HF571259
ADH7	*ADH7A*	HF569257	*ADH7*	HF571260
	*ADH7B*	HF569256		
	*ADH7C*	HF569255		

All the ADH classes and genes identified in *A. carolinensis* (anole lizard) and *P. sinensis* (turtle) genomes, together with their EMBL accession numbers (from this study), are included. Multiple *ADH1* and *ADH7 loci* exist in *A. carolinensis*, while no *ADH2* gene has been found. In contrast, *P. sinensis* has a single gene for each class.

## Results

### Isolation, cloning and identification of *X. laevis* cDNAs

With the initial aim of studying the amphibian NADP^+^-dependent ADH8 in the development model *X. laevis*, degenerated primers were designed, based on the *R. perezi* ADH8 sequence. By RT-PCR amplification from a *X. laevis* stomach cDNA pool, four cDNAs were cloned and sequenced, and, on the basis of amino acid sequence identity, they were assigned to ADH1, ADH3, ADH8 and a novel ADH9 class. ADH1, ADH3 and ADH8 were similar to their *R. perezi* orthologues and showed the typical residues of each class, while the low sequence identity values (<58%) between ADH9 and other classes pointed it to be a new class. The *ADH1* cDNA was likely to be an allele of the same gene which partial sequence had been reported by Hoffmann *et al. *[21], while ADH9 was identical to the alleged ADH4-like form reported in the same study. Our studies here indicate that *Xenopus* does not possess an ADH4 ortholog.

Since the *X. tropicalis* genome project provided reliable genomic data from this organism, a parallel study was conducted to identify *X. tropicalis ADH* genes, and the same nomenclature was used for the two *Xenopus* species. Some *ADH* genes appear to be closely related, probably encoding isozymes of the same enzymatic class. Therefore, our gene notation includes an Arabic number indicating the ADH class, followed by a capital letter corresponding to the encoded isozyme, assigned in ascending order by the gene location in the scaffold. For those genes that had been previously identified in *X. tropicalis* genomic studies [4], their names were conserved whenever it was possible. Moreover, putative duplicated genes in *X. laevis* relative to *X. tropicalis* were denoted with a “1” or “2” tag after the name field.

According to this nomenclature, subsequent screening of data banks retrieved the following *X. laevis* ADH sequences, as detailed in Table 3: Four ADH1 forms (1A1 -partial- and 1A2, corresponding to a single 1A form in *X. tropicalis*; 1B and 1C); one form each of ADH3, ADH8 and ADH9; two novel sequences that were named ADH10 (10A and 10B); and a partial sequence similar to that of chicken ADH7. The here cloned *X. laevis ADH3* and *ADH9* cDNAs were identical to the sequences retrieved while *ADH1* cDNA corresponded to ADH1B. The only *ADH8* transcript found in databases showed a nonsense mutation after codon 25 (a likely amplification or sequencing artifact) and notably differed from the cloned sequence from gastric tissue. Thus, this new sequence was considered to be a different gene, designed as *ADH8A*, while that previously cloned was named *ADH8B*.

Class assignment was mainly based on amino acid identities in pairwise comparisons with other vertebrate ADH enzymes (see Additional file 1), considering an intraclass identity >65% for amphibian ADH sequences (see Additional file 2). The presence of class-specific residues and phylogenetic relationships were also taken into account for *Xenopus* ADH7 and ADH10, which were considered as separate classes despite their high amino acid identities with ADH1 enzymes.

### Expression of *X. laevis* ADHs

**Figure 1:**
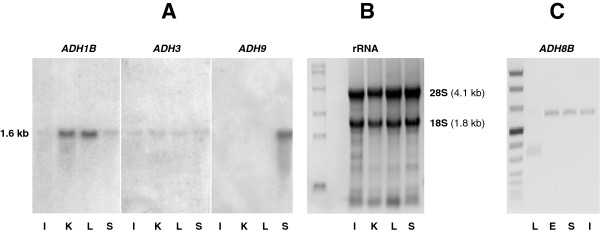
**Detection of ADH classes in *X. laevis*. (A)** Northern blot analysis of *ADH1B*, *ADH3* and *ADH9* from intestine (I), kidney (K), liver (L) and stomach (S), performed on 15-μg samples of total RNA. **(B)** Ethidium bromide-stained gel, from the same electrophoresis as in panel **(A)**, showing 18S and 28S rRNAs next to the RNA molecular weight marker (0.24-9.5 kb, Invitrogen). The estimated molecular size of the RNA hybrids detected was ~1.6 kb. **(C)** RT-PCR of *ADH8B* from liver (L), esophagus (E), stomach (S) and intestine (I) next to DNA molecular weight marker VIII (Roche). Esophagus, stomach and intestine show an amplification product of 603 bp, indicating the presence of the *ADH8B* cDNA.

**Figure 2:**
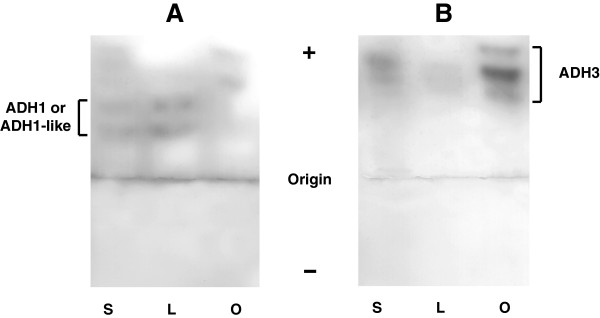
**Detection of ADH activity in *X. laevis *tissues.** Starch gel electrophoresis of tissue homogenates (15 μl) from different animals. **(A)** ADH1 or ADH1-like activity staining using 2-buten-1-ol as a substrate and NAD^+^ as a coenzyme. **(B)** Glutathione (GSH)-dependent formaldehyde dehydrogenase (ADH3) activity staining. Lanes: S, stomach; L, liver; O, ovary (pool of oocytes at different maturation stages). All detected ADH forms showed anodic mobility and different band patterns. ADH1 or ADH1-like activity is more abundant in liver extracts than in stomach and is absent in ovary, whereas ADH3 is more abundant in ovary.

Northern blot analysis performed on intestine, kidney, liver and stomach from *X. laevis*, with cDNA probes for cloned *ADH1B*, *ADH3* and *ADH9*, showed a 1.6-kb mRNA in positive samples (Figure 1A). *ADH3* transcripts were present in all the tissues analyzed at relatively low levels. Starch gel electrophoresis confirmed its generalized expression and revealed high glutathione-dependent formaldehyde dehydrogenase activity in the ovary as compared with esophagus, stomach and liver (Figure 2). *ADH1B* expression followed the typical pattern found for *ADH1* genes in vertebrates, as it was differentially expressed in the tissues analyzed (liver > kidney > stomach > intestine), while *ADH9* was only detected in stomach. Moreover, *ADH8B* transcripts were detected by RT-PCR in *X. laevis* esophagus, stomach and intestine but not in liver (Figure 1B), confirming the gastrointestinal location of this enzyme reported in *R. perezi *[15]. The diffuse band of low molecular weight observed in the liver corresponds to an unspecific amplification product, as confirmed by the lack of NADP^+^-dependent activity observed in electrophoresed liver extracts (not shown).

Additional data on the expression profile of the identified ADHs was obtained from *Xenopus* EST libraries. Although EST evidence is not quantitative, it can reflect very low transcript amounts, undetectable by less sensitive methods, but which may be physiologically relevant. Table 3 combines the results of localization studies with data obtained from expression libraries of adult tissues and embryonic stages.

### Chromosomal location and structure of *X. tropicalis*, *A. carolinensis* and *P. sinensis ADH* genes

A total of eleven *loci* encoding ADH enzymes (Table 3) were identified in scaffolds GL172747.1 and GL172865.1 of the *X. tropicalis* genome assembly 4.2. Scaffold GL172747.1 comprises the overlapping scaffolds 326 and 785 from previous version 3.0, referred in prior publications [4]. Scaffold GL172747.1 contains nine genes that constitute the main *ADH* cluster (from 2318883 to 2533660, spanning 215 kb) and one isolated gene (*ADH10A*: 2028408–2046244), while scaffold GL172865.1 contains another single *ADH* gene (*ADH1A*: 115684–136134), probably located in a different chromosome.

Likewise, twelve *ADH loci* (Table 4) were identified in scaffolds GL343323.1 and GL343307.1 of the *A. carolinensis* assembly AnoCar2.0. Scaffold GL343323.1 contains eleven genes (from 1237952 to 1438011, spanning 200 kb) ascribed to ADH1 and ADH7 classes; and a single gene is located in scaffold GL343307.1 (*ADH3*: 1468623–1476597). In the *P. sinensis* assembly PelSin_1.0, four *ADH loci* (Table 4) were identified in scaffolds JH210661.1 (*ADH3*: 581621–600183 and *ADH2*: 607230–649664), and JH209104.1 (*ADH7*: 16015–22889 and *ADH1*: 31847–53030). Therefore, although no *ADH2 locus* has been found in *A. carolinensis* (it was expected to localize between scaffolds GL343307.1 and GL343323.1), the analysis of the *P. sinensis* genome confirms the presence of class II in reptiles.

Sequence data of *X. laevis, X. tropicalis*, *A. carolinensis* and *P. sinensis* ADHs were deposited in the European Molecular Biology Laboratory (EMBL) nucleotide sequence database [49], and accession numbers are provided in Tables 3 and 4.

**Figure 3:**
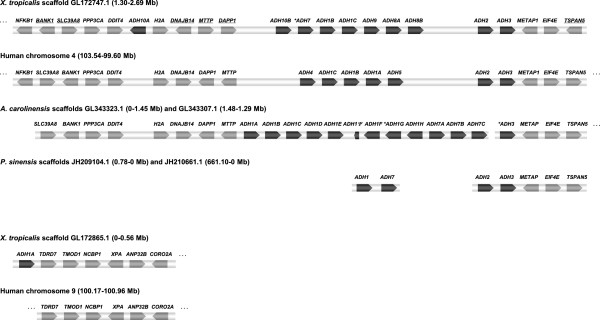
**Chromosomal location and synteny of *ADH loci.****X. tropicalis* scaffolds GL172747.1 and GL172865.1 are compared to human syntenic chromosomes 4 and 9, *A. carolinensis* scaffolds GL343323.1 and GL343307.1, and *P. sinensis* JH210661.1 and JH209104.1. All the identified genes are shown transcriptionally oriented (*ADH* genes in black and others in grey). The genes marked with an asterisk lack the first exon in the assembly. The opposite orientation of several orthologous genes (underlined) in *X. tropicalis* and human suggests a past inversion and posterior rearrangements involving the *ADH* cluster. In contrast, frog genes between *NPNT* (nephronectin, not shown) and *NFKB1*, located at 0.58-1.30 Mb of scaffold GL172747.1, have the same orientation as their human orthologues (not shown). Gene symbols in human chromosomes are *NFKB1*: Nuclear factor of kappa light polypeptide gene enhancer in B cells 1, *SLC39A8*: Solute carrier family 39 (zinc transporter) member 8, *BANK1*: B-cell scaffold protein with ankyrin repeats 1, *PPP3CA*: Serine/threonine phosphatase 2B catalytic subunit (alpha isoform), *DDIT4L*: DNA-damage inducible transcript 4-like, *H2AFZ*: Histone H2A family member Z, *DNAJB14*: DnaJ homolog subfamily B member 14, *MTTP*: Microsomal triglyceride transfer protein, *DAPP1*: Dual adaptor for phosphotyrosine and 3′-phosphoinositides, *METAP1*: Methionine aminopeptidase 1, *EIF4E*: Eukaryotic translation initiation factor 4E, *TSPAN5*: Tetraspanin 5, *TDRD7*: Tudor domain containing protein 7, *TMOD1*: Tropomodulin-1, *NCBP1*: 80 kDa nuclear cap binding protein, *XPA*: DNA-repair protein complementing XP-A cells, *ANP32B*: Acidic leucine-rich nuclear phosphoprotein 32 family member B, *CORO2A*: Coronin-2A.

Synteny of amphibian, reptile and human regions containing *ADH* genes is shown in Figure 3. Scaffolds GL172747.1 of *X. tropicalis*, GL343323.1 of *A. carolinensis*, and JH209104.1 and JH210661.1 of *P. sinensis* are syntenic to human chromosome 4, where all seven human *ADH* genes are closely linked. On the other hand, *X. tropicalis* scaffold GL172865.1 may be syntenic to human chromosome 9. Arrangement of several orthologous genes flanking the *ADH loci* in *X. tropicalis* scaffold GL172747.1 (the whole human syntenic region spans 2.7 kb) indicates a past inversion that isolated *ADH10A* from the main *ADH* cluster, presumably followed by other rearrangements in the same region. In contrast, reptiles show a single cluster although *ADH* genes are found in both orientations in the *A. carolinensis* genome.

All *X. tropicalis* genes contain nine exons and eight introns and the insertion points are those conserved in animal ADHs [50]. Comparison of the gene structures shows a wide range of intron sizes; therefore this parameter has not been conserved, even among close genes. The fact that no stop codons or reading frame alterations were detected in the coding sequences, together with evidence from *X. tropicalis* EST collections, support the idea that all these genes are transcriptionally active. Moreover, all the *X. tropicalis* ESTs found could be assigned to an *ADH* genomic sequence. The eleven genes were phylogenetically classified in seven classes: ADH1 (1A, 1B, 1C), 2, 3, 7, 8 (8A, 8B), 9, and 10 (10A and 10B). Furthermore, sequences from UniProt [28] provided supporting evidence and sometimes complementary information, like the first exon of *ADH7*, missing in the assembly but present in sequence Q5M7K9 (Table 3).

### Promoter analysis of *X. tropicalis ADH* genes

Regions including 600 bp upstream of the translation start codon were screened for proximal regulatory elements (see Additional file 3, Additional file 4, Additional file 5, Additional file 6, Additional file 7, Additional file 8, Additional file 9, Additional file 10, Additional file 11, Additional file 12 and Additional file 13 for *X. tropicalis* ADH cDNAs including predicted regulatory elements). Putative TATA boxes were established for all genes except for *ADH3*. Upstream TATA box has been described for most *ADH* genes, such as those encoding human and mouse class I [51-54] and human class II [55]; whereas the promoters of human and mouse class III contain GC boxes clustered near the start site [56-58].

Putative transcription factor binding sites were also predicted, although they should be functionally validated *in vivo* since competition, chromatin structure and other influences are as important as binding affinity. Promoters of *ADH1A* and *ADH1B* contain putative sites for HNF3beta, GATA-1, overlapping half-sites for estrogen and retinoid receptors (repeated twice in *ADH1B*), three AP1 sites in *ADH1A* and one site for Sp1 in *ADH1B*. Positive regulation of the human *ADH1A* gene was reported to be influenced by GATA-2, while differences in HNF3beta binding could be related with tissue specificity of ADH1 [59]. In addition, AP1-responsive genes are susceptible to be negatively regulated by retinoic acid [60]. *ADH1C* promoter has a single site for Oct1 and reverse sites for HNF3beta and Sp1. The *ADH2* promoter contains one NF1-binding site in forward orientation and two reverse sites for GATA-1, NF1 and AP1, and one for Gfi1. The *ADH3* TATA-less promoter shows single sites for Oct1, c-Myb and HNF3beta, a reverse CCAAT box and reverse sites for USF, GATA-1 and AP1. Both *ADH8* promoters exhibit a CCAAT box, and GATA-1 (two in *ADH8A*), XFD and HNF3beta sites. Their differential traits are CRE-BP1 and AP1 sites in *ADH8A*, and overlapping half-sites for estrogen and retinoid receptors in *ADH8B*. The *ADH9* promoter has also a CCAAT box, and GATA-1 and XFD sites, in addition to a site for Oct1. Finally, *ADH10A* promoter has one USF, one CHOP:C/EBPalpha and three HNF3beta sites, while *ADH10B* shows a putative site for estrogen receptor, and GATA-1 and AP1-binding sites.

Furthermore, single polyadenylation signals were found within the first 650 bp of the 3′-non-coding region of the three *ADH1* genes, *ADH3*, *ADH7* and *ADH8B*; two signals were observed in the case of *ADH2*, *ADH8A*, *ADH9* and *ADH10B*, and up to five signals were located at the 3′-end of *ADH10A*.

### Sequence analysis and evolutionary relationships

Available amino acid sequences of *R. perezi*, *X. laevis* and *X. tropicalis* were aligned (see Additional file 14) and key residues for substrate and coenzyme binding are summarized in Table 5.

**Table 5:** Substrate and coenzyme-interacting residues in amphibian ADHs

**Enzyme**	**Substrate-binding**											**Coenzyme-binding**								
	**Inner**				**Middle**				**Outer**											
	**48**	**93**	**140**	**141**	**57**	**116**	**294**	**318**	**110**	**306**	**309**	**47**	**48**	**51**	**223**	**224**	**225**	**269**	**271**	**363**
Xt-1A	S	F	F	V	M	I	V	L	V	M	L	R	S	H	D	T	N	I	N	H
Xl-1A1	S	F	F	V	I	I	-	-	M	-	-	R	S	H	D	T	N	-	-	-
Xl-1A2	S	F	F	M	F	L	V	L	M	M	L	G	S	T	D	T	N	V	N	H
Rp-1	S	F	F	I	L	I	L	V	L	L	L	R	S	H	D	L	N	I	N	R
Xl-1B	S	F	F	L	I	I	L	L	L	M	L	R	S	H	D	T	N	I	N	R
Xt-1B	S	F	F	L	I	I	L	L	L	M	L	R	S	H	D	T	N	I	N	R
Xl-1C	T	S	F	V	V	F	L	L	I	M	L	H	T	H	D	T	N	V	D	N
Xt-1C	T	S	F	V	V	F	L	L	I	M	L	H	T	H	D	T	N	V	D	T
Xt-2	T	Y	F	M	F	A	V	F	L	F	L	R	T	H	D	I	N	I	I	R
Xl-3	T	Y	F	M	D	I	V	A	L	F	V	H	T	Y	D	L	N	I	N	H
Xt-3	T	Y	F	M	D	I	V	A	L	F	V	H	T	Y	D	L	N	I	N	H
Xt-7	T	C	F	L	L	L	E	I	C	I	F	R	T	H	D	I	N	I	N	R
Xl-8A	S	F	Y	M	L	F	V	A	L	G	L	G	S	S	**G**	**S**	**Q**	T	Y	K
Xt-8A	S	F	F	M	L	F	V	A	V	G	L	G	S	S	**G**	**S**	**H**	T	Y	Q
Rp-8	S	F	L	V	I	M	L	V	F	L	L	G	S	S	**G**	**T**	**H**	A	R	S
Xl-8B	T	C	F	L	L	F	V	P	L	G	M	G	T	A	**G**	**S**	**H**	S	N	A
Xt-8B	T	C	F	V	L	F	V	P	L	G	M	G	T	A	**G**	**S**	**H**	S	N	A
Xl-9	T	C	F	M	M	V	V	F	F	M	L	H	T	H	D	I	N	V	K	R
Xt-9	T	C	F	M	M	V	V	F	F	I	L	H	T	H	D	I	N	V	N	R
Xl-10A	S	F	F	L	L	L	V	V	I	M	L	R	S	H	D	V	N	V	H	H
Xt-10A	S	F	F	M	L	M	V	V	M	M	L	R	S	H	D	I	N	V	H	R
Xl-10B	S	V	F	L	F	L	V	M	R	M	L	R	S	H	D	V	N	V	H	L
Xt-10B	S	V	F	M	F	L	A	S	R	M	L	R	S	H	D	I	N	V	H	H

All available sequences, full-length and partial, are considered. Sequence annotation consists of source organism abbreviation (Rp: *Rana perezi*, Xl: *Xenopus laevis*, Xt: *Xenopus tropicalis*) followed by the assigned class number. Position numbering is based on horse ADH1E. Residues at positions 223–225 (in bold) are involved in the interaction with the extra phosphate group of NADP^+^ in ADH8 and determine preference for this coenzyme [19].

*Xenopus* orthologs are closely related phylogenetically, with an intraclass identity of 85-97% (see Additional file 2). Evidence of *X. laevis* genome duplication was exclusively found for *X. tropicalis ADH1A*, which corresponds to *X. laevis ADH1A1* and *ADH1A2* genes (Table 3). Although only partial sequences were found for *X. laevis* ADH1A1, identical key residues in *X. tropicalis* ADH1A (Arg47, His51 and Val141), which are not found in *X. laevis* ADH1A2, suggest that the two former sequences would be functionally closer. The percentage of identity between *X. laevis* ADH1A1 and ADH1A2 is 89.3% in 244 residues.

When *Rana* and *Xenopus* class I sequences were compared, *R. perezi* ADH1 showed the highest identity with *Xenopus* ADH1B forms (~76%) and identical residues at most important positions (Table 5). For ADH8, the percent identity between the *R. perezi* and *Xenopus* forms is 71-73% and all possess Ser48, Ser51, Phe93, Gly223 and Leu309.

**Figure 4:**
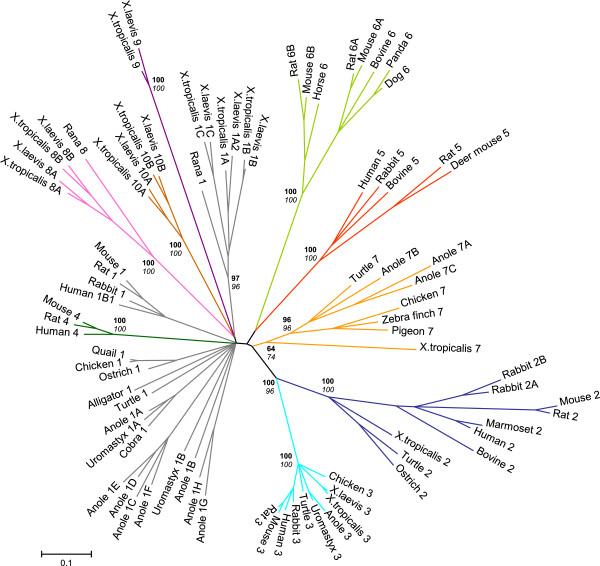
**Phylogeny of vertebrate ADHs.** Seven amphibian classes of ADH can be differentiated phylogenetically, where branches of each class are shown in a different color. The reliability of the Neighbour-joining (NJ) tree was tested by bootstrap analysis (1000 replicates). Within each class, branches were collapsed when bootstrap values were <80 with the exception of *X. tropicalis* ADH7. A second tree constructed following the Maximum-likelihood (ML) method (500 replicates) produced a similar topology. Figures at nodes are the scores from bootstrap resampling of the data, NJ values are in bold and ML values in italics. ADH sequences from *X. laevis*, *X. tropicalis*, *A. carolinensis* and *P. sinensis* are those described in the present manuscript and their accession numbers are provided in Tables 3 and 4. Accession numbers of other ADH sequences are compiled in Table 2. Alignment of all vertebrate ADHs included in the phylogenetic tree is presented in Additional file 15. Scale-bar represents substitutions per nucleotide.

Amphibian sequences were also compared to other vertebrate ADHs, including those identified in *A. carolinensis* (anole lizard) and *P. sinensis* (turtle), in unrooted phylogenetic trees (Figure 4). Molecular phylogenetic analysis on the deduced protein sequences using NJ and ML estimations produced similar topologies. For each tree construction, among-site rate heterogeneity was taken into account and confidence in each node was assessed by 1000 and 500 bootstrap replicates, respectively. In the tree of Figure 4, *Xenopus* ADH1, ADH2, ADH3, ADH7 and ADH8 sequences cluster with other extant members of their classes, whereas ADH9 branches separately. The tree topology pictures the constant nature of class III, in contrast to the other ADH classes. Related to class I, amphibian ADH1, ADH8, ADH9 and ADH10 form a protein cluster presumably derived from a common ancestor. An important radiation would have occurred in amphibians from a primitive *ADH1* gene, originating the four classes mentioned above, although the order and genes involved in each duplicatory event cannot be ascertained. Likewise, the presence of eight ADH1 and three ADH7 forms in *A. carolinensis* suggests that specific duplications could have occurred in lizards but not in turtles, as these organisms belong to different reptilian lineages. Percent identity within anole class I sequences ranges from 70.6% to 86.4%, and ADH1A and ADH1B show the highest identity with uromastyx (spiny-tailed lizard) ADH1A (90.4%) and ADH1B (85.0%), respectively. Within anole ADH7 sequences, the percent identity is around 71-78%.

Interestingly, multiple alignments reveal that all class I enzymes from reptiles to humans, as well as other classes derived from amniote ADH1, such as ADH4 and ADH6, show a deletion at position 60 with respect to amphibian ADH1 proteins and the remaining ADH classes (see Additional file 15).

Phylogenetic data supporting the results of this study (sequence alignment and phylogenetic tree of vertebrate ADHs) are available from the TreeBase repository (http://purl.org/phylo/treebase/phylows/study/TB2:S15368).

## Discussion

A total of eleven *ADH* genes with a conserved structure have been identified in the *X. tropicalis* genome, and grouped in seven enzyme classes: ADH 1 (1A, 1B, 1C), 2, 3, 7, 8 (8A, 8B), 9, and 10 (10A and 10B). These *loci* are distributed in two scaffolds, one containing the main discontinuous *ADH* cluster, syntenic to human 4q21-23, but broken by several rearrangements, and the other showing the single *ADH1A locus*. The amphibian ADH system represents a unique organization among tetrapods since sequencing data and comparative analysis of genomes describe single clusters in human, rat, mouse and chicken [4,61]. Genes similar to those of *X. tropicalis* have been identified in *X. laevis*, indicating that the multiplicity of ADH forms was present prior to the divergence of the two species. Duplication of the *X. laevis* genome (30 Mya) [62] affected a great number of gene families, such as those of globin or α-actin [63,64], but the only ADH duplicates found to date correspond to the *X. tropicalis ADH1A* gene, which were named *ADH1A1* and *ADH1A2* in *X. laevis*. This suggests that many *ADH* duplicated *loci* could have been lost. Nevertheless, further identification of other gene duplicates in *X. laevis* should not be discarded. In this regard, activity staining of hepatic ADHs revealed the existence of two polymorphic genes coding for ethanol dehydrogenase subunits that did not heterodimerize and were placed in separate genetic linkage groups [17].

In the following description of the amphibian ADH properties, we include functional features of forms not yet characterized, predicted from the wide information of the structure/function relationships available for the ADH family. However, the proposed functions have to be confirmed by the expression and kinetic characterization of the novel enzymes, especially ADH9 and ADH10.

### Ancient forms of vertebrate ADH classes in amphibians ADH1

Amphibian class ADH1 clusters with the novel amphibian classes ADH8, ADH9 and ADH10, since all of them derive from a primitive *ADH1* gene ancestor, also common to the amniote class I line. Later, *ADH1* duplications generated *ADH1A*, *1B* and *1C* (these duplications were independent from mammalian ADH1 duplications that generated human *ADH1A*, *1B* and *1C* after rodent/primate divergence); *ADH8A* and *8B*; and *ADH10A* and *10B* from their corresponding ancestors.

*Xenopus* ADH1A is the most similar to other vertebrate ADH1 enzymes, whereas *Xenopus* ADH1B shows the highest identity with *R. perezi* ADH1. *Xenopus* ADH1A and ADH1B show Arg47, His51, Phe93 and Phe140, typical class I residues that are associated with ethanol dehydrogenase activity. The substrate-binding pocket of *R. perezi* ADH1B is extremely hydrophobic and space-restricted, resulting in low *K*_m_ values for aliphatic alcohols, it has wide substrate specificity and is moderately active with retinoids [18]. In ADH1A, smaller substrate-binding residues Val141 and Val294 anticipate higher *K*_m_ values for this isozyme, and substitution by His363 (Arg in many class I enzymes) suggests an increased rate of NAD^+^ dissociation and higher *k*_cat_ values. *X. laevis* ADH1A2 has many atypical residues, such as Gly47 or Thr51, which suggest an alternative proton-relay pathway in comparison with all the other class I enzymes, showing His51. Moreover, voluminous residues Phe57, Met110 and Met141 would increase hydrophobicity and would narrow the substrate cleft even more than in ADH1B. These residue exchanges predict different substrate specificity and suggest that ADH1A2 may have acquired a new function after gene duplication, while ADH1A1 would have maintained the original one.

ADH1C has unique features among substrate-binding residues. At position 93, the lack of an aromatic ring expands the substrate cleft and permits the accommodation of large substrates, as in human ADH1A and chicken ADH7 [14,65,66]. In contrast, an unusual Phe116 would narrow the entrance, although still may allow productive binding of retinoids as occurs in *X. laevis* ADH8B [67]. These features predict that ADH1C binds large alcohol substrates better than ethanol. Substitutions in the coenzyme-binding site, in relation to amniote class I, are His47 instead of Arg (ADH1C has His residues at both positions 47 and 51), Asp271, and Asn/Thr363, which could weaken the coenzyme binding and increase *k*_cat_ values.

Expression pattern of amphibian class I in adult and embryonic tissues resembles that of other vertebrates, and transcripts of ADH1B are abundant in the developing tadpole (Table 3 and [22]).

Regarding reptiles, specific duplications of the *ADH1* gene occurred in lizards. Anole ADH1A is the ortholog of uromastyx ADH1A and clusters with other known reptilian and avian class I enzymes, while anole ADH1B is the ortholog of uromastyx ADH1B and clusters with the rest of anole class I forms. Thus, *A. carolinensis ADH1C*-*1H* genes may have arisen from further tandem duplications of *ADH1B* in this organism, although the existence of additional *ADH1* genes in uromastyx cannot be discarded.

Interestingly, *A. carolinensis* ADH1D, ADH1E and ADH1G share the residues Gln-Arg-Ser instead of the typical class I triad Asp-Ile-Gln at positions 223–225, which interact with the adenosine moiety of the coenzyme. Similar residues are found in NADP^+^-dependent MDR enzymes such as *Sulfolobus solfataricus* glucose dehydrogenase (Gln-Arg-Arg), *Xilella fastidiosa* cinnamyl alcohol dehydrogenase (Thr-Arg-Ser) or *Saccharomyces cerevisiae* ADH6 (Ser-Arg-Ser), among others, thus suggesting a higher preference for this coenzyme. Given the phylogenetic distance between these ADH1 forms and amphibian class ADH8, this suggests that the NADP^+^ specificity could have arisen at least twice during the vertebrate ADH evolution.

### ADH2

Amphibians are the most ancient organisms that possess a class II enzyme. Therefore, the duplicatory event that generated ADH2 from ADH3 can be placed between fish/tetrapod and amphibian/amniote splits, 450 to 360 Mya [68]. Moreover, *X. tropicalis* ADH2 already has the four-residue insertion, considered as the most distinctive trait of class II enzymes. The phylogenetic proximity of amphibian and avian ADH2 enzymes, in spite of the overall variability of this class, predicts similar structure and kinetic behaviour. The ostrich enzyme has been described as a mixed-class, structurally similar to mammalian class II but resembling class-I kinetic properties, since it is notably active with short-chain alcohol substrates such as ethanol.

Ostrich ADH2 shares 81.6%, 77.3% and 68.8% identity with turtle, frog and human ADH2, respectively. These four sequences show Ser/Thr48, Phe57, Tyr93, Leu110, Phe140, Val294, Ile/Leu309 and Phe318 in the substrate-binding site (Table 6); and also Ser115 and Ser128, which distinguish human class II from class I [69]. All the residues involved in the substrate interaction are almost identical in frog, turtle and ostrich ADH2. Moreover, the three enzymes show Arg47, Ser/Thr48, His51 and Ile269 at the coenzyme-binding site, the same residues which are found in human ADH1B1, concluding that the primitive class II forms may share common kinetic properties with class I enzymes.

**Table 6:** Substrate and coenzyme-interacting residues in amphibian, reptilian, avian and mammalian ADH2

**Enzyme**	**Substrate-binding**														**Coenzyme-binding**				
	**Inner**				**Middle**							**Outer**							
	**48**	**93**	**140**	**141**	**57**	**Insertion after 115**				**294**	**318**	**110**	**306**	**309**	**47**	**48**	**51**	**269**	**271**
Mouse ADH2	T	F	F	M	K	N	F	K	Y	A	F	L	V	I	P	**T**	N	A	T
Rat ADH2	T	F	F	M	K	N	F	K	Y	A	F	L	V	I	P	**T**	N	A	T
Rabbit ADH2A	S	Y	F	F	F	K	G	K	N	V	N	F	I	I	**R**	S	**H**	A	G
Rabbit ADH2B	S	Y	F	L	F	E	H	K	N	V	S	F	V	I	**R**	S	Y	A	G
Human ADH2	T	Y	F	F	F	N	L	K	S	V	F	L	E	I	H	**T**	S/T*	A	G
Marmoset ADH2	T	Y	F	L	F	N	L	K	N	V	F	F	E	I	H	**T**	T	A	G
Bovine ADH2	S	H	F	M	F	H	F	K	N	V	S	F	M	L	H	S	**H**	A	G
Ostrich ADH2	T	Y	F	M	F	K	I	K	T	V	F	L	M	I	**R**	**T**	**H**	**I**	N
Turtle ADH2	S	Y	F	M	F	K	I	K	T	V	F	L	M	I	**R**	S	**H**	**I**	N
*X. tropicalis* ADH2	T	Y	F	M	F	K	I	K	T	V	F	L	F	L	**R**	**T**	**H**	**I**	I
Human ADH1B1	T	F	F	L	L	-	-	-	-	V	V	Y	M	L	**R**	**T**	**H**	**I**	R

Human ADH1B1 is included for comparison with all available ADH2 enzymes. The four-residue insertion of class II enzymes is also shown. Residues of the coenzyme-binding site (in bold) are common to human ADH1B1. Residues at positions 223-224-225 are D-I-N in all cases. *Human ADH2 is polymorphic at position 51 and shows Ser or Thr [65].

Class II can be divided in two structurally and functionally distinct subgroups [70]. The first one exhibits a low activity with ethanol and comprises mouse and rat ADH2, both showing Pro47, and rabbit ADH2B, which lacks a His residue at both positions 47 or 51 (Table 6). In contrast, the second group is constituted by rabbit ADH2A and amphibian, reptilian and avian ADH2, all of them possessing His51; and human, marmoset and bovine ADH2, which show His47 (bovine ADH2 has His residues at both positions 47 and 51). These forms may share not only the ethanol dehydrogenase activity but also the ability of metabolizing retinoids, as reported for human ADH2 [71].

### ADH3

*X. laevis* and *X. tropicalis* ADH3 sequences show the 22 functionally important residues strictly conserved in class III enzymes from reptiles to mammals [72,73]. ADH3 is a glutathione-dependent formaldehyde dehydrogenase, as seen spectrophotometrically for the purified enzyme of *R. perezi*[18] and by activity staining of electrophoresed tissue homogenates from *R. perezi* and *X. laevis*. Expression of amphibian ADH3 is detected in every stage and tissue studied, although it is more abundant in some organs such as the ovary, suggesting that oocytes may store large amounts of maternal ADH3 for its later use during the embryonic development. The maternal origin of *ADH3* mRNA has been previously described in *Drosophila *[74] and zebrafish [75] embryos.

### ADH7

The novel reptilian genomic sequences supported the class assignment of *X. tropicalis* ADH7, sharing identity percentages of 71.2% and 67.4% with turtle ADH7 and anole ADH7B, respectively. As listed in Table 7, all ADH7 enzymes show Thr48, involved in the stereospecificity for secondary alcohols; a small residue such as Cys at position 93 (Pro in chicken), indicative of high *K*_m_ values for ethanol and correct positioning of steroid substrates [14]; Phe140 and Leu141 (in most sequences); and similar coenzyme-binding residues. Positions 112 to 126 are almost identical, and His115 and Trp142 are common for all ADH7 enzymes. These two positions were reported to affect the conformation of the loop 112–120, widening the entrance of the substrate-binding site and conferring to ADH7 the ability to oxidize large hydrophobic alcohols [14].

**Table 7:** Substrate and coenzyme-interacting residues in amphibian, reptilian and avian ADH7

**Enzyme**	**Substrate-binding**													**Coenzyme-binding**				
	**Inner**				**Middle**						**Outer**							
	**48**	**93**	**140**	**141**	**57**	**115**	**116**	**142**	**294**	**318**	**110**	**306**	**309**	**47**	**48**	**51**	**269**	**271**
*X. tropicalis* ADH7	T	C	F	L	L	H	L	W	E	I	C	I	F	R	T	H	I	N
Turtle ADH7	T	C	F	L	L	H	L	W	T	F	C	M	F	R	T	H	I	R
Anole ADH7A	T	C	L	M	F	H	F	W	E	C	C	V	I	R	T	H	I	L
Anole ADH7B	T	C	F	L	F	H	L	W	V	F	C	M	F	G	T	H	V	C
Anole ADH7C	T	C	F	L	F	H	I	W	A	L	C	L	L	R	T	N	I	R
Chicken ADH7	T	P	F	L	F	H	L	W	V	L	Y	F	L	H	T	H	I	T
Zebra finch ADH7	T	C	Y	L	L	H	F	W	V	L	Y	V	L	H	T	H	I	H
Pigeon ADH7	T	C	F	M	I	H	F	W	V	L	Y	L	L	R	T	H	I	R

Residues 115 and 142, involved in the rearrangement of the loop 112–120 at the entrance of the substrate-binding pocket and present in all ADH7 forms, are also included. Residues at positions 223-224-225 are D-I-N in all cases.

Among the three forms identified in *A. carolinensis*, ADH7B is the most similar to turtle, chicken and frog ADH7, with identity percentages of 79.4%, 73.6% and 67.4%, respectively. Already present in amphibians, ADH7 appeared between the tetrapod/fish and the amniote/amphibian splits, 450–360 Mya [61]. On the other hand, the common position of turtle and chicken *ADH7* and vertebrate *ADH5-ADH6 loci* within the ADH cluster [4,13,61], together with the fact that they do not coexist in any organism, suggests that the loss of *ADH7* might have occurred close to the origin of *ADH5* and *ADH6*, or even in the same genetic event.

### Novel ADH classes in amphibians ADH8

A total of five members are now known from the NADP^+^-dependent class VIII: ADH8 from *R. perezi*, and ADH8A and ADH8B from both *X. laevis* and *X. tropicalis*. They show the conserved triad Gly223-Ser/Thr224-Gln/His225 that interacts with the 2′-phosphate of the adenosine moiety of NADP^+ ^[19]. Despite their different substrate-binding sites, especially concerning large hydrophobic residues, both *X. laevis* ADH8B and *R. perezi* ADH8 reduce retinaldehyde and medium-chain aldehydes [15,67]. One major difference is the substitution of Phe93 by Cys that correlates with the poor ethanol oxidizing activity of *X. laevis* ADH8B (Borràs *et al.*, unpublished results). Residues Gly47, Ser48 and Ser51 would determine the proposed catalytic mechanism and proton-relay pathway of *R. perezi* ADH8 [21]. While Gly47 is conserved in all ADH8 members, Ser48 and Ser51, also found in ADH8A, are substituted by Thr48 and Ala51 in ADH8B enzymes. Remarkably, several deletions exist in ADH8 with respect to amphibian class I. The first one is found at position 57 of *R. perezi* ADH8 and both *Xenopus* ADH8B enzymes, and it may account to some extent for the wide substrate pocket observed in *R. perezi* ADH8 [21]. The second one, at position 167, is common for all ADH8 sequences. And the third, located at position 186, is only found in *Xenopus* ADH8B enzymes. Regarding coenzyme-interacting positions, the presence of Gly47 and the lack of a typical hydrophobic residue in position 269, usually Val or Ile, suggest a lower affinity for the coenzyme and increased *k*_cat_ values for ADH8 enzymes. Adult expression pattern of ADH8 includes the gastrointestinal tract and skin, where it may participate in cell differentiation through regulation of retinoic acid levels acting as a retinaldehyde reductase [15]. In embryos, ADH8 expression is also observed after neurulation, although EST evidences are too scarce to suggest a possible function (Table 3). *ADH8A* and *ADH8B* promoters are closely related, both exhibiting a putative CCAAT box, and common HNF-3beta, XFD and GATA-binding sites.

### ADH9

*X. laevis* and *X. tropicalis* ADH9 are the only members known from this novel class, and none of them has been characterized at the protein level. These enzymes share a percentage of amino acid identity lower than 60% with any other ADH, and show many special sequence features, such as Cys93, Met57, Phe110, Val116, Met141, and Phe318; together with His residues at both positions 47 and 51. His51 indicates the same proton-relay pathway as in class I enzymes, while His47 suggests rapid coenzyme dissociation. Asp223 at the cofactor-binding site indicates NAD^+^-dependence. Cys93, Phe110, Met141, and Phe318 predict a substrate pocked enlarged at its inner part, but narrow and hydrophobic at the middle and the entrance, similar to that of ADH8. Substrate preferences may be large substrates rather than ethanol, but probably not steroids. Northern blot studies detect *ADH9* transcripts in adult stomach, esophagus and skin, while discard the expression of the enzyme at embryonic stages (Figure 1 and [22]). Colocalization with ADH8, in spite of their sequence divergence and different cofactor specificity, may obey to the common regulatory elements found in their gene promoters, and it is consistent with the adjacent chromosomal location of their genes. ADH9 was initially described as an ADH4-like form in *X. laevis*, likely because its tissue localization [22]. Based on phylogenetic analyses and class-specific sequence signatures, it is now clear that ADH9 constitutes a separate class and thus ADH4 is not present in amphibians. The absence of ADH4 forms in reptiles and birds, and its presence in marsupials [13] supports its emergence at the origin of mammals (310 Mya).

### ADH10

Two isoenzymatic forms occur in this class, ADH10A and ADH10B, which are closely related to ADH1. Common residues of ADH10 and ADH1 enzymes are Ser48, Phe140, Met306 and Leu309, for substrate interaction; and Arg47, His51 and Leu363, for coenzyme binding (Table 5). Val269 and His271 are particular of ADH10. Substitution of typical Ile269 by smaller Val can affect the strength of coenzyme interaction, and substitution of Arg271 by His was suggested to increase *k*_cat_ values in human ADH4 [76]. Three residue exchanges characterize ADH10B: Val93, Phe57 and Arg110. Val93 results in a wider bottom of the substrate pocket and it has not been found in any other ADH. Phe57, present in most class II enzymes, narrows the middle region and increases its hydrophobicity, but hydrophilic Arg110 should compensate for this fact. ADH10 is the only class with a charged residue at position 110. Also interesting is the basic residue found at position 115 which, similarly to Arg115 in class III enzymes [73], could contribute to a substrate-binding site with a wider entrance and higher volume. A deletion at position 114 of *X. tropicalis* ADH10B could also participate in this rearrangement. The substrate pocket of ADH10B with a widened entrance and inner part (Ser48 instead of Thr) could accommodate large substrates, such as steroids, provided that Phe57 was not a major steric constraint. Ser48 can also be found in horse ADH1S and human ADH1C, both able to oxidize steroids. Adrenal and gonadal steroids are therefore proposed as ADH10 substrates, in agreement with its predominant expression in *Xenopus* mesonephric kidney and testis, and the presence of a putative site for estrogen receptor in the *ADH10B* promoter.

## Conclusions

**Figure 5:**
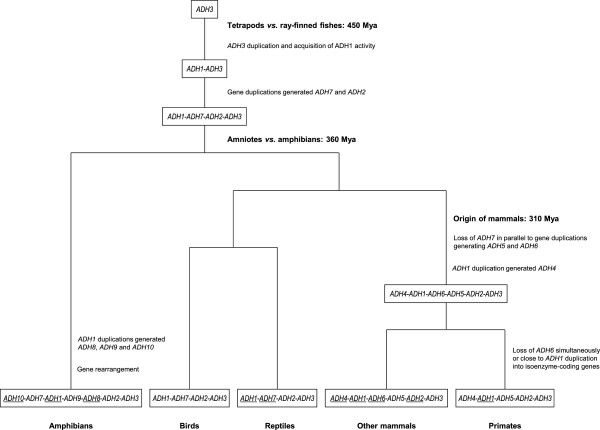
**Hypothetical evolutionary pathways leading to tetrapod ADH multiplicity.** At the base of vertebrate radiation, an initial tandem duplication of the ancestral *ADH3* led to a two-gene cluster. Actinopterygia (ray-finned fish) and sarcopterygia (lobe-finned fish and tetrapods) acquired ADH1 activity by the most 5′ member of the cluster [4]. Before the amniota/amphibian split (360 Mya), ADH2 and ADH7 would have arisen in tetrapods as a consequence of gene duplication events. In reptiles and birds, no additional ADH classes have been found. In contrast, *ADH1* tandem duplications led to further class multiplicity in the amphibian lineage; thus, *ADH8*, *ADH9*, and more recently *ADH10* forms would derive from the ancestral *ADH1*. Close to the origin of mammals, *ADH7* was lost while gene duplications generated *ADH5* and *ADH6*, and tandem duplication of *ADH1* gave rise to *ADH4*. Only in primates, ADH6 was lost simultaneously or close to *ADH1* duplications generating ADH1A-C isozymes [13]. Likewise, additional duplications occurred in other vertebrate lineages, and those *ADH* genes leading to isoenzyme multiplicity in at least one member of that lineage are underlined (in reptiles, multiple *ADH1* and *ADH7* are found in lizards, but not in turtles). In some organisms, ADH pseudogenes are also observed.

In conclusion, the complex *Xenopus* ADH system is composed of the vertebrate classes ADH1, ADH2, ADH3 and ADH7, together with novel class I-derived enzymes, ADH8, ADH9 and ADH10, exclusively found in amphibians. ADH4 is not present in amphibians and reptiles. The study of the ancient forms of ADH2 and ADH7, also found in reptiles, led to significant conclusions about the evolutionary history of the ADH family (Figure 5), whereas the special features showed by the novel forms described herein point to the acquisition of new functions following the *ADH* gene family expansion occurred in amphibians.

## Availability of supporting data

The data sets supporting the results of this article are included within the article and its additional files.

## Abbreviations

ADH: Alcohol dehydrogenase; AP1: Activator protein 1; cAMP: Cyclic adenosine monphosphate; C/EBPalpha: CCAAT-enhancer binding protein alpha; CHOP: C/EBP homologous protein; c-Myb: Mieloblastosis viral oncogene homolog; CRE-BP1: cAMP-responsive element binding protein; EST: Expressed sequence tag; FDH: Formaldehyde dehydrogenase; GATA-1: GATA-binding protein 1; Gfi1: Growth factor independence 1; HNF3beta: Hepatic nuclear factor 3 beta; MDR: Medium-chain dehydrogenase/reductase; NAD(P): Nicotinamide adenine dinucleotide (phosphate); NF1: Nuclear factor 1; Oct1: Octamer-binding factor 1; RT-PCR: Reverse transcription-polymerase chain reaction; SDS: Sodium dodecyl sulfate; USF: Upstream stimulating factor; Ssp1: Stimulating protein 1; XFD: *Xenopus* fork head domain.

## Competing interests

The authors declare that they have no competing interests.

## Authors’ contributions

EB carried out the cloning and detection of *X. laevis* ADH forms, identified ADH sequences in genomic and expression databases, performed comparative analyses, constructed NJ trees, and drafted the manuscript. RA calculated evolutionary parameters of vertebrate ADH, constructed ML trees, and participated in drafting of the manuscript. GD provided partial sequences of *X. laevis ADH8B* and *ADH9* cDNAs, and critically reviewed the manuscript. XP participated in the design and coordination of the study. JF conceived the study, participated in its design and coordination, and provided critical review of the manuscript. All authors have read and approved the final manuscript.

## Supplementary Material

Additional file 1 **Percentage of amino acid identity between *Xenopus tropicalis *** **and representative vertebrate ADH sequences.** Click here for file

Additional file 2 Percentage of amino acid identity between amphibian ADH sequences. Click here for file

Additional file 3 ***Xenopus tropicalis ADH1A *cDNA sequence.** The sequence includes the translated coding exons, intron flanking regions (±15 bp with total intron size), proximal promoter (-600 bp from the ATG codon) and 3′-untranslated region (650 bp) with predicted regulatory elements. Putative TATA boxes and polyadenylation signals are in bold and underlined. Putative transcription factor binding sites are underlined, with the core sequence of the matrix in bold and italics (for overlapping sites, the most downstream site is overlined); and the orientation (+ or - strand) is given in parentheses. Click here for file

Additional file 4 ***Xenopus tropicalis ADH1B *cDNA sequence.** The sequence includes the translated coding exons, intron flanking regions (±15 bp with total intron size), and the proximal promoter (-600 bp from the ATG codon) and 3′-untranslated region (650 bp) with predicted regulatory elements. Putative TATA boxes and polyadenylation signals are in bold and underlined. Putative transcription factor binding sites are underlined, with the core sequence of the matrix in bold and italics (for overlapping sites, the most downstream site is overlined); and the orientation (+ or - strand) is given in parentheses. Click here for file

Additional file 5 ***Xenopus tropicalis ADH1C *cDNA sequence.** The sequence includes the translated coding exons, intron flanking regions (±15 bp with total intron size), and the proximal promoter (-600 bp from the ATG codon) and 3′-untranslated region (650 bp) with predicted regulatory elements. Putative TATA boxes and polyadenylation signals are in bold and underlined. Putative transcription factor binding sites are underlined, with the core sequence of the matrix in bold and italics (for overlapping sites, the most downstream site is overlined); and the orientation (+ or - strand) is given in parentheses. Click here for file

Additional file 6 ***Xenopus tropicalis ADH2 *cDNA sequence.** The sequence includes the translated coding exons, intron flanking regions (±15 bp with total intron size), and the proximal promoter (-600 bp from the ATG codon) and 3′-untranslated region (650 bp) with predicted regulatory elements. Putative TATA boxes and polyadenylation signals are in bold and underlined. Putative transcription factor binding sites are underlined, with the core sequence of the matrix in bold and italics (for overlapping sites, the most downstream site is overlined); and the orientation (+ or - strand) is given in parentheses. Click here for file

Additional file 7 ***Xenopus tropicalis ADH3 *cDNA sequence.** The sequence includes the translated coding exons, intron flanking regions (±15 bp with total intron size), and the proximal promoter (-600 bp from the ATG codon) and 3′-untranslated region (650 bp) with predicted regulatory elements. Putative TATA boxes and polyadenylation signals are in bold and underlined. Putative transcription factor binding sites are underlined, with the core sequence of the matrix in bold and italics (for overlapping sites, the most downstream site is overlined); and the orientation (+ or - strand) is given in parentheses. Click here for file

Additional file 8 ***Xenopus tropicalis ADH7 *cDNA sequence.** The sequence includes the translated coding exons, intron flanking regions (±15 bp with total intron size), and 3′-untranslated region (650 bp) with predicted regulatory elements. Putative TATA boxes and polyadenylation signals are in bold and underlined. Click here for file

Additional file 9 ***Xenopus tropicalis ADH8A *cDNA sequence.** The sequence includes the translated coding exons, intron flanking regions (±15 bp with total intron size), and the proximal promoter (-600 bp from the ATG codon) and 3′-untranslated region (650 bp) with predicted regulatory elements. Putative TATA boxes and polyadenylation signals are in bold and underlined. Putative transcription factor binding sites are underlined, with the core sequence of the matrix in bold and italics (for overlapping sites, the most downstream site is overlined); and the orientation (+ or - strand) is given in parentheses. Click here for file

Additional file 10 ***Xenopus tropicalis ADH8B *cDNA sequence.** The sequence includes the translated coding exons, intron flanking regions (±15 bp with total intron size), and the proximal promoter (-600 bp from the ATG codon) and 3′-untranslated region (650 bp) with predicted regulatory elements. Putative TATA boxes and polyadenylation signals are in bold and underlined. Putative transcription factor binding sites are underlined, with the core sequence of the matrix in bold and italics (for overlapping sites, the most downstream site is overlined); and the orientation (+ or - strand) is given in parentheses. Click here for file

Additional file 11 ***Xenopus tropicalis ADH9 *cDNA sequence.** The sequence includes the translated coding exons, intron flanking regions (±15 bp with total intron size), and the proximal promoter (-600 bp from the ATG codon) and 3′-untranslated region (650 bp) with predicted regulatory elements. Putative TATA boxes and polyadenylation signals are in bold and underlined. Putative transcription factor binding sites are underlined, with the core sequence of the matrix in bold and italics (for overlapping sites, the most downstream site is overlined); and the orientation (+ or - strand) is given in parentheses. Click here for file

Additional file 12 ***Xenopus tropicalis ADH10A *cDNA sequence.** The sequence includes the translated coding exons, intron flanking regions (±15 bp with total intron size), and the proximal promoter (-600 bp from the ATG codon) and 3′-untranslated region (650 bp) with predicted regulatory elements. Putative TATA boxes and polyadenylation signals are in bold and underlined. Putative transcription factor binding sites are underlined, with the core sequence of the matrix in bold and italics (for overlapping sites, the most downstream site is overlined); and the orientation (+ or - strand) is given in parentheses. Click here for file

Additional file 13 ***Xenopus tropicalis ADH10B *cDNA sequence.** The sequence includes the translated coding exons, intron flanking regions (±15 bp with total intron size), and the proximal promoter (-600 bp from the ATG codon) and 3′-untranslated region (650 bp) with predicted regulatory elements. Putative TATA boxes and polyadenylation signals are in bold and underlined. Putative transcription factor binding sites are underlined, with the core sequence of the matrix in bold and italics (for overlapping sites, the most downstream site is overlined); and the orientation (+ or - strand) is given in parentheses. Click here for file

Additional file 14 Alignment of amphibian ADH amino acid sequences. Click here for file

Additional file 15 Alignment of vertebrate ADHs included in the phylogenetic tree. Click here for file
