# Supplementary material for: The Xenopus alcohol dehydrogenase gene family: characterization and comparative analysis incorporating amphibian and reptilian genomes
Source: BMC Genomics. 2014 Mar 20;15:216. doi: 10.1186/1471-2164-15-216 (PMC4028059; doi:10.1186/1471-2164-15-216)
Supplement: Additional file 1 — Percentage of amino acid identity between Xenopus tropicalis and representative vertebrate ADH sequences. [file 1471-2164-15-216-S1.doc]

Percentage of amino acid identity between *Xenopus tropicalis* and representative vertebrate ADH sequences. Highest values within each class are shown in bold. Representative values are shaded.

|  | *Xenopus tropicalis* ADH classes | | | | | | |
| --- | --- | --- | --- | --- | --- | --- | --- |
|  | ADH1 | ADH2 | ADH3 | ADH7 | ADH8 | ADH9 | ADH10 |
| *Rana perezi* ADH1 | **67.5-75.5** | 61.7 | 61.6 | **62.1** | 56.8-59.4 | **59.2** | **62.4-65.6** |
| Alligator ADH1 | **62.5-66.4** | 66.4 | 64.8 | **66.1** | 57.7-60.2 | **57.3** | **62.6-65.8** |
| Ostrich ADH1 | **64.3-69.3** | 67.0 | 65.8 | **64.8** | 57.6-60.2 | **59.2** | **64.8-68.0** |
| Human ADH1B1 | **63.3-66.9** | 64.1 | 62.6 | **65.8** | 58.1-58.4 | **59.4** | **63.2-64.2** |
| Ostrich ADH2 | 57.7-59.8 | **77.3** | 67.2 | 61.7 | 58.0-58.3 | 53.0 | 59.6-60.6 |
| Human ADH2 | 53.7-54.3 | 68.8 | 63.0 | 58.0 | 58.0-59.6 | 52.5 | 55.6-57.2 |
| Human ADH3 | 57.7-60.4 | 67.5 | **85.8** | **64.0** | 55.4-56.8 | 56.0 | 59.4-59.7 |
| Human ADH4 | 59.1-63.4 | 61.4 | 61.6 | 61.3 | 56.0-57.3 | 52.8 | 61.6-62.6 |
| Human ADH5 | 57.0-59.2 | 59.6 | 59.2 | 55.7 | 55.7-56.5 | 53.3 | 56.2-57.8 |
| Rat ADH6 | 49.3-52.0 | 53.2 | 52.8 | 53.6 | 50.6-52.5 | 50.1 | 51.7-53.0 |
| Chicken ADH7 | 58.5-62.6 | 64.3 | 63.4 | **64.0** | 54.1-54.6 | 54.1 | 58.9 |
| *Rana perezi* ADH8 | 55.6-57.8 | 56.4 | 55.4 | 56.0 | **67.0-71.3** | 54.6 | 57.6-58.1 |
