# Supplementary material for: The Xenopus alcohol dehydrogenase gene family: characterization and comparative analysis incorporating amphibian and reptilian genomes
Source: BMC Genomics. 2014 Mar 20;15:216. doi: 10.1186/1471-2164-15-216 (PMC4028059; doi:10.1186/1471-2164-15-216)
Supplement: Additional file 2 — Percentage of amino acid identity between amphibian ADH sequences. [file 1471-2164-15-216-S2.doc]

**Percentage of amino acid identity between amphibian ADH sequences.** All available full-length sequences are included. Sequence names consist of source organism abbreviation (Rp: *Rana perezi*, Xl: *Xenopus laevis*, Xt: *Xenopus tropicalis*) followed by the assigned class number and enzyme notation. Intraclass percentages of identity are shown in bold and shaded.

| **Xl-3** | **Xt-2** | **Rp-1** | **Xt-1B** | **Xl-1B** | **Xt-1A** | **Xl-1A2** | **Xt-1C** | **Xl-1C** | **Rp-8** | **Xt-8B** | **Xl-8B** | **Xl-8A** | **Xt-8A** | **Xt-9** | **Xl-9** | **Xt-10B** | **Xl-10B** | **Xt-10A** | **Xl-10A** | **Xt-7** |  |
| --- | --- | --- | --- | --- | --- | --- | --- | --- | --- | --- | --- | --- | --- | --- | --- | --- | --- | --- | --- | --- | --- |
| **96.8** | 69.3 | 61.6 | 59.9 | 60.7 | 61.8 | 61.7 | 58.5 | 57.6 | 55.4 | 55.2 | 54.6 | 57.8 | 56.8 | 54.9 | 52.8 | 58.9 | 58.5 | 60.2 | 59.2 | 64.2 | **Xt-3** |
|  | 69.3 | 62.6 | 61.2 | 61.5 | 62.9 | 62.2 | 58.8 | 58.1 | 56.2 | 55.7 | 54.6 | 58.4 | 57.3 | 55.4 | 53.3 | 58.6 | 58.3 | 59.7 | 59.2 | 63.7 | **Xl-3** |
|  |  | 61.7 | 61.4 | 61.1 | 63.0 | 60.7 | 61.0 | 60.4 | 56.7 | 57.7 | 59.3 | 59.3 | 59.1 | 55.1 | 53.8 | 61.4 | 61.0 | 62.5 | 61.4 | 62.7 | **Xt-2** |
|  |  |  | **75.5** | **75.8** | **71.7** | **69.1** | **67.5** | **68.8** | 60.5 | 57.0 | 56.8 | 59.7 | 59.7 | 59.2 | 57.6 | 62.4 | 62.7 | 65.6 | 65.0 | 62.1 | **Rp-1** |
|  |  |  |  | **94.1** | **75.0** | **74.2** | **69.9** | **70.0** | 57.8 | 55.1 | 54.3 | 57.2 | 59.4 | 55.9 | 56.7 | 62.0 | 60.9 | 64.1 | 62.8 | 61.8 | **Xt-1B** |
|  |  |  |  |  | **76.1** | **75.0** | **70.1** | **70.8** | 58.8 | 57.2 | 55.7 | 58.0 | 59.9 | 57.2 | 57.5 | 62.0 | 60.9 | 63.6 | 63.1 | 63.1 | **Xl-1B** |
|  |  |  |  |  |  | **82.9** | **71.5** | **72.2** | 57.0 | 54.4 | 54.1 | 56.2 | 57.0 | 58.4 | 56.5 | 64.2 | 62.5 | 65.0 | 65.0 | 62.1 | **Xt-1A** |
|  |  |  |  |  |  |  | **71.2** | **70.4** | 56.1 | 54.5 | 53.9 | 55.5 | 56.3 | 58.7 | 57.9 | 61.7 | 60.9 | 61.4 | 61.4 | 60.9 | **Xl-1A2** |
|  |  |  |  |  |  |  |  | **87.0** | 55.6 | 54.0 | 54.0 | 55.6 | 54.6 | 55.1 | 54.3 | 59.8 | 59.6 | 62.0 | 61.2 | 58.0 | **Xt-1C** |
|  |  |  |  |  |  |  |  |  | 57.8 | 54.9 | 55.2 | 56.5 | 56.5 | 57.3 | 56.8 | 61.3 | 60.6 | 62.1 | 61.6 | 58.4 | **Xl-1C** |
|  |  |  |  |  |  |  |  |  |  | **67.0** | **66.2** | **72.7** | **71.3** | 54.4 | 53.6 | 57.6 | 56.2 | 58.1 | 58.4 | 56.0 | **Rp-8** |
|  |  |  |  |  |  |  |  |  |  |  | **85.4** | **78.0** | **80.7** | 52.0 | 50.6 | 55.4 | 55.9 | 57.0 | 56.8 | 56.0 | **Xt-8B** |
|  |  |  |  |  |  |  |  |  |  |  |  | **76.4** | **78.6** | 52.5 | 50.9 | 56.0 | 56.2 | 56.2 | 56.5 | 55.7 | **Xl-8B** |
|  |  |  |  |  |  |  |  |  |  |  |  |  | **88.7** | 54.4 | 53.0 | 58.4 | 57.5 | 60.2 | 59.4 | 55.7 | **Xl-8A** |
|  |  |  |  |  |  |  |  |  |  |  |  |  |  | 52.8 | 52.0 | 59.2 | 58.8 | 61.0 | 60.5 | 57.8 | **Xt-8A** |
|  |  |  |  |  |  |  |  |  |  |  |  |  |  |  | **90.1** | 56.0 | 53.8 | 56.5 | 56.5 | 57.6 | **Xt-9** |
|  |  |  |  |  |  |  |  |  |  |  |  |  |  |  |  | 55.7 | 53.0 | 56.0 | 56.0 | 56.5 | **Xl-9** |
|  |  |  |  |  |  |  |  |  |  |  |  |  |  |  |  |  | **87.7** | **80.5** | **78.4** | 61.6 | **Xt-10B** |
|  |  |  |  |  |  |  |  |  |  |  |  |  |  |  |  |  |  | **78.8** | **79.4** | 59.8 | **Xl-10B** |
|  |  |  |  |  |  |  |  |  |  |  |  |  |  |  |  |  |  |  | **88.2** | 61.6 | **Xt-10A** |
|  |  |  |  |  |  |  |  |  |  |  |  |  |  |  |  |  |  |  |  | 62.1 | **Xl-10A** |
