# Supplementary material for: The Xenopus alcohol dehydrogenase gene family: characterization and comparative analysis incorporating amphibian and reptilian genomes
Source: BMC Genomics. 2014 Mar 20;15:216. doi: 10.1186/1471-2164-15-216 (PMC4028059; doi:10.1186/1471-2164-15-216)
Supplement: Additional file 3 — Xenopus tropicalis ADH1A cDNA sequence. The sequence includes the translated coding exons, intron flanking regions (±15 bp with total intron size), proximal promoter (-600 bp from the ATG codon) and 3′-untranslated region (650 bp) with predicted regulatory elements. Putative TATA boxes and polyadenylation signals are in bold and underlined. Putative transcription factor binding sites are underlined, with the core sequence of the matrix in bold and italics (for overlapping sites, the most downstream site is overlined); and the orientation (+ or - strand) is given in parentheses. [file 1471-2164-15-216-S3.doc]

***X. tropicalis ADH1A***

**-600**

ATATTGCAGTGTCACCCACTG***TGACC***TACAGCACTTATATTTGCCTATTTGTGTCTGTAAGTTACCCTCCCATATAGATTGTAAGCTCTACGGGGCAGGAACCTCCA

ER(+) RORA1(-)

TCCTCTTGTGTTTT***TGACT***CTTATTGCAACTG***TATCT***TT***TATTT***ATTTGTCTTTATTGTAATACTTTGTAT***TTATC***TA***TTATC***TTAATAACCCCC***TGTTT***GTATTAA

AP1(+) GATA1(-) HNF3B(+) GATA1(-) GATA1(-) HFH3(+)

TGTATTCTACTGTACAGCGTACAGTACATAAGTAGCGCTTT***ATAAA***TAAA***GATA***TACATACATACATACATACATACATACATCAGTATAT***TGACT***TAAATCTATTT

XFD2(+) GATA1(+) AP1(+)

CCCTCCCTACTCATTTTGAC***ACCTG***TCTGAGCACACAGATTAAAAAAAGTATGCACAAATGCCAAGTTTTGTGCACATGAATAAGAA***AAATA***TTATAGGGAACCTTT

MYOD(-) HNF3B(-)

CCACCAGAATAGCAGGACATGTATTTTCTTGGTGTTGCTTTAAGATTGAGAAACACCATAATTCCACCCTGCAGGAGGGGTTGTGGTTCTT**TATA*TGACT***CCTGTTA

TATA box AP1(+)

ACAGC***TATC***TGCTGGAAGGTTTTACTTCCTACAGACTCTGGAATCTGGGAAACAAAGACATCGAG ATG GCC GCT GCT GGG AAA GTAAGCTGTTTTTAA

GATA1(-) M A A A G K **

1

intron 1 (2559 bp) ATGTTTTCTTTACAG GTG ATT AAA TGC AAA GCT GCG GTG GCC TGG GCA GCT AAG CAA CCC TTC AGC ATT

** V I K C K A A V A W A A K Q P F S I

10 20

GAG GAC ATT GAA GTT GCT CCT CCA AAG GCT CAT GAA GTT CGC GTA AAG GTGAATATTGCCCAT intron 2 (508 bp) TATGCTTT

E D I E V A P P K A H E V R V K **

30 40

TGTGCAG ATG GTG GCA ACT GGG ATT TGC CGA TCA GAT GAC CAT GTA CTT AAT GGA TCA ATG AGT TTT CCG AAC TTT CCT GTG

** M V A T G I C R S D D H V L N G S M S F P N F P V

50 60

ATT CTG GGC CAT GAA GGT GCT GGT ATA GTG GAA AGC ATT GGC CCA GGA GTG AAA AAT ATA AAA CCA G GTGAAGCAAATGGGC

I L G H E G A G I V E S I G P G V K N I K P **

70 80

intron 3 (2849 bp) CCTTTCCCACTATAG GA GAC AAA GTC ATC ACT CTC TTC AAT CCC CAG TGC AGA GAA TGC AAA AAC TGT

** G D K V I T L F N P Q C R E   C K N C

90 100

TTA GAT CCC AAG AGC AAC GTG TGC ATT AAA TCT GA GTGAGTGACATTTTT intron 4 (1026 bp) ATACTTTTCATACAG C ATT GGT

L D P K S N V C I K S D ** ** I G

110

AAA TTA ACT GGA TTG ATG TTG GAC AAC ACC AGC AGA TTT ACA TGC AAG GGG AAA CAG ATC CAC CAC TTT GTG CAT ACC AGC

K L T G L M L D N T S R F T C K G K Q I H H F V H T S

120 130 140

ACC TTT ACT GAA TAC ACT GTG TTG GAT GAA ATG GCA GTT GCT AAG ATA CAC AAT GAT GCT CCT CTG GAT AAA GTC TGT TTA

T F T E Y T V L D E M A V A K I H N D A P L D K V C L

150 160 170

ATT GCC TGT GGG TTT TCT ACT GGC TAT GGC TCT GCT CTG AAC ACA GCC AAG GTAAGTGGTCTGGAA intron 5 (>8191 bp)

I A C G F S T G Y G S A L N T A K **

180 190

TTCTTTCCCCAATAG GTT GAA CCA GGA TCC ACA TGT GCT GTG TTT GGC CTG GGA GGT GTT GGT CTC TCT GTG ATT ATT GGA TGT

** V E P G S T C A V F G L G G V G L S V I I G C

200 210

AAA GTA GCT GGT GCT GCT AAG ATC ATT GGA GTG GAT ACA AAC GGT GAT AAG TTT GCC AAA GCT AAA GAA GTG GGA GCT ACT

K V A G A A K I I G V D T N G D K F A K A K E V G A T

220 230 240

GAG TGC ATC AAT CCA AAT GAT TAT AAG GAA CCT ATC CAC AAA GTG TTG GAA AAG ATG ACT GAT GGG GGC CTA GAT TAT TCT

E C I N P N D Y K E P I H K V L E K M T D G G L D Y S

250 260

TTT GAG TGT ATT GGA AAC ACC AGA GTC ATG GTTAGTAAACATTGT intron 6 (1485 bp) GGTTATATTTCTTAG GCA TCT GCT CTA

F E C I G N T R V M ** ** A S A L

270 280

CTA TCC ACC AAA TTT GCA TGC GGA ACA TCA GTT ATT GTT GGT GTA GCT CCT TCA ACA GCA GAA CTG AAT TTG GAT CCT ATG

L S T K F A C G T S V I V G V A P S T A E L N L D P M

290 300

GTG ATA CTC ACA GGA CGC ACT TTA AAA GGA TCT CTG TTT GGA G GTAATATATCTTTGC intron 7 (1214 bp) ACTAATATACT

V I L T G R T L K G S L F G **

310 320

ACAG GA TGG AAG AGC AAA GAT TGT GTT CCA AAA CTG GTT GCT GAT TTT ATG GAA AAC AAG TTT GAA CTG GAT GGG CTG ATA

** G W K S K D C V P K L   V A D F M E N K F E L D G L I

330 340

TCT CAC AAG CTA CCA CTT CAA AAA ATC AAT GAA GGA TTT GAT CTC TTG CAC AAA GGA ACA AG GTAGGTTATTAGCAG intron 8

S H K L P L Q K I N E G F D L L H K G T S **

350 360

(1276 bp) CTTTTTTAATCGTAG C CTT CGC ACC GTT TTG TAT TTC TGA TCTTCCGTGGAACGCTTCAAACGCTTATGCAAAGGAATCAACAGCTT

** L R T V L Y F stop

370

ACCAGCAGCACGAAGGCTTATTGAGTATACCATGAAAATAGTACTGAACTGTGATTTTCATTTACCAATGGAAAA**AATAAA**CACACTTTTGCACTGTTTATAATTGTGCTCAGATTAATACTCCTGCTAATAAGTATTTTTATGTGCTTACAAACGTGAAAACCTCTAAAATCACAAATTAAACATAGATTGTTAAAATGTGCCTAGGACAGCTCCCATTGGCTTCTACATGACCTTGCCAGCTTTTACATAGAATGCTTTGTATTAGCGTTTTTAGTGGTTTTTAAAGAAGAAGGAAAGGCTAATAAAGAGTTAATCTCAAGCTGCAGGCATACCTTCAGTTCTCTCAATAGTGCCCTTAAGTCTCCCCATATTTCACCTTTTCAGATGATCAGAAGCCAAACAGGAAGAAAAAATGCTGAGCTGTGTAAAGAAAGTTCCCATAATGCCTCACTCCTGCACCAAAAGCAAGACCAGTGTACATGCTCGGTTTGTAAGACTTCCTGCTGATTGGCTCAGATCCACATTCCTAAGGGGGGGGAGTGAGTTCTTAGCATTCTTGAGGGAGGGGGGAGCAGGAGAGGGGAGAGAGGAGAGAGCTGT
