# Supplementary material for: The Xenopus alcohol dehydrogenase gene family: characterization and comparative analysis incorporating amphibian and reptilian genomes
Source: BMC Genomics. 2014 Mar 20;15:216. doi: 10.1186/1471-2164-15-216 (PMC4028059; doi:10.1186/1471-2164-15-216)
Supplement: Additional file 4 — Xenopus tropicalis ADH1B cDNA sequence. The sequence includes the translated coding exons, intron flanking regions (±15 bp with total intron size), and the proximal promoter (-600 bp from the ATG codon) and 3′-untranslated region (650 bp) with predicted regulatory elements. Putative TATA boxes and polyadenylation signals are in bold and underlined. Putative transcription factor binding sites are underlined, with the core sequence of the matrix in bold and italics (for overlapping sites, the most downstream site is overlined); and the orientation (+ or - strand) is given in parentheses. [file 1471-2164-15-216-S4.doc]

***X. tropicalis ADH1B***

**-600**

CCCCCAGTGATTGTAACCACTTACCTGAAACCCCAGGCTGGTGCTCCTGTTAGCATAAAAGTGCACCAGCCCTGGGTATTTCTGCAGAGGATGAGGATCTTCTACTG

AAATACCCTTTGCTGGTGCAGTTTTTAGAGAAGAGGAGCACCAGCACAGGGTATCAGGTAAGCGATTATAATCACTGGGGGGGGGGGTATGCCTACTGTTGCACCCC

CCTGTGAATTTAAATTCAGCTCTTCTAAGGTTAATTTTAAGCTGAAGAATGTGCTTAAGCTGTGC***TGACC***TGTTGCACTGATCAAATTTCTATTAAATACATGGATC

ER(+) RORA1(-)

ACTG***TTTAT***AGCAGTGAATAGA***GGTC***ATTCCTTAGTGCATGAGATTCTGGCTG***GTTAC***ATAACACTGTACATAATATATACAATACATACATACTGTATCCAGTAAG

XFD2(-)HFH1(+) RORA1(+) ER(-) HLF(+)

CAGTTGCTGCTCATAAATAATCCATTGCAGATGTAAGTTCACAGTGAGAATTTTCTAGGTGGAGTTAACATAATATA***GGGCGG***CCCTGGTAGGTGGTTACATT**TATA**

SP1(+) TATA box

ACAGCTGT***AGATA***GAGTGTAACGGTGAGGCAAGCAAGCTGTAAACTGGGAAGCAGAGACATCGAG ATG GCA ACT GCT GGG AAA GTAAGCAGCAAAGTC

GATA1(+) M A T A G K **

1

intron 1 (2897 bp) TTTTTCTTTTTACAG GTG ATT AAA TGC AAG GCA GCT GTG GCC TGG GGG CCC AAG CAA CCC CTC ACC ATC

** V I K C K A A V A W G P K Q P L T I

10 20

GAG GAT ATT GAA GTT GCT CCA CCA AAG GCT CAT GAA GTT CGT GTA AAG GTA AAGATTACAAAT intron 2 (1932 bp) CCATT

E D I E V A P P K A H E V R V K **

30 40

AC TATTGCAG ATT GTG GCA ACT GGC ATT TGT CGT TCA GAT GAC CAT GTT CTT AGT GGA GCT ATA AGC GAC ATG AAG TTC CCA

** I V A T G I C R S D D H V L S G A I S D M K F P

50 60

GCG ATT CTT GGG CAT GAA GGG GCT GGC ATA GTG GAG AGT GTG GGA GAG GGA GTG AAA AAT ATA AAA CCA G GTGAGACTTATTG

A I L G H E G A G I V E S V G E G V K N I K P **

70 80

TA intron3 (3516 bp) GTTTTGTGTTTCTAG GA GAC AAA GTT ATC CCA CTC TTT GTT CCC CAA TGT GGA GAA TGC AGA TGC TGC

** G D K V I P L F V P Q C G E C R C C

90 100

ACT AAT GTG AGG AGC AAC CTA TGT GAC AAA CAT GA GTATGTGATTTACTG intron 4 (555 bp) ATTTGTCTTATACAG T ATT GGA

T N V R S N L C D K H D ** ** I G

110

CCA TAC AGA GGA CTC ATG TTG GAC AAC ACC AAT AGA TTT ACA TGC AAG GGG AAT CCG GTT TAC ACT TTT TTA AGC ACC AGC

P Y R G L M L D N T N R F T C K G N P V Y T F L S T S

120 130 140

ACC TTT ACT GAA TAC ACT GTC TTG GAT GAG ATA TGC GTG GCT AAG ATT GAT GAC AAT GCT CCT CTT GAT AAA GTG TGT TTA

T F T E Y T V L D E I C V A K I D D N A P L D K V C L

150 160 170

ATC GGC TGT GGC TTT TCC ACT GGT TAC GGC TCT GCT GTG AAG ATT GCT AAG GTGAGGGCTTTGTTG intron 5 (924 bp) TCTT

I G C G F S T G Y G S A V K I A K **

180 190

TTATCCTACAG GTT GAG CAG GGG TCT TCA TGC GCT GTG TTT GGC CTG GGT GGT GTT GGT CTT TCT GTG CTT ATT GGA TGT AAA

** V E Q G S S C A V F G L G G V G L S V L I G C K

200 210

GTA GCT GGT GCT TCT AAG ATC ATT GGA GTG GAT ACA AAC AGT GAC AAG TTT GCC AAA GCA AAG GAA TTA GGG GCC ACT GAA

V A G A S K I I G V D T N S D K F A K A K E L G A T E

220 230 240

TGC ATT AAC CCA AAC GAT TAT AAT GAA CCT ATC CAT GAA GTG CTG GCA AAG ATA TCT GAT GGA GGA TTA GAC TAT ACC TTT

C I N P N D Y N E P I H E V L A K I S D G G L D Y T F

250 260

GAG TGC ATT GGA AAC ACC AAG GTC ATG GTAAGAAACAATACT intron 6 (1198 bp) GTGCATGCTTTCCAG GAA TCT GCT CTT AAG

E C I G N T K V M ** ** E S A L K

270 280

GCT ACC CAC TTT GGA TGT GGC ACA TCA GTC ATC GTT GGT TTA GCT CCT GCC TCA GCC AGG GTA TCA GTG GAC CCA ATG GAG

A T H F G C G T S V I V G L A P A S A R V S V D P M E

290 300

ATG CTC ACA GGA CGC ACA TTA AAA GGA GCT TTG TTT GGA G GTGAGGGATTGAAAT intron 7 (1712 bp) CTGCCAATATTTCAG GC

M L T G R T L K G A L F G ** ** G

310 320

TGG AAG AGC AGA GAC GAA GTC CCT CAA CTG GTT GCT GAC TTT TTG GCA AAG AAG TTT GAA CTT GAT GGG CTG ATA ACT CAT

W K S R D E V P Q L V A D F L A K K F E L D G L I T H

330 340 350

AGG TCA ACT CTT GAT AAA ATC AAT GAA GGG TTT GAT CTC CTG CGC AAA GGG GAC AG GTAGGTTATTAACAG intron 8

R S T L D K I N E G F D L L R K G D S **

360

(1282 bp) TATCTCTTCCCTCAG C ATT CGA ACC ATC CTT CAG ATC TCA CAG TGA GTTTGCTGCATTGCCCCATCAGATCGTGGTCATACAGCT

** I R T I L Q I S Q stop

370

TCAGTGGAATCACTATGTGGATCATAAAGATTTAAAGCTAAAAATACATAGCAATATATAGCAAAGCCAATTTAATGCATTTTATAGCTTCTTTTTCTATATAGAAACAAACTGTAACTGTGACTTAAAGAC**AATAAA**TATATCTGCTTAAATGATCCTGGTCTCTTTTATATCCACCAATAGACCTGTGCAGGAACAGTGGCCAAATTATTTCCATACCAGAGTATTTTCAGTCACAGCAGGGTCGAACTAGGCCACCAGGACACTGCAAAAAAAACATGGTGGGCCCCGGCGGCCCAGACCCCAATCCCTGTTGCCTCTTCCCCTGGCTGCTGATGTCCTCCCCTGATGCGTTTTACTTATGCAGGCTCAGGGGAGGACATTGGGCAGGTGGCCCCTGCAGGGGGTTAGGGGGGGTTGTGACGTGGGGTGCGGGTGGCTCGGCTGGCAGGGGCACCTTGGGGAGTGCAGGGCCCACTGTGAGTCACAGTATTAATGGGGGAGTTACAAGCAGTGGTATTTTAATATAATTTGTAATCGGCCTTCATTTTTCTGCAGCTTTCCAACTTATAATATTAATGGCATTTTGACTGCATGGGCTTATATTCTCT
