# Supplementary material for: The Xenopus alcohol dehydrogenase gene family: characterization and comparative analysis incorporating amphibian and reptilian genomes
Source: BMC Genomics. 2014 Mar 20;15:216. doi: 10.1186/1471-2164-15-216 (PMC4028059; doi:10.1186/1471-2164-15-216)
Supplement: Additional file 5 — Xenopus tropicalis ADH1C cDNA sequence. The sequence includes the translated coding exons, intron flanking regions (±15 bp with total intron size), and the proximal promoter (-600 bp from the ATG codon) and 3′-untranslated region (650 bp) with predicted regulatory elements. Putative TATA boxes and polyadenylation signals are in bold and underlined. Putative transcription factor binding sites are underlined, with the core sequence of the matrix in bold and italics (for overlapping sites, the most downstream site is overlined); and the orientation (+ or - strand) is given in parentheses. [file 1471-2164-15-216-S5.doc]

***X. tropicalis ADH1C***

**-600**

CCTGAATACACCTGGAACAAA***TATCT***GTCATGCTACACACCCCTTCAAGAACTGGCTGTGAAATG***AAATA***AAAAAATCTGCTGTTAGTGAAAGATTTAAACTCCTAT

GATA1(-) HNF3B(-)

ATTCCAGACACTCCTGCACTAATATGAAT***GCAAA***GAAAATTGCCTCCAGAGCTTGCACTTCACTGCATC***TTATC***TCTGGCATGTCATGCCAGTGGTTTGATGCATTT

OCT1(+) GATA1(-)

TATACATAATATTACCCCTATGAAAATGGTGCAAGGAAATTTTGTTGACACTTGCAAAATCAGCACACTGCCTGCATTGTATGCCTCTTAAGAAACACTAAAAGTAA

GTAGACATACGTTTGTGTGTGAGCTTCAAGTGTGCAAAAGCCAACCCCACCCATAAAACATTATATAAGTGCAA***AGATAA***ATGTGCAGGGATCCCTTGTACTGCTGT

GATA1(+)

GCAGATTGTCGAACAGCCCATTTTATAACAGGCAGGCTAGAATGTAGCTGTGAGCTTCAAACTCCTATGTGGCATCACACCCTCTGTCTGTCCC***CCGCCC***TGGTATG

SP1(-)

GCACAG**TATA**ACTGGGCTAAACTGAGT***TTATCT***TGTACAGACTGCAGCCAGGAAGCACAGATATC ATG TCT ACA GCT GGG AAA GTAAGCAGTTAGCTT

TATA box GATA1(-) M S T A G K **

1

intron 1 (3208 bp) ATTGTTTCTTTACAG GTG ATT AAG TGC ACA GCA GCC GTG GTC TGG GAG CCT GGC CAA CCC TTC AGC ATC

** V I K C T A A V V W E P G Q P F S I

10 20

GAG GAC ATT GAA GTT GCT CCT CCA AAG GCT CAT GAA GTT CGA GTG AAG GTGAGAATTGCTCGC intron 2 (407 bp) TATTCTTTA

E D I E V A P P K A H E V R V K **

30 40

ATACAG ATT GTT GCA TCT GGA GTA TGC CAT ACA GAT TAC CAT GTC ATG AGT GGA TCA GTA GAT AAT ATC AAG TAC CCA TTG

** I V A S G V C H T D Y H V M S G S V D N I K Y P L

50 60

ATT CTG GGG CAT GAA GGT GCT GGT ATA GTG GAA AGT GTG GGA GAA GGA GTA AAA AAA GTA AAA CCA G GTAAGACTTGGTCAGCT

I L G H E G A G I V E S V G E G V K K V K P **

70 80

intron 3 (769 bp) TGTCTTTCTCCCTAG GA GAC AAA GTC ATT CCT CTG TCG CTT CCC CAG TGT GGA AAA TGC CTT TTA TGT

** G D K V I P L S L P Q C G K C L L C

90 100

TTA AAT CCC AAG AGC AAC ATA TGC GAA AAA TCT GA GTTAGTTTATTCTTT intron 4 (1766 bp) CCTAAATTTGAACAG T TTT GGA

L N P K S N I C E K S D ** ** F G

110

AAA TAT TCT GGA CTC ATG TTG GAC AAC ACC AGT AGA TTT ACA TGC AAG GGG AAG CTG ATT TAC CAC TTT GTA AGA ACC AGC

K Y S G L M L D N T S R F T C K G K L I Y H F V R T S

120 130 140

ACC TTT ACT GAA TAC ACT GTC TTG GAT GAG ATA TCC GTG GCT AAG ATT GAT GAC AGT GCT CCT CTC GAT AAA GTG TGT TTA

T F T E Y T V L D E I S V A K I D D S A P L D K V C L

150 160 170

ATC AGT TGT GGA TTT TCC ACT GGT TAC GGC TCT GCT GTG AAG ATT GCT AAG GTGAGGGCTTTGTGT intron 5 (> 3536 bp)

I S C G F S T G Y G S A V K I A K **

180 190

TTCTATACTCTGCAG GTT GAG CCG GGA TCC ACA TGT GCT GTG TTT GGC CTG GGT GGT GTT GGC CTG TCT GGA ATT ATT GGC TGT

** V E P G S T C A V F G L G G V G L S G I I G C

200 210

AAG GCA GCT GGT GCT TCT AGA ATA ATT GGA GTG GAT ACA AAC AGC AAA AAG TTT ACA GCA GCC AAA AAT GTA GGA GCC ACT

K A A G A S R I I G V D T N S K K F T A A K N V G A T

220 230 240

GAA TGC ATC AAT CCA AAT GAT TAT AAG GAA CCA GTC CAT GAA GTG CTG ACA AAG ATG ACT GGG CTG GGA ACA GAC TAC TCA

E C I N P N D Y K E P V H E V L T K M T G L G T D Y S

250 260

CTT GAG TTT GTG GGA GAT ACC AAT GTT ATG GTTAGTAAACACATC intron 6 (1114 bp) TTTTACATTTCCTAG TTG TCT GCT CTT

L E F V G D T N V M ** ** L S A L

270 280

CTG TCC ACC AAC TTT GCA TCT GGA ACA ACA GTT ATT GTT GGC TTA GCT CAT TAT ACA GCA AAG ATG AAC TTC AAT CCA ATG

L S T N F A S G T T V I V G L A H Y T A K M N F N P M

290 300

GTG CTG CTC ACA GGA CGC ACT TTG AAA GGA GGC TTA CTT GGA G GTAAATTGCAGTACT intron 7 (1387 bp) ACTACTCTATT

V L L T G R T L K G G L L G **

310 320

ACAG GA TGT AAA AGC AAA GAT ATC CCA AAA CTG GTT TCT GAT TTA ATG GCA AAG AAA TTT GAC CTT GAC GGA CTG ATA ACT

** G C K S K D I P K L V S D L M A K K F D L D G L I T

330 340

CAC AAG ATG CCA CTT GCA AAA ATC AAT GAA GCC TAT GAT GTC CTG ACC AAA GGA GAA AG GTAGGTTATTGCTAG intron 8

H K M P L A K I N E A Y D V L T K G E S **

350 360

(1671 bp) TGCTTTCGTTTACAG C CTT CGT ACT ATT TTG ATG ATG AGT TCG TGT GAA TGA TGATGATGAGTTCGTCTGTTGAAATCAAC

** L R T I L M M S S C E stop

370

TCAGAAACAACCTGGATTATATACTATAAATACAGATAATATTAGTCCAGTTTCATTTGCTAATGGCTAAATAGAGAAAAAATCATCATCATTGCAGAACTTGAGCTCCATCTTCCTCCAGGCTGAACGGTGACCCCATACAAGTCTTATACTGCAAGGTCAGCATTTGCATTTCATAGAAGACTCTGTATGTGAGTGTCACTATAGAAAACTGCAATTTACTCTTCTAACAGCTGGTGTATCTGTAATCTGCAAAACAAAAACAAGGGCATAGAGTTTGCTGGTCTTTGGTCATTACTGTTCAGAGCTGCTGGCATATTTGGGTCATTGGTTGCCTATGTGTCTGCTTT**AATAAA**GGATGGATACAAAATTTATTGTTGCTTTGTGTTTCTAAATTTCTGCAGAGCACAATCTGCATTTTTTGTGTTTTTTTACAAAAACAAAAATACTCATCTTTATGACTTGAACATGCAGCTCCACTTTCTAAAGCTCTATAAATGAGTTGGGTCTGCAG
