# Supplementary material for: The Xenopus alcohol dehydrogenase gene family: characterization and comparative analysis incorporating amphibian and reptilian genomes
Source: BMC Genomics. 2014 Mar 20;15:216. doi: 10.1186/1471-2164-15-216 (PMC4028059; doi:10.1186/1471-2164-15-216)
Supplement: Additional file 6 — Xenopus tropicalis ADH2 cDNA sequence. The sequence includes the translated coding exons, intron flanking regions (±15 bp with total intron size), and the proximal promoter (-600 bp from the ATG codon) and 3′-untranslated region (650 bp) with predicted regulatory elements. Putative TATA boxes and polyadenylation signals are in bold and underlined. Putative transcription factor binding sites are underlined, with the core sequence of the matrix in bold and italics (for overlapping sites, the most downstream site is overlined); and the orientation (+ or - strand) is given in parentheses. [file 1471-2164-15-216-S6.doc]

***X. tropicalis ADH2***

**-600**

CT

GCAGCC***TATCT***ACTGACAAGGCTACCAACTTTCCCTTGCCTTTATTGAAAGGTTAAATGGCTTATGTTGTGACCCCCCCACTACCTCCTTGTGTGCCTTGGGCCCTG

GATA1(-)

AATGTATAGGTAGTTGGGAAGTG***GCCAA***GGAACTTAAAAGCTTTATACATAATATATTGGATAACTTGTGTCTTGATATTTTGATATTATACGTTACTT***TGATT***TGG

NF1(-) GFI1(-)

TTTTGGGCTGCAAAGTGTTTTTTCCATTCTAGCACTCCATGTCATCCC***TTGGC***ATAAATATAGTTC***AGTCA***TGGTATCTAGGTTTTTGAT***GCCAA***CGTGCAT***GATAA***

NF1(+) AP1(-) NF1(-) GATA1(+)

AAGATCTCTAACAGTGGTCACAAGGTTTTGTACAGTAAATCCACTCATGGAACTATGTTTCTATTGGGCATAACAATCCCTGTTATGCTTGTCTCTTACAGAATTAC

CACTGGGTTACAAGTAACAAAGGAGCTGCAAGTAGAAGAGAGGAAAGGGCGTGA**TATA**TTTGGGACTGGT**TATA**GAGCAGTG**TATT**AAACCCATATGAGGCAGTTGT

TATA box? TATA box? TATA box?

GTTTGTGTGCACTAGGAGAGAGCAGCCTGCACATCTCCAAACACCTCTAAGAGAGA***AGTCA***CC ATG AGC ACA GCC AGG AAA GTAAGGAAAACCTATTC

AP1(-) M S T A R K **

1

intron 1 (3615 bp) ATGTTTATTTTGCAG GTT ATA AAG TGC AAG GCA GCT GTA GCC TGG GAA GCT GGC AAG CCT TTT TCT ATA

** V I K C K A A V A W E A G K P F S I

10 20

GAG GAA ATT GAA GTT GCT CCT CCT AGG GAT CAT GAA GTC CGT GTA AAG GTAACAATAAAGCCT intron 2 (1444 bp) TTCTTTCC

E E I E V A P P R D H E V R V K **

30 40

TCTATAG ATT GTT GCC ACA GGA GTC TGC CGA ACT GAT GCA CAT GCT GTG AGC ACT CAT TTC AAA GAA GGG CTG TTC CCA GTG

** I V A T G V C R T D A H A V S T H F K E G L F P V

50 60

ATT CTT GGC CAT GAA GGT GCT GGT ATT GTG GAA AGT GTT GGC CCA GGT GTC ACC AGA GTG AAA CCA G GTATAAAACCTAAAT

I L G H E G A G I V E S V G P G V T R V K P **

70 80

intron 3 (453 bp) TTTTTTGCTTTCCAG GC GAT AAA GTT ATT CCT CTT TAT ATT CCA CAA TGC GGT GAA TGC AAG TTC TGT

** G D K V I P L Y I P Q C G E C K F C

90 100

CTG AAT CCC AAA ACC AAC TTG TGT GAA AAG ATA AG GTAAAAATGCTAAAT intron 4 (438 bp) ATTTCATATCCTTAG T AAA ATT

L N P K T N L C E K I S ** ** K I

110

AAG ACT GCC ATT TCG GAT CAA GAC CTG ATG GCA GAC AAC ACA AGC AGG TTT ACC TGC AAG GGG AAG CAG ATA TAT CAT TTT

K T A I S D Q D L M A D N T S R F T C K G K Q I Y H F

120 130 140

ATG GGC ACC AGC ACC TTT TCA GAA TAC ACT GTG TGT GCT GAA ACC TCA GTT GCT AAA ATT GAT GAT GCG GCT CCT CTG GAG

M G T S T F S E Y T V C A E T S V A K I D D A A P L E

150 160 170

AAA GTC TGC CTG ATC GGT TGT GGT TTC TCC ACT GGT TAT GGT GCT GCC ATC AAC ACT GCT AAG GTAAAACAATGATGA intron

K V C L I G C G F S T G Y G A A I N T A K **

180 190

5(3211 bp) TTTTTTTATGTTCAG GTT GAG CCT GGC TCC TCC TGT GCA GTT TTT GGC TTA GGA GGT GTT GGG CTC TCA GCT GTC

** V E P G S S C A V F G L G G V G L S A V

200 210

ATG GGA TGT AAA GCT GCA GGT GCA AGT CGA ATT ATT GGC ATT GAC ATC AAC AGT GAT AAA TTC GAG AAA GCT CTA GAG CTG

M G C K A A G A S R I I G I D I N S D K F E K A L E L

220 230 240

GGA GCC ACC GAA TGC ATC AAC CCT AAA GAT TAT GAC AAG CCA ATT CAA CAG GTC ATA TCG GAA ATG ACT GGT GGT GGC GTT

G A T E C I N P K D Y D K P I Q Q V I S E M T G G G V

250 260

GAT TTC TCC ATT GAA TGC ATT GGA ATT ATT GAT GTG ATG GTTAGTATATGCACT intron 6 (625 bp) ATCATTTCTTTGTAG AAA

D F S I E C I G I I D V M ** ** K

270 280

GCT GCA CTG GAG TGC ACC ACA GTG GGC TGG GGA ACA TGT GCA ATA GTG GGA GTC TCA TTG GAT GAA CAT GGA TTG CCT GTT

A A L E C T T V G W G T C A I V G V S L D E H G L P V

290 300

GCT CCT TTC CAT TTG CTT ATG GGA AGA ACT TTA AAG GCA ACT TTC TTT GGA G GTATGTCAGAGTTGG intron 7 (1274 bp)

A P F H L L M G R T L K A T F F G **

310 320

TTATTTGTTTTATAG GC TGG AAA AGT GTG GAC AAT GTT CCA AAG CTG GTG GAA GAT TAT TTG GGG AAT AAA TTT GAT CTG GAT

** G W K S V D N V P K L V E D Y L G N K F D L D

330 340

TCT TTA GTA ACA TTT ACT CTG CCA TTT GAT AAA ATA AAT GAA GCT TTT GAT TTA ATG CGT GAT GGG AAA AG GTGGGTATTTA

S L V T F T L P F D K I N E A F D L M R D G K S **

350 360 370

AAAG intron 8 (1825 bp) CTTTTTCTCTTCCAG C ATC CGA ACA GTA TTG GTA TTC TAA ATCGTGGTTCTGAACTTATTCAAGGCAAAAA

** ** I R T V L V F stop

380

GTCCTGTCAATTTAATTGGAATTTTTCGAAAATATACTAGTAGATGTGTTCTAAAATATCATGTATGTTCATTTATTTGTGTTTTTTTAAGTATTTTGAACTGGCTTTACAGAAATAGGCAAATGTATAAGTGTGTATCTGATGTAAACTTAGATGTAACAACCTGATATAAAACTGTGTGTATCTAA**AATAAA**TCTGTGAACTGTTTTGCAAAATAAGTCCCTGTACATAATGATTTATCTGCACCATTCAACCATATAT**AATAAA**TAGTGCATGAGAATATATCTGTCTTCTTTTAAGATTATTAGAATTCCAAAAAAATCCAAATGTCTTATTATTAGAAACAAAAGTACAATTAGTACAAACATTTTTATGTACTGTAGTAAAAATTGCAGTAAATTTCTACTTGATCTTTATGAATACAAGATTTAGATTCAGACAAATAGTAGTGCCGGGTCAAATATGATCCTTTTATAACCAAACCAATAATAATATTGCATGACGTTTAATCA
