# Supplementary material for: The Xenopus alcohol dehydrogenase gene family: characterization and comparative analysis incorporating amphibian and reptilian genomes
Source: BMC Genomics. 2014 Mar 20;15:216. doi: 10.1186/1471-2164-15-216 (PMC4028059; doi:10.1186/1471-2164-15-216)
Supplement: Additional file 7 — Xenopus tropicalis ADH3 cDNA sequence. The sequence includes the translated coding exons, intron flanking regions (±15 bp with total intron size), and the proximal promoter (-600 bp from the ATG codon) and 3′-untranslated region (650 bp) with predicted regulatory elements. Putative TATA boxes and polyadenylation signals are in bold and underlined. Putative transcription factor binding sites are underlined, with the core sequence of the matrix in bold and italics (for overlapping sites, the most downstream site is overlined); and the orientation (+ or - strand) is given in parentheses. [file 1471-2164-15-216-S7.doc]

***X. tropicalis ADH3***

**-600**

AATTGCATATAACTAAAAACAACTCTGTTCTACAAATGAAGAGTTGTTATTAACGATTCTTAAATC***TATCT***AA***TATGC***ACCATGCTAGGAAAATCTCCTGTACTCTC

GATA1(-) OCT1(+)

TAGAACAGGGGTCCCCAACCTTTCTCACT***CGTGA***GCCAC***AGTCA***ATCGTAAAAACACTTGGAGAGCAACACAAGCACCATAAAAGTTCATGGAGGAGCCAAATAAGG

USF(-) AP1(-)

GCTAAG***ATTGG***CTATTAGGCTGCCTCTATGCACAC***TATCT***GCTTTCAGGGGCTTTATTTGGCAGTAAATCT***TGTTT***TTATTCAACCAAAACTTGCCCCCA***AGTCA***GG

CCAAT box(-) GATA1(-) HFH3(+) AP1(-)

AATTAAAAAATAACTTTCTGGTTTGGGGGCACTGAGAGCAACATCCAAGGGGTTGGGGAGCAACATGTTGCTCACAAGCC***ATTGG***TTGGGGATCACTGCTCTAGAAG

CCAAT box(-)

CT***GTTGG***CTTGTGCACAAAACCCATGTAGTCCGCTCTGGGTCCTTCATGCCACAAGATGGCGCTGGCGATCCCAGGCAAGCCTTACTGTTCCGCACCGAAGCTGGAA

CMYB(+)

GGCGCCTGGAACCCATAGGAGGGAGGCGTGGCTCCACACACACAGTCTTGTGCACAACGTGGATT ATG GAG ACA GCG GGG AAG GTGAGAAGCATCCTG

M E T A G K **

1

intron 1 (1731 bp) TGTATCGCCTTTTAG GTC GTC AAA TGT AAA GCT GCT GTA GCA TGG GAG GCA GGA AAA CCC CTC TCA ATT

** V V K C K A A V A W E A G K P L S I

10 20

GAA GAA GTT GAA GTG GCT CCC CCA AAA GCT CAT GAA GTT CGC ATT AAG GTAAAATAACTAGAT intron 2 (416 bp) ATTTTAAT

E E V E V A P P K A H E V R I K **

30 40

ATTGCAG ATA GTC TCC ACT GCT GTG TGC CAT ACA GAT GCC TAC ACA TTG AGT GGT GCT GAT CCA GAA GGA TGT TTC CCT GTA

** I V S T A V C H T D A Y T L S G A D P E G C F P V

50 60

ATA TTG GGT CAT GAA GGA GCT GGC ATT GTG GAA AGT GTA GGA GAA GGT GTT ACC AGA GTA AAA CCA G GTAAGGATTTTCATG

I L G H E G A G I V E S V G E G V T R V K P **

70 80

intron 3 (604 bp) TGTTTCATTTCACAG GT GAC AAA GTA ATA CCC TTG TAT ATC CCA CAG TGT GGT GAA TGC AAG TTC TGT

** G D K V I P L Y I P Q C G E C K F C

90 100

TTG AAT CCC AAA ACA AAC CTG TGC CAG AAG ATA AG GTATTACAAGCAGCC intron 4 (543 bp) ATCATTCTTTGGCAG G ATT ACT

L N P K T N L C Q K I R ** ** I T

110

CAA GGC AAG GGG TTT ATG CCT GAT GGC AGC AGT AGG TTC ACT TGC AAA GGA CAA CAG ATT TTC CAC TTC ATG GGC ACC AGC

Q G K G F M P D G S S R F T C K G Q Q I F H F M G T S

120 130 140

ACT TTC TCT GAA TAC ACT GTT GTA GCA GAT ATC TCC GTT GCT AAA ATA GAT GAC TCT GCT CCT CTG GAC AAA GTC TGC TTG

T F S E Y T V V A D I S V A K I D D S A P L D K V C L

150 160 170

CTG GGC TGT GGA ATC TCA ACT GGT TAT GGA GCT GTC ATT AAC ACA GCA AAG GTAACTGATTTCTAG intron 5 (600 bp) TTTT

L G C G I S T G Y G A V I N T A K **

180 190

GTGTCCATCAG GTT GAA CCT GGC TCT ACA TGT GCT GTC TTT GGC TTA GGA GGG GTT GGT CTT GCA GTC ATT ATG GGC TGT AAA

** V E P G S T C A V F G L G G V G L A V I M G C K

200 210

GTA GCC GGA GCT ACT CGC ATT ATT GGC ATT GAC CTT AAC AAG GAC AAG TTT GTA AAG GCA ACA GAG TTT GGA GCT ACA GAT

V A G A T R I I G I D L N K D K F V K A T E F G A T D

220 230 240

TGT TTA AAC CCA GCA GAC TTC AAA AAA CCT ATT CAG GAT GTG CTG ATT GAA ATG ACT GAT GGA GGA GTC GAC TAT TCC TTT

C L N P A D F K K P I Q D V L I E M T D G G V D Y S F

250 260

GAG TGT ATT GGC AAT GTC GGT GTT ATG GTAATTATTTATTTA intron 6 (363 bp) TGTTCTTGTGGTTAG AGA GCT GCC CTG GAA

E C I G N V G V M ** ** R A A L E

270 280

GCG TGT CAC AAA GGC TGG GGT ACA AGT GTT ATA GTT GGT GTG GCA GCG TCT GGC CAG GAG ATT GCT ACA CGC CCA TTT CAA

A C H K G W G T S V I V G V A A S G Q E I A T R P F Q

290 300

CTT GTC ACA GGG AGG GTT TGG AAA GGA ACT GCA TTT GGA G GTGAGAACATGTCCA intron 7 (674 bp) TTTTGTGTCTTGCAG

L V T G R V W K G T A F G ** **

310 320

GA TGG AAG AGT GTG GAC AGT GTG CCA AAG CTG GTT TCT GAA TAT ATG GCA AAA AAG ATT AAG GTT GAT GAG TTT GTG ACT

G W K S V D S V P K L V S E Y M A K K I K V D E F V T

330 340

CAC ACT TTA CCC TTT AAT TCT ATC AAT GAA GCA TTT GAA CTC ATG CAT GCA GGG AAG AG GTAAGAGCTCTTAAA intron 8

H T L P F N S I N E A F E L M H A G K S **

350 360

(1875 bp) TTCCCTTTTTTGCAG C ATT CGT GGT GTT TTG AAT TAT TAA CAGTCTTTCTGAAGAAATCCTTCACAAGAAACATGATGGATCTCAT

** I R G V L N Y stop

370

CTCCGTAGATTAATACAAAGCACTTATTACTTAATTAGATACAATCCCTGCCAGTGCATTTGTTACAGAACTTGATTGACATAAATTTACTAAAAATTCTCTTACTGATTTGACAGACTGTAGCTAAATAGCT**AATAAA**ATTAAGTAACATTGTTTACTTGTTCAATTTAGTTGCTCAGTGTGTTTCTTTTGGTTTGGATTTGTTTTGATGCACCTAAAAATGTAATCAGCCCAACATCTTATGGGGGGATATTGCAAAGACTTAAGGTGGCCAAACACAGGCAGATTTAAAATACAATGTATATCAGTTGGGCACGACTCCCCCGCCCAAATGGAATTATTTGTACTGCATATATCCCCTCCGCTTGCCAGCACCAGCACATTTTCCTAAAGGAAATAGCAGCTTTCACCCGGTGGCCATTTTTCCTCTGATACATAATCAGATACATTTAAATCTGCAAACAGCATACACACACACAGGCCCTTATTCAGCATAC
