# Supplementary material for: The Xenopus alcohol dehydrogenase gene family: characterization and comparative analysis incorporating amphibian and reptilian genomes
Source: BMC Genomics. 2014 Mar 20;15:216. doi: 10.1186/1471-2164-15-216 (PMC4028059; doi:10.1186/1471-2164-15-216)
Supplement: Additional file 8 — Xenopus tropicalis ADH7 cDNA sequence. The sequence includes the translated coding exons, intron flanking regions (±15 bp with total intron size), and 3′-untranslated region (650 bp) with predicted regulatory elements. Putative TATA boxes and polyadenylation signals are in bold and underlined. [file 1471-2164-15-216-S8.doc]

***X. tropicalis ADH7***

**-76**

ATGGCGGAACCAGGAAGTGAATGCGCGACACAGTACAGCAGGGCACTGGCTGGGGGAATATACCAGGGGAGGAACT ATG GAC ACT GCT GGG AAA

M D T A G K

1

intron 1 (> 1920 bp) TTTTCTAACTAATAG ATT GTA AAA TGC AAA GCG GCT GTA GCC TGG GAG ATT GGA AAA CCC CTT ACA

** I V K C K A A V A W E I G K P L T

10 20

ATT GAA GAA ATA GAG GTT GAA GTA CCA AAA GCC AGT GAA GTT CGA ATC AAG GTATGAGACCAATAA intron 2 (> 1976 bp)

I E E I E V E V P K A S E V R I K **

30 40

TTGTCAATTTTTTAG ATG GTT GCA ACA GGA ATC TGT CGC ACC GAC GAC CAT GTT TTG AAG GGG GCT TTA AAA GGT ATT GAT TTT

** M V A T G I C R T D D H V L K G A L K G I D F

50 60

CCT GTC ATT TTG GGC CAT GAA GGA GCT GGA ATA ATT GAA AGC GTT GGA GAA GGT GTG ACT GGA CTT AAA CCA G GTATAGTTT

P V I L G H E G A G I I E S V G E G V T G L K P **

70 80

GCAGAA intron 3 (356 bp) TTTCTGTTTTTGCAG GA GAC AAA GTT ATT CCA CTT TGT ATC CCA CAG TGT GGA AAG TGC AGT

** G D K V I P L C I P Q C G K C S

90 100

TCT TGC CTT AAT CCA AAT ACC AAC TGC TGC CTT AAA ACT CA GTAAGTGTTTTAATA intron 4 (> 3620) CAAATATATTTACAG T

S C L N P N T N C C L K T H ** **

110

CTC AGT GAG TCA CAA AAT GTA ATG CCT GAC AAG ACA TCT AGA TTT TTG TGC AAA GGA AAA GCT GCT TAT CAC TTT TTA TGG

L S E S Q N V M P D K T S R F L C K G K A A Y H F L W

120 130 140

ACA AGC ACC TTC TCG GAA TAT ACT GTG GTT CCA GTT GAT GCA GTT GCA AAA ATT GAT GAC AGA GTA CCC ATG GAT AAG GCC

T S T F S E Y T V V P V D A V A K I D D R V P M D K A

150 160 170

TGC CTT TTT GGA TGT GGT TTT CCA ACA GGA TAT GGA GCT GTT GTC AAT ACT GCT AAG GTACTACATGTAAAG intron 5

C L F G C G F P T G Y G A V V N T A K **

180 190

(376 bp) ATTTTCTTTCTGTAG GTG GAG CCC GGT TCT ACT TGT GCT GTG TTT GGG TTA GGT GGA ATT GGC CTT TCA GCT GTA

** V E P G S T C A V F G L G G I G L S A V

200 210

ATA GGT TGC AAA TCT GCA GGA GCT GCA ATA ATT ATA GCT GTT GAT ATA AAC AGT GCT AAA TTC GAT ATC GCT AAG GTG TTT

I G C K S A G A A I I I A V D I N S A K F D I A K V F

220 230

GGG GCA ACA GAA TGT ATC AAT CCT CTG GAT TAT TCC AAG CCA ATC CAG GAG GTG ATT ACT GAA ATG ACA AAT GGA GGA GTA

G A T E C I N P L D Y S K P I Q E V I T E M T N G G V

240 250 260

CAT TAT TCC TTT GAA TGT ATT GGA AAC ACA GAT ACA ATG GTAAGCATTCTACTG intron 6 (1651 bp) ACTTGTTATTAACAG AAA

H Y S F E C I G N T D T M ** ** K

270

GCG GCC CTG GAA TGC TGT CAC ATG GGA TAT GGA ACC AGT GTT ATT ATT GGA GAG GCT CCA TCA GCA GCA CAA ATC TCT TTT

A A L E C C H M G Y G T S V I I G E A P S A A Q I S F

280 290 300

GAT CCA ATA TTG CTG TTT ACA GGA CGT ACA TGG AAA GGG AGC ATA TTT GGA G GTACAGTTAAAAAAT intron 7 (1292 bp)

D P I L L F T G R T W K G S I F G **

310 320

TTGTTGTTCATTTAG GT TGG AAA AGC AAG GAA AGT GTC CCT CGA CTG GTT GAT GAA TTC ATG GCA AAT AAA TTT AAT CTT GAT

** G W K S K E S V P R L V D E F M A N K F N L D

330 340

GGA TTG GTG AGC CAC ACT TTG CCT TTT GAT CAG ATA AAT GAG GGG TTT GAG CTT TTA CGC TCT GGT AAC AG GTGGGTCATTTT

G L V S H T L P F D Q I N E G F E L L R S G N S **

350 360

ATT intron 8 (1642 bp) CTTTACCTCCCAAAG C ATC CGC ACT ATC CTG ATG TTC TGA TTTCCTGAACTGAAAGCTTTCCTGTGGTTGATAC

** I R T I L M F stop

370

CTTCACTTAATGTACAGCACAGAAACAATTTGGTATTAAGCAGTGTTCCAGCTAACTGGAAAACCAGTAAGCCTGAATTATGGGGTCATAACTGCTCTGTTAACAGCTTTGTAATATAAATCTTTCTCTGTATAAACAGAGATTTGTTTGAAATAATTATTACTTTTCCTCAGTACTTTTGCCCATATTTTGAATTTGTGAGGCGATACAGCATTACAGATTTATGACCGCTCATTCCTCTTTTTATGTGCAGTTACCGCTTTTAAAGGATAAGTAAACCTTAACATTGAATGTAAAATTTCTCAGGACACTTTTTTTTGAGCAATGCAAAATCAGAATAGTTCCCTGCTTTTCTTCTAAATGCATATCTCTTTGACTGTACAGTT**AATAAA**AACTTAAATAGTGCTGAAACTTAAAAAAAAAACAAAAAAAAAAATTCATGTAAATTGCAAAAGTGCTTAGAAAAGCACCCTGAGCAAAAGGAGGAGTTACGATTAGCATAATACAT
