# Supplementary material for: The Xenopus alcohol dehydrogenase gene family: characterization and comparative analysis incorporating amphibian and reptilian genomes
Source: BMC Genomics. 2014 Mar 20;15:216. doi: 10.1186/1471-2164-15-216 (PMC4028059; doi:10.1186/1471-2164-15-216)
Supplement: Additional file 9 — Xenopus tropicalis ADH8A cDNA sequence. The sequence includes the translated coding exons, intron flanking regions (±15 bp with total intron size), and the proximal promoter (-600 bp from the ATG codon) and 3′-untranslated region (650 bp) with predicted regulatory elements. Putative TATA boxes and polyadenylation signals are in bold and underlined. Putative transcription factor binding sites are underlined, with the core sequence of the matrix in bold and italics (for overlapping sites, the most downstream site is overlined); and the orientation (+ or - strand) is given in parentheses. [file 1471-2164-15-216-S9.doc]

***X. tropicalis ADH8A***

**-600**

GTAATTAGAATTGCTCCTGCCTATGAATTTTCTCTCCTGTAAAAAGTGCCATTTAAGGCCCAGTTTCACTGCCCATGTCTGCCCACAAACTCATGTCATACTCCTTA

ACCATCTGGAGCG***AGATAA***GGTGCTTGTGGACAACCTGCAGTCAAATTATGTCTGC***TTACG***TGAAATAACAACATTCAATCATTCTAAACA***AGATAA***AT***AAATA***AAA

GATA1(+) CRE-BP1(+) GATA1(+) HNF3B(-)

TAAATTCTAAATAATAAATGAAAAAAAATTGTAAAGTTA***TTTAT***ATTAATTCCACCTTCAGCATTTACACTGACCATTCCAGTTAATAACAGT***TGACT***GGCGGAAAG

XFD2(-) AP1(+)

***AGTCA***AACATGCTGATAGCACTATTACACAGCATTTTTTCTCTGTTAGCTCACACCTAATATTTCGCAGCCCTGTTTTTAACCCTTCACCCAGAAATAGGTAGAATG

AP1(-)

CCACAATGGAATGCACTGCACTCTTTACAGGCATGTGGAGTTAAAGACTGAATATGAATTGAG***CCAAT***CATATGCC**TATA**TACTGCTTACAGGGAGTGGTCCCAGCT

CCAAT box(+) TATA box?

CC**TATA**TAGCACTCTCTGTTT***AGTCA***CTGAAAAGAGCAGAAAGACTTGCTGAAGCAGCCGGAGAC ATG GAC ACA ACA GGA AAA GTATTTGCTCTTTCA

TATA box AP1(-) M D T T G K **

1

intron 1 (1308 bp) TTATTTTTGTTTTAG GTG ATC AAA TGT AAG GCG GCT ATA GCA TGG GGA CTA CAC AAA CCA CTC ACG ATT

** V I K C K A A I A W G L H K P L T I

10 20

GAG GAA ATT GAA GTT GCA CCC CCA AAA GCT CAT GAA GTC AGA ATA AAG GTACAGTTATTCAAA intron 2 (510 bp) TACTGTGC

E E I E V A P P K A H E V R I K **

30 40

ATTGTAG ATT TTG GCA TCT GGT ATC TGT GGC TCA GAC CTC AGT GTA CTG AAA GAT AAA CTT AGT GGT GTT AAA TTT CCA ACT

** I L A S G I C G S D L S V L K D K L S G V K F P T

50 60

ATT TTG GGC CAT GAA GCC ATT GGC ATT GTT GAA AGT ATC GGT AAG GAT GTA ACT GTC GTT AAA CCA G GTGAGTATTGTATAT

I L G H E A I G I V E S I G K D V T V V K P **

70 80

intron 3 (458 bp) ACTTGCTTCCTTTAG GT GAC AAA GCA ATC CCA TTG TTT GTA CCT CAG TGT GGA CAA TGC AGA GCT TGC

** G D K A I P L F V P Q C G Q C R A C

90 100

AAG ACT CCA AAC TGT AAT GTG TGT GAA AAA AAT GA GTAAGTTCTACTTTT intron 4 (578 bp) TTTTTTCTCTACCAG C TTT

K T P N C N V C E K N D ** ** F

110

ACT ACC AAA AAA GGG CTG ATG CAA GAC AAT ACC AGC AGA TTT ACA TGC AAA GGC GAA CAA GTC TAT CAT TTT ATG AGC ACA

T T K K G L M Q D N T S R F T C K G E Q V Y H F M S T

120 130 140

AGT ACT TTC ACA GAA TAC ACT GTT GTG CCT GAC ATA TGT GTT GCC AAA GTG GAC CCT GCT GCT CCT GTT GAA GGC TGC CTT

S T F T E Y T V V P D I C V A K V D P A A P V E G C L

150 160 170

ATT GGC TGT GGA TTT GCT ACT GGA TAT GGT GCA GCA GTG AAC ACC GCT AAG GTAACTAGAGACATA intron 5 (528 bp) TTGT

I G C G F A T G Y G A A V N T A K **

180

ATTTCTTGCAG GTT ACT CCA GGA TCT ACA TGT GCT GTG TTC GGT TTA GGA GGT GTG GGG TTT TCA GCT TTG ATT GGC TGC AAA

** V T P G S T C A V F G L G G V G F S A L I G C K

190 200 210

ATC GCT GGT GCA GGT CGG ATT ATT GGA GTT GGC TCA CAT AAG GAT AAA TTC CCA AAA GCA ATA GAG TTG GGA GCC ACC GAA

I A G A G R I I G V G S H K D K F P K A I E L G A T E

220 230 240

TGC TTG AGC CCA AAG GAT TAT GAC AAA CCA ATT CAA GAG GTC ATT AGA GAC ATG ACT AAT GGC GGA GTG GAT TTT GCC TTT

C L S P K D Y D K P I Q E V I R D M T N G G V D F A F

250 260

GAA TGC ACT GGG TAT ATT GAA ACA ATG GTAAGGATTTTTGTT intron 6 (2229 bb) TTTGTATTTATTTAG AAA ACT GCC TTT GAC

E C T G Y I E T M ** ** K T A F D

270 280

TCA ACT TAT TTA GGC AAT GGG GTC ACC GTG GTG CTT GGA GTA GCA GGT CCA GAT GAT AGA CTT TCT TTT CAT CCT GGT GAA

S T Y L G N G V T V V L G V A G P D D R L S F H P G E

290 300

CTT TTG TTT GGA CGT ACC ATG AAA GGA TCA GCA TTT GGA G GTACTGAACAAAAAA intron 7 (274 bp) TCATTATATGCATAG GT

L L F G R T M K G S A F G ** ** G

310 320

TTT AAA AGC AGG GAT GAA GTA CCA ATG CTG GTT TCT GAT TAT ATG GAA AAG AAA TTT AAC CTT GAT TTC ATG GTG AGC GAA

F K S R D E V P M L V S D Y M E K K F N L D F M V S E

330 340

AGA ATA CCA TTG GAA AAA ATT AAT GAA GCA TTT GAA TTG ATG CAA AGT GGC AAA GG GTATGTATTGTCAAA intron 8

R I P L E K I N E A F E L M Q S G K G **

350 360

(1248 bp) TCTTTCTTTTTTCAG A GTG CGG AAC ATT ATT ATT TTC TGA TGCATTTTCCATTTCTCATGTGAAGTCCACTACATTGCTGCATCC

** V R N I I I F stop

370

TGACATCCTGATGTTTATTATTGATTATTTATAAGGTGATGATAGATAATATAATGATGCAATGCAACATCAACAACAACAATCTTAACATGATGGGGAAAAGAAATTAAAAGAAAGCATGTGAATTGGCTGATAATTGATTTTGCTTATATGAAGAAATGTCTATGTATTTTATATTATGTTATCTTAG**AATAAA**AAAATCTTTGCTACCACATAGAGTCTGAAGTTTACAATAATATCTTTAAACATATTCATTTTGTGTCTGCTTATGTCACATAAAAGTAACAGGAATTGTATTTAGAGCATTTTGGGGCAGATATTTTATTCAGATAGGCTGGATAGGATAGCTCCATAGTAATAATGATAGTCAC**AATAAA**AGAAGGAAATCCTAAAATATATTTATCTATATGCTCCCATAATGACATGGTTCCAATTTATGCAAGACTTACAAACAGGTGCAGAAATTGCACCCATTTTTGTGCATCACTGAGTTTTTTT
