# Supplementary material for: The Xenopus alcohol dehydrogenase gene family: characterization and comparative analysis incorporating amphibian and reptilian genomes
Source: BMC Genomics. 2014 Mar 20;15:216. doi: 10.1186/1471-2164-15-216 (PMC4028059; doi:10.1186/1471-2164-15-216)
Supplement: Additional file 10 — Xenopus tropicalis ADH8B cDNA sequence. The sequence includes the translated coding exons, intron flanking regions (±15 bp with total intron size), and the proximal promoter (-600 bp from the ATG codon) and 3′-untranslated region (650 bp) with predicted regulatory elements. Putative TATA boxes and polyadenylation signals are in bold and underlined. Putative transcription factor binding sites are underlined, with the core sequence of the matrix in bold and italics (for overlapping sites, the most downstream site is overlined); and the orientation (+ or - strand) is given in parentheses. [file 1471-2164-15-216-S10.doc]

***X. tropicalis ADH8B***

**-600**

AGTTAACCTATAG***GTAAACA***ACCCCTTTACCTGCAACAGACATGACAAAGGCTGTAACAGAAAGTTAGTGCTGGAAACCAGAACCAGTTATGGCTGCATGCAAGGAA

HNF3B(-) XFD1(+)

GCAGAGCAGTAGCTGCAGCAAAGTAGCAGAGATGAGACTGGAAGAACTTA***GGTCA***AATAACAAAGTACAATCATTCTAAACAACAACAAAAAAATTTGGTAAGTTTC

RORA1(+) ER(-)

TTTCGATTGTTATACCTAAAACGTACATTTTTAAGGATACTTGTGTAATGGAGTCGAAGAAAGAGTTAAACTTCCTGATTCCAT***TTATC***AGTCCACACCTAATGTTA

GATA1(-)

GAC***AAACA***CTTTTCTTTAACCC***AGTCA***CTCAAAAAATAGATGCACCA***AGATA***ATAATTC***TGTTT***GCTTAAGAAGGC***AGTCA***GAGTTAAAAACTTTTTGGATACAAAT

HFH3(-) AP1(-) GATA1(+) HNF3B(+) AP1(-)

CAAA***CCAAT***CGTATGCCTGAATACTGCTCAAAAGGCGTAGTCCCAGTTCA**TAAA**AAGCACTCTTCATTCAGGCTTGTCACTGAAAGGAACACTTGCTGAAGACGCCA

CCAAT box(+) TATA box

CAGAC ATG GAT ACT GCA GGA CAA GTACTTACACCTTTA intron 1 (371 bp) CTATTTATGTTTAAG GTG ATC AAA TGT AAG GCG GCT

M D T A G Q ** ** V I K C K A A

1 10

ATT GCA TGG GGA GAT CAC AAA CCA CTT ACA ATT GAG GAA ATT GAA GTC GCT CCC CCA AAA GCT AAT GAA GTC AGA ATT AAG

I A W G D H K P L T I E E I E V A P P K A N E V R I K

20 30 40

GTACTGTGATTTTTA intron 2 (1912 bp) TTTCTTGTATTGTAG ATT CTG GCA TCT GGT ATC TGT GGC ACT GAC ACT GCT GCA CTG

** ** I L A S G I C G T D T A A L

50

AAA GGT AAA CTT GGT ACT AAA TTT CCA GCT ATT TTG GGT CAT GAA GCC ATT GGC ATC GTT GAA AGC ATC GGT AAC GGT GTG

K G K L G T K F P A I L G H E A I G I V E S I G N G V

60 70 80

ACT ACT GTT CAA CCA G GTAAGTTTTGTATGA intron 3 (610 bp) TCTTACTTGCTTTAG GT GAC AAA GTA ATC CCA CTC TGT ATG

T T V Q P ** ** G D K V I P L C M

90

CCT CAG TGT GGA CAA TGC AGA GCA TGC AAG AGT CCA AAT GCT AAC CTG TGT GAC AAA AAT GA GTAAGTTCTACTTTT intron 4

P Q C G Q C R A C K S P N A N L C D K N D **

100 110

(1063 bp) TTATATCTTTACCAG C TTT ACT AAC AAC ACG GGG CTG ATG CAA GAC AAA ACC AGC AGA TTT ACA TGC AAA GGC AAA

** F T N N T G L M Q D K T S R F T C K G K

120 130

CAA GTC TAT CAT TTT GTG GGC ACA AGT ACT TTC ACA GAA TAC ACT GTT GTT TCT GAA ATA AGT GTT GCC AAA GTG GAC CCT

Q V Y H F V G T S T F T E Y T V V S E I S V A K V D P

140 150 160

GCT GCT CCT CTT GAG GTC TGC ATT GTT GGC TGT GGC TTT GCT ACT GGA TAT GGT GCA GCA GTG AAT TCT AAG GTAAACGGGC

A A P L E V C I V G C G F A T G Y G A A V N S K **

170 180

TATAG intron 5 (288 bp) ATATTTATTTTCCAG ATT GCT CCA GGA TCT ACA TGT GCT GTG TTT GGT TTA GGC GGT GTG GGG TTT

** I A P G S T C A V F G L G G V G F

190 200

TCA GCT TTG ATT GGC TGC AAA ATC TCT GGT GCA GGT CGG ATT ATT GGA GTT GGC TCA CAT AAG GAT AAA TTC CCA AAA GCC

S A L I G C K I S G A G R I I G V G S H K D K F P K A

210 220 230

ATA GAG TTG GGA GCC ACC GAG TGC TTG AGC CCA AAG GAT AAT GAC AAA CCA ATT CAA GAG GTC ATT AGA GAC ATG ACT AAT

I E L G A T E C L S P K D N D K P I Q E V I R D M T N

240 250

GGC GGA GTG GAT TTT GCC TTT GAA TGT AGT GGA AAT ATT GAA ACA CTG GTAAGGTTTTTTTTC intron 6(2309 bp) ATATTCTTT

G G V D F A F E C S G N I E T L **

260 270

ATTTAG AAA ACT GCA TTT GAA TCA ACT TAC ATA GGT AGT GGT GTC ACT GTG TTG CTG GGA GTC GCA GGT CCG AAT GAT AAA

** K T A F E S T Y I G S G V T V L L G V A G P N D K

280 290

CTT TGT TTT CAT CCT GGT GAA GTC ATG ATG GGA CGA ACC ATA AAA GGA TTA CCA TAT GGA G GTAGAGAACTATGAA intron 7

L C F H P G E V M M G R T I K G L P Y G **

300 310

(2866 bp) AAATTTTATACATAG GA TTT AAG GGC AGG GAT GAT ATA CCA AAG CTC GTT GGT GAT TAT ATG GCA AAC AAA TTT

** G F K G R D D I P K L V G D Y M A N K F

320 330

AAC CTG AAT TTC ATG GTG AGC GAA AGA ATG CCA TTG GAA AAA ATC AAT GAA GCA TTT GAA TTG ATG GCA AGT GGG AAA GG

N L N F M V S E R M P L E K I N E A F E L M A S G K G

340 350 360

GTATGTATTGTCTTA intron 8 (1166 bp) TTCTCGTTTTTTCAG A CTG CGG AAC CTT ATC ATT TTC TAA TGCCATTTCCATTTTTCGT

** ** L R N L I I F stop

370

GTGAAGACCACTACGGTGTTGCAGCATGACATCCCATTGATTATAATTTTTTCAATACTACAAACAGATAATACAATGACTAAATGGAATAATGAGTTTATACTGCC**AATAAA**AGAAAATGTACTAAATATAATGGGGAAAAGGATGTAATGGATAGTCCATACTTTCCCTTAGTGGGGTTAAAAATGTTTAATTTTGCTTATGAGAAGGCACTTATGAAGATATATTATACATTGTTACCTTTCCATTTGGTAGAAAGAATAATTGCATATTAGTGATAGTCATAATAATGGAGCAAGGGAATACTTTACACCTGCCATTGACCAGGCACTTTTTTATGCTTAAAACTTTACATACGATTGGATTTTGTATGAGAGTTTATTTTGCCCCTCCCACATGCTGCCAGATAAATGTGGCTCCAATTTATGCAAAACAGGTGCTAAAACTGCCCCGTGTATGCACATCGCAAATACAACTGAACATAAAGTAAGAGTGCAGCTAGTCCACCTTGAAATGTAAGTTTT
