# Supplementary material for: The Xenopus alcohol dehydrogenase gene family: characterization and comparative analysis incorporating amphibian and reptilian genomes
Source: BMC Genomics. 2014 Mar 20;15:216. doi: 10.1186/1471-2164-15-216 (PMC4028059; doi:10.1186/1471-2164-15-216)
Supplement: Additional file 11 — Xenopus tropicalis ADH9 cDNA sequence. The sequence includes the translated coding exons, intron flanking regions (±15 bp with total intron size), and the proximal promoter (-600 bp from the ATG codon) and 3′-untranslated region (650 bp) with predicted regulatory elements. Putative TATA boxes and polyadenylation signals are in bold and underlined. Putative transcription factor binding sites are underlined, with the core sequence of the matrix in bold and italics (for overlapping sites, the most downstream site is overlined); and the orientation (+ or - strand) is given in parentheses. [file 1471-2164-15-216-S11.doc]

***X. tropicalis ADH9***

**-600**

CAGGCGTTTTCTTTACACAGAGAATATTGCTATATACTAC***AGATA***TAATTTTTTGGTTGTTAGGCAAGAAGCTGACAGAAGAATTGTGCATTACACAAGGCTAATGG

GATA1(+)

CAGCACTGATCTTTTGAAATATATACTGTAATAAGTGCATTTTGTGCAAGTTGAATGAAACCCAAGATGGCATTCTGGCAAATAATTCTGCATTGTGAGTAAAAAAC

GTTGATTGATGTAATAAAATTAATGCTGCATCTATAAATGAGTCCTTGTCACTTTAAGTTTGCACATTTAACTGATCTGCATTGATTAAACCGCTTACATGCTAT***GC***

OCT1(+)

***AAA***AC***AAATA***GAATTGAACTTTTCATACCCTAACATGATCTTGCAA***ATAAACA***CTACACTACAGATTTCTACTTTGCCCTTAGCGATATTTGGGGCTCTATGTTAGG

HNF3B(-) HNF3B(-) XFD2(+)

ACTTTAAGTTTACTTTGGTTTACTTAGCTGTG***CCAAT***CTGAAGGGCGTAGCCATCAAAGTAACTGATCTG**TATA*TATGC***AGTACAGAACTTAGTCTGTCTCTGCAAG

CCAAT box TATA box OCT1(+)

AAACCAGAAAAGACCCAACAAACT***GATAA***GGACTTTTAACTGCTGTGTGCAGCAAGAAAGCCAAG ATG TCC ACT GTT GGA AAG GTAAATCATATAATT

GATA1(+) M S T V G K **

1

intron 1 (4113 bp) CTACATTACGCCTAG GTT ATC CAC TGC AAA GCA GCA GTT GCC CTG GAG GCT AAA AAA CCA CTT GTT ATA

** V I H C K A A V A L E A K K P L V I

10 20

CAG CAA ATT GAA GTT GCT CCA CCA AAG GCT AAA GAA GTC CGT ATC AAG GTAACAAAAGCACAA intron 2 (178 bp) TGCTGTTT

Q Q I E V A P P K A K E V R I K **

30 40

TCTTTAG ATT TAC CAC AGT GGC ATT TGC CAC ACA GAT GAC CAT GCT CTA GGG GGC TTT ATG GCT GGT ATT ACA TTT CCA GTC

** I Y H S G I C H T D D H A L G G F M A G I T F P V

50 60

ATT TTG GGA CAC GAG GGA GCT GGA GTT GTT GAG AGC GTT GGA GAA GGA GTA ACA ACT GTG AAA CCA G GTATTTTAATTTGTT

I L G H E G A G V V E S V G E G V T T V K P **

70 80

intron 3 (645 bp) TCCTTTTTCCCTTAG GA GAT CAT GTC ATC GCA ATA TGC AGT CCA ATG TGC ATG AAG TGT CCC AGC TGC

** G D H V I A I C S P M C M K C P S C

90 100

TTG CAT CCC GAC AGT AAC TTT TGT GTT AAA AAT GA GTATGTGTGTATTTA intron 4 (1353 pb) GTGTTTCTTGATCAG T GTT GGC

L H P D S N F C V K N D ** ** V G

110

AAG CAT GTT GGA CTG ATG CTG GAC AAA ACC AGC AGA TTC TCC ATC AAG GGC AAG CTA ATT CAC AAC TTT ATG TCC ACA AGC

K H V G L M L D K T S R F S I K G K L I H N F M S T S

120 130 140

ACA TTC TGC GAA TAC ACC GTT GTG GAT GAA TTT GCT TGT GTC AAG ATT GAC CCC AAG GCT CCC TTG CAT GAA GTG TGT CTG

T F C E Y T V V D E F A C V K I D P K A P L H E V C L

150 160 170

ATT GGC TGT GGG TTT TCC ACT GGT TAT GGA TCA GTA CTC AAC ACA GCT AAA GTAAGCAAGGACATT intron 5 (1273 bp) TTAA

I G C G F S T G Y G S V L N T A K **

180 190

TGTTATACCAG GTA CAG CCA GGT AGC GCA TGT GCA GTG TTT GGT TTG GGT GGA ATA GGA ATG TCT GTT GTT ATG GGC TGC AAA

** V Q P G S A C A V F G L G G I G M S V V M G C K

200 210

GTT GCT GGA GCA TGC CGA ATC ATT GGA GTA GAC ATC AAC AAA GGA AAA TTT GAA ATT GCA AAG AAG TTG GGA TGC ACT GAG

V A G A C R I I G V D I N K G K F E I A K K L G C T E

220 230 240

TGC CTG GAC CCA AAC GAT TAT GAT AAA CCC ATC CAT GAG GTT ATT GCA AAT ATG ACT GAT GGG GGA GTT GAT TAT TCA TTT

C L D P N D Y D K P I H E V I A N M T D G G V D Y S F

250 260

GAA TGC GTT GGA AAT GTT GAT TTG ATG GTAAGTAATATATTA intron 6 (1590 bp) TTTATTTTCCCTTAG GCA TGT GTT ATT CAA

E C V G N V D L M ** ** A C V I Q

270 280

TCC TGT CAT TAT TCA TAC GGT TGT GCA ACC ATT ATT GGT GTC CCG CCA TCA ACA GCT CGA CTT TGC CTT GAT CTG ATA TGG

S C H Y S Y G C A T I I G V P P S T A R L C L D L I W

290 300

TTA CTC ACT GGG CGC ACA TTG AAA GGA GCT TTC CTT GGA G GTACAGTATGCTACA intron 7 (1211 bp) TTGTCTGTTATTTAG AC

L L T G R T L K G A F L G ** ** D

310 320

TAC AAA GCA AAG GAG GCT TTC CCT GGC TTA GTA AAA GAT GCC ATG AAT AAA AAA TTT GAC ATT GGA GCA TTG GTG ACG CAT

Y K A K E A F P G L V K D A M N K K F D I G A L V T H

330 340 350

AGG GTG AAG TTT GAT AAA ATT ATG GAT GGC TTT GAA CTC ATG CGC CAT GGA AAA TG GTGAGCTCTTGGTGT intron 8

R V K F D K I M D G F E L M R H G K C **

360

(460 bp) TTTTTTGTTTTACAG T GTG CGT GCA GTC CTG GAT ATG TGA AGCAGAAATCGAAAGCTTGGAATGAACACAATCATATGTTGTCTCCT

** V R A V L D M stop

370

CAACAAAATGCTTGTAGATAGAAGTGCATAGTAGAATCAAGCAAGAAAATAGTCTGTTGCTTACTGATGTTCTAATTTTGCTTACTGAAATTTTAAAGCAAAAAAAAAAATATTC**AATAAA**TTTTACAGGTTGATTTATAAAAAAATGTCAAGTGTTTTTGTCTGTTTTATTTTACTACTAAACTATGCTGCATAATAGAGAAATTGTGAATCTTTGTACATGAATGAA**AATAAA**AAGCTGTGCATGTTGGTCAGACATACAATGGGTCAGATGTAAATGCAGCCAGATGGATCAAACTCTGGAACAGTTTTGGAGCAGAACAGTGCAGTTTTAGCAGCATGAAAAAACAGTTGCATTAAAATTTGAATCAAAGATAAAATTTTGTCATATTATTTCATGATAATTGGTTTATTGTAATTGTCACACAAAACCAGGGTCAGACTGGGGTGTCTAGCACCCACAGGGACTGCAACTTCAAGGGCCTTCTTGCAAACT
