# Supplementary material for: The Xenopus alcohol dehydrogenase gene family: characterization and comparative analysis incorporating amphibian and reptilian genomes
Source: BMC Genomics. 2014 Mar 20;15:216. doi: 10.1186/1471-2164-15-216 (PMC4028059; doi:10.1186/1471-2164-15-216)
Supplement: Additional file 12 — Xenopus tropicalis ADH10A cDNA sequence. The sequence includes the translated coding exons, intron flanking regions (±15 bp with total intron size), and the proximal promoter (-600 bp from the ATG codon) and 3′-untranslated region (650 bp) with predicted regulatory elements. Putative TATA boxes and polyadenylation signals are in bold and underlined. Putative transcription factor binding sites are underlined, with the core sequence of the matrix in bold and italics (for overlapping sites, the most downstream site is overlined); and the orientation (+ or - strand) is given in parentheses. [file 1471-2164-15-216-S12.doc]

***X. tropicalis ADH10A***

**-600**

ACGCACACCATGTTAGCCATCCATGCCAATACTTTCGGAAAATTACTGTACTGAAAATGACCA***TATCT***CCGCAAACTGGAGGATGCATCACTCTAGGGCCAACACAG

GATA1(-)

ACTTGAATAAATCACACTGCAAAGTCCATATTGTGTTGCCAAAAGCTCATTAACTGTACTTTTCTATAATTACCTCCTGCCTCTAGTAGGTAGGCAAATTAACGCAT

GCGATATGCGACTTAACG***CACGT***GGTAATACTGTAGTGAATCGTGCGTTAATTTTCGCGTTTATTTTAACGCAAAAAAGCATGCAATAAAAAGGAACGCACTTTAGT

USF(+)

GAATCAACCCTATTGTAT***TGCAA***TATCATCTAGTTAATAATC***ATTAA***CTGTATGGAAAAAGTGAAGGACAAGAAAATTTATATTGTC***TGTTT***TTTTAGAAAACCAGT

CHOP:C/EBPA(+) HNF1(-) HNF3B(+)

TCAAAGGTTCATTGAATGCTGATCATTTGGCTCAAGTA***TGTTT***CTCTTTAGAAATT***ATTAA***CTTTGGGTTTATGAATCAG**TATA*TATCT***ACTTTATGCGTCCAGATC

HFH3(+) HNF1(-) TATA box GATA1(-)

CAACAAATCAACAGCAGAAACTGCTTGGCACTTAAGTTTGTTTCT***TGTTT***GTAAAAATACAGAGA ATG GAT ACA GCA GGA AAG GTAAGTGCATTTAGA

HFH3(+) M D T A G K **

1

intron 1 (1541 bp) AATTTATTTGTAAAG GTG ATA AAA TGC AAA GCA GCA GTG ATA TGG GAG AAA AGT GCA CCC TTC TCT ATT

** V I K C K A A V I W E K S A P F S I

10 20

GAG GAA GTT GAA GTG GCC CCC CCA AAG GCC AAT GAA GTC CGT ATA AAG GTAAGCTTCTTAATG intron 2 (1006 bp) TATCTTAT

E E V E V A P P K A N E V R I K **

30 40

ATTATAG ATT GTC GCT ACT GGT ATT TGC CGG TCC GAT GAT CAT GCA ATT GAG GGG AAA CTG AGT ACA GTA AAA TTC CCA GTT

** I V A T G I C R S D D H A I E G K L S T V K F P V

50 60

ATA CTT GGC CAT GAA GGT GTT GGT ATT GTG GAG AGC ACT GGA GAA AGT GTT AAG CAC ATT AAG CCA G GTATGTGGGGAAACT

I L G H E G V G I V E S T G E S V K H I K P **

70 80

intron 3 (1006 bp) GTTTTCTTTCTCTAG GA GAT AGA GTC ATC CCA CTT TTT GTA CCT CAA TGT GGA CAA TGT ATA TGT TGC

** G D R V I P L F V P Q C G Q C I C C

90 100

AAA GAC CCA AGG AGT AAT ATG TGC ATT GCA GGC AA GTAAGTACCAGTATT intron 4 (593 bp) ATGGTGTTAAAACAG A ATG AAA

K D P R S N M C I A G K ** ** M K

110

AAA GCT GTA GGA CTC ATG TCG GAC GGC ACC AGC AGA TTT ACC TGC AAA GGG AAG CAA ATC TAC CAC TTC ATG AAC ACC AGC

K A V G L M S D G T S R F T C K G K Q I Y H F M N T S

120 130 140

ACT TTT ACT GAA TAC ACT GTA GCT GAG GAA ATG TCA GTT GCT AAG ATC GAC AGT GGT GCA TCC CTA GAT AAT ACC TGT CTC

T F T E Y T V A E E M S V A K I D S G A S L D N T C L

150 160 170

ATT GGA TGT GGC TTC TCC ACT GGA TAT GGG TCT GCA TTA AAT TCC GCC AAG GTAAATATTTATTTG intron 5 (3002 bp) TATG

I G C G F S T G Y G S A L N S A K **

180 190

ATCTTTTGCAG GTG CAC CCA GGG TCT ACT TGT GCC ATA TTT GGT TTA GGA GGA ATT GGC CTT GCT GTC ATT ATG GGC TGT AAG

** V H P G S T C A I F G L G G I G L A V I M G C K

200 210

ATA GCT GGG GCA GCT CGC ATC ATT GGA GTA GAT ATC AAT CCT GAC AAA TTC AAC ATA GCA AAG GAA CTA GGA GCG ACT GAA

I A G A A R I I G V D I N P D K F N I A K E L G A T E

220 230 240

TGT ATA AAC CCC AAG GAT TAT GAT AAA CCA GTA CCA CAA GTG ATT CTG GAG CAG ACT GGA GGT GGC GTG GAC TAT GCA TTT

C I N P K D Y D K P V P Q V I L E Q T G G G V D Y A F

250 260

GAA TGT GTT GGG CAC ATT GAG ACC ATG GTAAGTAGTTAGGCT intron 6 (561 bp) TGATTCCTTTTCCAG CTA GCT GCC CTT AAC

E C V G H I E T M ** ** L A A L N

270 280

TCA AGT CAT TTT GCA TAT GGC ACA ACA GTG ATA GTC GGA GTG TCT GCA CCA GAT TCA ACC ATC TCC TTT GAT CCA ATG ATC

S S H F A Y G T T V I V G V S A P D S T I S F D P M I

290 300

CTG CTG ACT GGG CGC ACA ATT AAA GGC TCT GTC TTT GGA G GTATTATAAGTTTTC intron 7 (4698 bp) CTCGTTCTTTTCTAG GC

L L T G R T I K G S V F G ** ** G

310 320

TGG AAG AGT AAG AAT TCT GTT CCA CAA CTG GTT TCA GAT TCT TTG GCA AAG AAA TTC GAC CTT GAA AAG TTG GTA ACC CAT

W K S K N S V P Q L V S D S L A K K F D L E K L V T H

330 340 350

CGA TTA CCA TTA AAT AAA ATT AAT GAA GGG TTT GAT CTT TTA CGT TCT GGA AAG AG GTAAAACGTGTTTTT intron 8

R L P L N K I N E G F D L L R S G K S **
 360

(1132 bp) TATCTTTTTTTTCAG T ATT CGA ACA ATC TTG TTG GTT TAA ACAATGGAAAGATATTGCATTTACCACCACAGAATTATCTAAGATGC

** I R T I L L V stop

370

ACTTCTTCGTGTTCAAGTATCAGGATACATTTCTTGAAAA**AATAAA**GTCAAAAACAATTTTATTTACTGCAGTGCGAATTTTTAAACAAAAAATTATTTTTTCAAAATATAAAACAT**AATAAA**CTGAATATAGGGCAAATTCTCATAAGTAACAATGTGTTCTGCATTTGCAA**AATAAA**GGCTTAATCTGACCAAGTTCTGCTTGTTTGGTAAGGTGGCAATGACATTCTTCAACCCAAACATGGCCTAGGCCAGGGAATAATATTCCCTATGTGTTCCCTATATACCCCTCAGGAACACCTAGAAATCAAGGACAATGAGATCCATTTTTCAAATACAAGTTA**AATAAA**AACACGGTATAGATTACATCAAGTTTATTTTTAGCAATTTTAA**AATAAA**GAGCTGAGCTGTACTGTACAGACATGCACTATTCTTCTATCTGGAAGAGAAATACAGCATTATAATATTAAGCGTCTGCTTTGTGTCTCCTTCTTGT
