# Supplementary material for: The Xenopus alcohol dehydrogenase gene family: characterization and comparative analysis incorporating amphibian and reptilian genomes
Source: BMC Genomics. 2014 Mar 20;15:216. doi: 10.1186/1471-2164-15-216 (PMC4028059; doi:10.1186/1471-2164-15-216)
Supplement: Additional file 13 — Xenopus tropicalis ADH10B cDNA sequence. The sequence includes the translated coding exons, intron flanking regions (±15 bp with total intron size), and the proximal promoter (-600 bp from the ATG codon) and 3′-untranslated region (650 bp) with predicted regulatory elements. Putative TATA boxes and polyadenylation signals are in bold and underlined. Putative transcription factor binding sites are underlined, with the core sequence of the matrix in bold and italics (for overlapping sites, the most downstream site is overlined); and the orientation (+ or - strand) is given in parentheses. [file 1471-2164-15-216-S13.doc]

***X. tropicalis ADH10B***

**-600**

AATACATAGGGGCAGATTTACTAAAACTGTAATACTTCTCATATTTGTATTTTAAAAATTCTACTAAACTCATTTTCACAAATGTTTCCAAAGTAAATTTGAACTGA

AAAGGTCTGCGC***TGACC***ACTTTTCTTGGAACTGCAACAAACCGGTGGATGTATTTTTAT***TGACT***TTCAGAACTTTACCTGGTTGTGCTCAGTAATGGTGATCAACCA

ER(+) AP1(+)

CTCTGGCTGTTTTTGGCTACTACACATGCATTTATTGCC***CCAAT***TCCATCTGCTATATAAGTTTTCGGCAAATGCATTTTCTAAAGGTGCCATGATACTACCACACA

CCAAT box

GTGAATAATATACTGGTATAAATACTGATTTGGTGTCCATGTCACCTAGTT***GATAA***GCACTACTGTCCAGACAATGATTAAATTAAGGCAAATAAATTTTTTGTACA

GATA1(+)

CTTGATCTTGCATTTTAGAAAATATGTCCAAAGGTTTCTTGCTCAGTTTTTTTCTGTTAAATA***ATTAA***CTTTTTG***TTATC***CTAAAAAG**TATA**TATTATGTGTACAGA

HNF1(-) GATA1(-) TATA box

TCATCTAAATCTGTTTCATTCTTGTCTGGTGTTGCTGAAATTTATAAGAAAAAATTGTAAACTAA ATG GAA TCA GCA GGA CAA GTAAGTGCTTATTTA

M E S A G Q **

1

intron 1 (1912 bp) ATGCACCTTTTTTAG GTT ATT AAA TGC AAG GCG GCT GTG ACA TGG GAA AAA GAT GCA CCC TTT TCA ATT

** V I K C K A A V T W E K D A P F S I

10 20

GAG GAG ATA GAA GTT GCT CCC CCA AAA GCC CAT GAA GTT CTG ATT AAG GTAAGATTCTTCCCA intron 2 (382 bp) ATACACTT

E E I E V A P P K A H E V R I K **

30 40

TATTAAG CTG ATA GCA ACA GGT ATC TGT CGG TCT GAT GAC CAT TCA CTC GAG GGG AAA TTT GCA GCT GTA AAA TTC CCA GTT

** L I A T G I C R S D D H S L E G K F A A V K F P V

50 60

ATT CTT GGC CAT GAG GGA GTT GGT ATT GTT GAA AGC ATT GGA GAC GGT GTG AAA GAC ATT AAG CCA G GTATGTGAAACTAAA

I L G H E G V G I V E S I G D G V K D I K P **

70 80

intron 3 (2172 bp) CTTCTTTTTCTTTAG GA GAC AAA GTC ATT CCA TTA GTT GCA CCT CAA TGT GGA AAA TGC CAG TGT TGC

** G D K V I P L V A P Q C G K C Q C C

90 100

AAG GAT CCA AGG ACT AAT AGA TGT CTC ACC AG GTAAGTATTTTCAAG intron 4 (890 bp) TTTTTTCATCACCAG A CTG AAA AGA

K D P R T N R C L T R ** ** L K R

110

CAA TTT GGA CTC ATG TCT GAT GGC ACC AGC AGA TTT ACC TGC AGA GGG AAG CAA ATC TAC CAC TTC ATG AAC ACC AGC ACT

Q F G L M S D G T S R F T C R G K Q I Y H F M N T S T

120 130 140

TTT ACT GAA TAC ACT GTA GTT GAG GAA ATG GCA GTT GCT AAG ATT GAT GAT AAT GCA ACA ATG GAT AGT GTC TGT CTC ATT

F T E Y T V V E E M A V A K I D D N A T M D S V C L I

150 160 170

GGA TGT GGT TTC TCC ACT GGT TAT GGG TCG GCA TTA AAT ACC GCC AAG GTAAATATATGTATA intron 5 (344 bp) TGTT

G C G F S T G Y G S A L N T A K **

180

TTCTATTGCAG GTG CAC CCA GAA TCT ACT TGT GCC ATA TTT GGT TTA GGA GGA ATT GGC CTT GCT GTC ATT ATG GGC TGT AAG

** V H P E S T C A I F G L G G I G L A V I M G C K

190 200 210

ATA GCT GGG GCA GCT CGC ATC ATT GGA GTA GAT ATC AAT CCT GAC AAA TTT GAC ATA GCA AAG GAA CTA GGA GCG ACT GAA

I A G A A R I I G V D I N P D K F D I A K E L G A T E

220 230 240

TGT ATA AAC CCC AAT GAT TAT GAT AAA CCA GTA GCA GAA ATG ATT CTG GAG CAG ACT GGA GGT GGC GTG GAC TAT GCA TTT

C I N P N D Y D K P V A E M I L E Q T G G G V D Y A F

250 260

GAA TGT GTT GGC CAT GCT GAA ACT ATG GTATGACTTGGGGAC intron 6 (382 bp) CACTTATTTTCCCAG TTA GCT GCA CTA CAC

E C V G H A E T M ** ** L A A L H

270 280

TCA AGT CAC TTT GCA TTT GGA ACA ACA GTC ATA ATT GGA GCA TCT GCA TCA ACT CTT TCC TTT GAT CCA ATG ATC CTG TTG

S S H F A F G T T V I I G A S A S T L S F D P M I L L

290 300

TCT GGA CGC ACA CTT AAA AGC TCT TCT TTT GGA G GTACAGCAGTTTGGT intron 7 (671 bp) CTGTACTATTTCTAG GC TGG AAG

S G R T L K S S S F G ** ** G W K

310 320

TCT AGG CTG GAA GTT CCA AAA TTA GTT TCT GAT TAT TTG GCA AAG AAA TTT GAC CTG GAA AAG TTG GTG ACG CAC CGA TTA

S R L E V P K L V S D Y L A K K F D L E K L V T H R L

330 340

CCT TTT CAG AAG ATC AGT GAA GGA TTT GAT CTT TTA CAT TCT GGC AAA TG GTATTTCACTTCCAC intron 8 (2730 bp) CT

P F Q K I S E G F D L L H S G K C **

350 360

GTTCTCCTTTCAG C ATT CGG ACA ATC CTG AAA TTT TAA GCAGTGGCTGCATTCTTCTCCAAGATGTCATTTCCATGAGCCA**AATAAAAATAAA**AACA

** I R T I L K F stop

370

ATTGTGTGATTTTTGTTTTTATCATGCGTACCTGGAACAAGTGTTTTGATAAAGCCATTACATATAATAACATGAACTAAACTGTACTGATAGTGTATGCACGTTCAGAGTGTATGGTGTTAATCCAAGTAGTAGCTATGGAGTGCCATTACACCGGGGCATATTTATAAAGCTGTGCAAAATTATTTACCCTAGAAAACCGTGTAAAAAAAGTGATGTATTTATGTCGTTAAGACAGTATTAGTTTAGCTTACAGCTTTATTGTGTTAAAAAGCCACTGTCTGCCTGAATGGCTAAAAGTTAGGTAACCCTATGTCAGGCTATGTTGTTTAAATGCTGGAAAACTGTGAAACTGAAGAGAAAGATGTGCACAAACTGAAAAAAAACCCATAACGTTCTAGAGGGTTTTAGGCCTCCTGTCCTACATTTAAAGGGATAGTGACACTTTCCAAATGTAGCTGGAACATGTTACAAGGGTAAGGTG
