# Supplementary material for: The Xenopus alcohol dehydrogenase gene family: characterization and comparative analysis incorporating amphibian and reptilian genomes
Source: BMC Genomics. 2014 Mar 20;15:216. doi: 10.1186/1471-2164-15-216 (PMC4028059; doi:10.1186/1471-2164-15-216)
Supplement: Additional file 14 — Alignment of amphibian ADH amino acid sequences. [file 1471-2164-15-216-S14.doc]

**Alignment of amphibian ADH amino acid sequences.** Only full-length sequences have been included. Sequence names consist of source organism (Horse: *Equus caballus*, as a reference, RP: *Rana perezi*, XL *Xenopus laevis*, XT: *Xenopus tropicalis*) followed by the assigned class number and enzyme notation.
