# Supplementary material for: The Xenopus alcohol dehydrogenase gene family: characterization and comparative analysis incorporating amphibian and reptilian genomes
Source: BMC Genomics. 2014 Mar 20;15:216. doi: 10.1186/1471-2164-15-216 (PMC4028059; doi:10.1186/1471-2164-15-216)
Supplement: Additional file 15 — Alignment of vertebrate ADHs included in the phylogenetic tree. [file 1471-2164-15-216-S15.doc]

**Alignment of vertebrate ADHs included in the phylogenetic tree.** Only full-length sequences have been included. ADH sequences from *Xenopus laevis*, *Xenopus tropicalis*, *Anolis* *carolinensis* (anole lizard) and *Pelodiscus sinensis* (turtle) are those described in this manuscript and their accession numbers are provided in Tables 3 and 4. Accession numbers of other ADH sequences are compiled in Table 2.

10 20 30 40 50 60 70 80 90 100

....|....| ....|....| ....|....| ....|....| ....|....| ....|....| ....|....| ....|....| ....|....| ....|....|

**Human 3**  **---ANEVIKC** **KAAVAWEAGK** **PLSIEEIEVA** **PPKAHEVRIK** **IIATAVCHTD** **AYTLSGADPE** **GC-FPVILGH** **EGAGIVESVG** **EGVTKLKA--** **GDTVIPLYIP**

**Rat 3**  **---ANQVIRC** **KAAVAWEAGK** **PLSIEEIEVA** **PPQAHEVRIK** **IIATAVCHTD** **AYTLSGADPE** **GC-FPVILGH** **EGAGIVESVG** **EGVTKLKA--** **GDTVIPLYIP**

**Mouse 3**  **---ANQVIRC** **KAAVAWEAGK** **PLSIEEIEVA** **PPKAHEVRIK** **ILATAVCHTD** **AYTLSGRDPE** **GC-FPVILGH** **EGAGIVESVG** **EGVTKLKA--** **GDTVIPLYIP**

**Rabbit 3**  **---ANKVIKC** **KAAVAWEAGK** **PLSIEEIEVA** **PPKAHEVRIK** **IFATAVCHTD** **AYTLSGADPE** **GC-FPVILGH** **EGAGIVESVG** **EGVTNLKA--** **GDTVIPLYIP**

**Chicken 3**  **---ASGVIKC** **KAAVAWEAGK** **PLSIEEVEVA** **PPKAHEVRIK** **IVATALCHTD** **AYTLSGADPE** **GC-FPVILGH** **EGAGIVESVG** **EGVTKVKP--** **GDTVIPLYIP**

**Turtle 3**  **---ASGVIKC** **KAAVAWEAGK** **PLSIEEVEVA** **PPKVHEVRIK** **VVATAVCHTD** **AYTLSGADPE** **GC-FPVILGH** **EGAGIVESVG** **EGVTKVKP--** **GDTVIPLYVP**

**Uromastyx 3**  **---ASGVIKC** **KAAVAWEAGK** **PLSIEEIEVA** **PPKAHEVRVK** **IIATAVCHTD** **AYTLSGADPE** **GS-FPVILGH** **EGAGIVESVG** **EGVTKFKP--** **GDTVIPLYIP**

**X.tropicalis 3**  **-ETAGKVVKC** **KAAVAWEAGK** **PLSIEEVEVA** **PPKAHEVRIK** **IVSTAVCHTD** **AYTLSGADPE** **GC-FPVILGH** **EGAGIVESVG** **EGVTRVKP--** **GDKVIPLYIP**

**X.laevis 3**  **-ETAGKVIKC** **KAAVAWEAGK** **PLSMEEVEVA** **PPKAHEVRIK** **IVSTAVCHTD** **AYTLSGADPE** **GC-FPVILGH** **EGAGIVESVG** **EGVTRVKP--** **GDKVIPLYIP**

**Anole 3**  **------VIKC** **KAAVAWESGK** **PLSIEEVEVA** **PPKAHEVRIR** **IIATAVCHTD** **AYTLSGADPE** **RE-FPCHPGH** **ERAGIVESVG** **EGVTKFKA--** **GDTVIPLYIP**

**Marmoset 2**  **-STKGKVIKC** **KAAIAWEAGK** **PLCIEEVEVA** **PPKAHEIRIQ** **IIATSLCHTD** **ATVIDSKFEG** **LA-FPVIVGH** **EAAGIVESIG** **PEVTNFKP--** **GDKVIPLYAP**

**Bovine 2**  **-GTKGKIIKC** **KAAIAWEANK** **PLSNEEVEVA** **PPKDHEVRIQ** **IIATALCHSD** **AHILHPQFEG** **GV-FPVILGH** **EAAGIVESIG** **PGVTNFKP--** **CDKVIPLHAP**

**Human 2**  **-GTKGKVIKC** **KAAIAWEAGK** **PLCIEEVEVA** **PPKAHEVRIQ** **IIATSLCHTD** **ASVIDSKFEG** **LA-FPVIVGH** **EAAGIVESIG** **PGVTNVKP--** **GDKVIPLYAP**

**Rat 2**  **-GTQGKVITC** **KAAIAWKTDS** **PLCIEEIEVS** **PPKAHEVRIK** **VIATCVCPTD** **INATNPKKK-** **AL-FPVVLGH** **ECAGIVESVG** **PGVTNFKP--** **GDKVIPFFAP**

**Mouse 2**  **-GTQGKVIKC** **KAAIAWKTGS** **PLCIEEIEVS** **PPKACEVRIQ** **VIATCVCPTD** **INATDPKKK-** **AL-FPVVLGH** **ECAGIVESVG** **PGVTNFKP--** **GDKVIPFFAP**

**Rabbit 2A**  **-GTKGKVIKC** **KAAIAWEAGK** **PLSIEEVEVA** **PPKAHEVRVQ** **INAAGLCRSD** **THVINPKFEG** **AF-LPVILGH** **EGAGIVESVG** **PGVTNVKP--** **GDKVIPLYIP**

**Rabbit 2B**  **-STKGKVIKC** **KAAIAWEAGK** **PLSIEEVEVA** **PPKAHEVRVQ** **IIAASVCRSD** **TYVINPAFKE** **GL-LPVILGH** **ECAGIVESVG** **PGVNNFKP--** **GDKVIPLYVP**

**Ostrich 2**  **-TTEGKVIKC** **KAAIAWEAGK** **PLSVEEIEVS** **PPKDHEVRVK** **IVATGVCRTD** **EHAINPSFKE** **GV-FPVILGH** **EGAGIVESIG** **QGVSKFKP--** **GDKVIPLYMP**

**Turtle 2**  **-STVGKVIKC** **KAAIAWEAGS** **LFSIEEVEVA** **PPKEHEIRVK** **IVATGVCRSD** **AHAISPSFKE** **GL-FPVILGH** **EGAGIVESTG** **PGVTKFKP--** **GDKVIPLYMP**

**X.tropicalis 2**  **-STARKVIKC** **KAAVAWEAGK** **PFSIEEIEVA** **PPRDHEVRVK** **IVATGVCRTD** **AHAVSTHFKE** **GL-FPVILGH** **EGAGIVESVG** **PGVTRVKP--** **GDKVIPLYIP**

**Human 4**  **-GTAGKVIKC** **KAAVLWEQKQ** **PFSIEEIEVA** **PPKTKEVRIK** **ILATGICRTD** **DHVIKGTMVS** **-K-FPVIVGH** **EATGIVESIG** **EGVTTVKP--** **GDKVIPLFLP**

**Rat 4**  **-DTAGKVIKC** **KAAVLWGTNQ** **PFSIEDIEVA** **PPKAKEVRVK** **ILATGICGTD** **DHVIKGTMVS** **-K-FPVIVGH** **EAVGIVESVG** **EEVTTVRP--** **GDKVIPLFLP**

**Mouse 4**  **-GTAGKVIKC** **KAAVLWGVNQ** **PFSIEEIEVA** **PPKAKEVRVK** **ILATGICRTD** **DHIIKGSMVS** **-K-FPVIVGH** **EAVGVVESVG** **EGVTTVRP--** **GDKVIPLFLP**

**Human 1B1**  **-STAGKVIKC** **KAAVLWEVKK** **PFSIEDVEVA** **PPKAYEVRIK** **MVAVGICRTD** **DHVVSGNLVT** **-P-LPVILGH** **EAAGIVESVG** **EGVTTVKP--** **GDKVIPLFTP**

**Rat 1**  **-STAGKVIKC** **KAAVLWEPHK** **PFTIEDIEVA** **PPKAHEVRIK** **MVATGVCRSD** **DHAVSGSLFT** **-P-LPAVLGH** **EGAGIVESIG** **EGVTCVKP--** **GDKVIPLFSP**

**Mouse 1**  **-STAGKVIKC** **KAAVLWELHK** **PFTIEDIEVA** **PPKAHEVRIK** **MVATGVCRSD** **DHVVSGTLVT** **-P-LPAVLGH** **EGAGIVESVG** **EGVTCVKP--** **GDKVIPLFSP**

**Rabbit 1**  **---AGKVIKC** **KAAVLWQLNK** **PFSIEEVEVA** **PPKAHEVRIK** **MVATGICRSD** **DHAVTGSIAV** **-P-LPVILGH** **EAAGIVESIG** **EGVTTVKP--** **GDKVIPLFTP**

**Ostrich 1**  **-STAGKVIKC** **KAAVLWEPKK** **PFSIEEVEVA** **PPKAHEVRVK** **IIATGICRSD** **DHVISGVLVM** **-P-FPIILGH** **EAAGVVESVG** **EGVTSVKP--** **GDKVIPLFVP**

**Quail 1**  **-STAGKVIKC** **KAAVLWEANK** **PFSLEEVEVA** **PPKAHEVRIK** **IVATGICRSD** **DHVVTGALAM** **-P-FPVILGH** **EAAGVVESVG** **EKVTLLKP--** **GDAVIPLFVP**

**Chicken 1**  **-STVGKVIKC** **KAAVLWEANK** **PFSLEEVEVA** **PPKAHEVRIK** **IVATGICRSD** **DHVVTGALAM** **-P-FPIILGH** **EAAGVIESVG** **EKVTSLKP--** **GDAVIPLFVP**

**Alligator 1**  **-STAGKVIKC** **KAAITWEIKK** **PFSIEEIEVA** **PPKAHEVRIK** **ILATGICRSD** **DHVTAGLLTM** **-P-LPMILGH** **EAAGVVESTG** **EGVTSLKP--** **GDKVIPLFVP**

**Turtle 1**  **-STSGKIIKC** **KAAIAWELKK** **SLSIEEVEVA** **PPKAHEVRIK** **IFATGICRSD** **EHVMSGALAQ** **-P-FPIILGH** **ESAGVVESVG** **EGVTSVKP--** **GDKVIPLFVP**

**Cobra 1**  **-STAGKVIKC** **KAAIAWELNK** **PLSIEQIEVA** **PPKAHEVRIK** **ILATGVCRSD** **EHVISGAFRM** **-P-LPMVLGH** **EAAGVVESVG** **EGVTCVKP--** **GDKVIPLFAP**

**Uromastyx 1A**  **-GTAGKVIKC** **KAAIAWEIKK** **PLSIEQIEVA** **PPKAHEVRIK** **ILATGICRSD** **DHVISGAFKM** **-P-LPMVLGH** **EAAGVVESVG** **EGVTCVKP--** **GDKVIPLFVP**

**Uromastyx 1B**  **-STAGKVIKC** **KAAVVWEPKK** **PFSIVEIEVA** **PPKAHEVRIK** **ILASGICRSD** **DHVLSGALKV** **-N-FPIILGH** **EAAGVVESVG** **EGVTSMKP--** **GDKVIPIFLP**

**Anole 1A**  **-STAGKVIKC** **KAAIAWEIKK** **PLSIEQIEVS** **PPKAHEVRIK** **ILATGICRSD** **DHVISGAFAM** **-P-LPMVLGH** **EAAGVVESVG** **EGVTCVKP--** **GDKVIPLFVP**

**Anole 1B**  **-STAGKVIKC** **KAAVVWEPKK** **PFSIVEIEVA** **PPKAHEVRIK** **ILASGICRSD** **DHVLSGALIV** **-N-FPIILGH** **EAMGVVESVG** **EGVTSVKP--** **GDKVIPLFLP**

**Anole 1C**  **-STVGKVIKC** **KAAIVFEAKG** **PFKIVEIEVA** **PPKAHEVRIK** **ILGTGICRTD** **EHVQAGAIKV** **-N-YPVIPGH** **EAVGIVESIG** **EGVTCVKP--** **GDKVIPLCLP**

**Anole 1D**  **-STAGKVIKC** **KAAIVFEAKG** **PLKVVEIEVA** **PPKAHEVRIK** **ILATGICRTD** **DHVLNGTIKV** **-N-YPVIPGH** **EAVGAVESIG** **EGVTCVKP--** **GDKIIPLCLP**

**Anole 1E**  **-CTAGKVIKC** **KAAVLFEVKQ** **PLHIVEIEVA** **PPKAHEVRVK** **ILASGICRSD** **DHVLNGTLKV** **-K-FPIIPGH** **EALGVVESIG** **EGVTYVKP--** **GDKVIPLFLP**

**Anole 1F**  **-STAGKVIKC** **KAAIVFEPKK** **PFSIVEIEVA** **PPKAHEARIK** **ILATGICRTD** **DHILSGSIKT** **-T-YPVIPGH** **EAVAVVESIG** **EGVTCVKP--** **GDKVIPLFLP**

**Anole 1G**  **------VIRC** **KAAVVWEPKK** **PFSIVEIEVA** **PPKAHEVRIK** **ILAAGICHSD** **EHVLSDDLKA** **-T-FPVILGH** **EAVGVVESVG** **EGVTSVKP--** **GDKVIPLFIP**

**Anole 1H**  **-STAGKVIKC** **KAAVIWEPKT** **PFSIVEIEVA** **PPKAHEVRIK** **ILASGICRSD** **DHVLSGALKV** **-T-FPIILGH** **EAAGVVESVG** **EGVTSVKT--** **GDKVIPTFVP**

**Rana 1**  **-ATAGKVIKC** **KAAVCWGPKQ** **PLSIEEIEVA** **PPKRHEVRVK** **IVATGICRSD** **DHVISGALSD** **MK-FPVILGH** **EAAGVVESVG** **EGVTKFKP--** **GDKVIPLFVP**

**X.tropicalis 1B**  **-ATAGKVIKC** **KAAVAWGPKQ** **PLTIEDIEVA** **PPKAHEVRVK** **IVATGICRSD** **DHVLSGAISD** **MK-FPAILGH** **EGAGIVESVG** **EGVKNIKP--** **GDKVIPLFVP**

**X.laevis 1B**  **-ATAGKVIKC** **KAAIAWGPKQ** **PLTIEDIEVA** **PPKAHEVRVK** **IVATGICRSD** **DHVLSGAISD** **MK-FPAILGH** **EGAGIVESVG** **EGVKNIKP--** **GDKVIPLFVP**

**X.tropicalis 1A**  **-AAAGKVIKC** **KAAVAWAAKQ** **PFSIEDIEVA** **PPKAHEVRVK** **MVATGICRSD** **DHVLNGSMSF** **PN-FPVILGH** **EGAGIVESIG** **PGVKNIKP--** **GDKVITLFNP**

**X.laevis 1A2**  **-TTAGNVIKC** **KAAVAWAPKQ** **PLSIEDIEVA** **PPKAHEIRVK** **MVATGICGSD** **DTVLSGSFSS** **IK-FPVILGH** **EGAGIVESVG** **TGVKNVKP--** **GDKVIALFQP**

**X.tropicalis 1C**  **-STAGKVIKC** **TAAVVWEPGQ** **PFSIEDIEVA** **PPKAHEVRVK** **IVASGVCHTD** **YHVMSGSVDN** **IK-YPLILGH** **EGAGIVESVG** **EGVKKVKP--** **GDKVIPLSLP**

**X.laevis 1C**  **-STAGKTIEC** **KAAVVWGPDQ** **PFSVETIQVA** **PPKAHEVRVK** **IVASGVCHTD** **YHIMVGAVAN** **VK-YPLILGH** **EGAGIVESVG** **EGVKKVKP--** **GDKVIPLSLP**

**Rana 8**  **-CTAGKDITC** **KAAVAWEPHK** **PLSLETITVA** **PPKAHEVRIK** **ILASGICGSD** **SSVLKEII-P** **SK-FPVILGH** **EAVGVVESIG** **AGVTCVKP--** **GDKVIPLFVP**

**X.tropicalis 8B**  **-DTAGQVIKC** **KAAIAWGDHK** **PLTIEEIEVA** **PPKANEVRIK** **ILASGICGTD** **TAALKGKL-G** **TK-FPAILGH** **EAIGIVESIG** **NGVTTVQP--** **GDKVIPLCMP**

**X.laevis 8B**  **-------IKC** **KAAIAWEEHK** **PLTIEEIEVA** **PPKDHEVRIK** **ILASGVCGTD** **SAALKGKL-G** **TK-FPAILGH** **EAIGVVESIG** **KGVTTVKP--** **GDKVIPLCLP**

**X.laevis 8A**  **-DTTGKVIKC** **KAAIAWELHK** **PLKIEXIEVA** **PPKAHEVRIK** **ILASGVCGSD** **LSVLNDKLGG** **VK-FPNILGH** **EAIGVVESIG** **EGVTVVQP--** **GDKVIPLFVP**

**X.tropicalis 8A**  **-DTTGKVIKC** **KAAIAWGLHK** **PLTIEEIEVA** **PPKAHEVRIK** **ILASGICGSD** **LSVLKDKLSG** **VK-FPTILGH** **EAIGIVESIG** **KDVTVVKP--** **GDKAIPLFVP**

**X.tropicalis 9**  **-STVGKVIHC** **KAAVALEAKK** **PLVIQQIEVA** **PPKAKEVRIK** **IYHSGICHTD** **DHALGGFMAG** **IT-FPVILGH** **EGAGVVESVG** **EGVTTVKP--** **GDHVIAICSP**

**X.laevis 9**  **-STVGKVIHC** **KAAVALEAKK** **PLVIQQIEVA** **PPKAKEIRIK** **IYHSGICHTD** **DHALGGFMAG** **IK-FPIILGH** **EGAGVVESVG** **EGVTSVEP--** **GDHVIAICSP**

**X.tropicalis 10B** **-ESAGQVIKC** **KAAVTWEKDA** **PFSIEEIEVA** **PPKAHEVRIK** **LIATGICRSD** **DHSLEGKFAA** **VK-FPVILGH** **EGVGIVESIG** **DGVKDIKP--** **GDKVIPLVAP**

**X.laevis 10B**  **-ESAGQVIKC** **KAAVTWGKNA** **PFSIEEIEVA** **PPKAHEVRIK** **MIATGICRSD** **HHSIEGKFSS** **VK-FPVIVGH** **EGVGIVESIG** **EGVKDIKP--** **GDKVIPLVTP**

**X.tropicalis 10A** **-DTAGKVIKC** **KAAVIWEKSA** **PFSIEEVEVA** **PPKANEVRIK** **IVATGICRSD** **DHAIEGKLST** **VK-FPVILGH** **EGVGIVESTG** **ESVKHIKP--** **GDRVIPLFVP**

**X.laevis 10A**  **-DTAGKVIKC** **KAAVTWEKGA** **PFSIEEVEVA** **PPKAQEVRIK** **IIATGICRSD** **DHAVEGKLSQ** **VK-FPIILGH** **EAVGIVESTG** **ESVKNLKP--** **GDRVIPLFAP**

**Human 5**  **-STTGQVIRC** **KAAILWKPGA** **PFSIEEVEVA** **PPKAKEVRIK** **VVATGLCGTE** **MKVLGSKHLD** **LL-YPTILGH** **EGAGIVESIG** **EGVSTVKP--** **GDKVITLFLP**

**Bovine 5**  **-STTGKVIRC** **KAAIVWKPGG** **SFSIEEVEVA** **PPKAKEVRIK** **MVATALCGTE** **MKMLKDKNLQ** **HQHYPIIMGH** **EGAGIVESVG** **EGVSTVKA--** **GDKVIALFLP**

**Rabbit 5**  **-STAGKVIRC** **RAAVLWKSGA** **PFSIEEVEVA** **PPKEKEVRIK** **IVATGLCGTE** **MKMLARTDLP** **GL-YPIILGH** **EGAGIVESVG** **GGVNTVKP-V** **GDKVIILFLP**

**Rat 5**  **-GTQGKVIRC** **KATVLWKPGA** **PLAIEEIEVA** **PPKAKEVRIK** **MVATGVCGTD** **IKHLDTQELS** **KF-CPMIMGH** **EGVGIVESVG** **EGVSSVRT--** **GDKVILLCIP**

**Deer mouse 5**  **-STAGKVIRC** **KAAVLWKPGA** **PLTMEEIDVA** **PPKGKEVRVK** **MVAAGICGTD** **IKSLDNKKLA** **PF-CPIIMGH** **EGTGIVESVG** **EGVSTVKT--** **GDKVIILCLP**

**X.tropicalis 7**  **-DTAGKIVKC** **KAAVAWEIGK** **PLTIEEIEVE** **VPKASEVRIK** **MVATGICRTD** **DHVLKGALKG** **ID-FPVILGH** **EGAGIIESVG** **EGVTGLKP--** **GDKVIPLCIP**

**Turtle 7**  **-GTAGKVIKC** **KAAIAWEANK** **PVSVEEVEVA** **PPKAREVRVK** **IVATGICRTD** **DHVLKGSLPN** **IN-YPVIPGH** **EGAGIVESIG** **EGVTCVKP--** **GDKVIPLCLP**

**Chicken 7**  **-ATSGKVIRC** **RAAVAWAAGK** **SLSVEEIEVA** **PPKAREVRVK** **MVATGICHTD** **EHVLEGNFPD** **VD-FPVILGH** **EGAGIVESIG** **EGVTSVKP--** **GDKVILFPLP**

**Zebra finch 7**  **-ATAGKVIRC** **RAAVAWAPGK** **PLSVEEVEVA** **PPKAGEVRIK** **IVATSICHTD** **DHVLKGSLQK** **VE-FPVIPGH** **EGAGIVESIG** **QGVTSVKP--** **GDKVIPLCLP**

**Pigeon 7**  **-ATSGKVIRC** **RAAVAWAVGK** **PLSVEEVEVA** **PPKAGEVRIK** **IVATGICRTD** **DHVVKGCIAN** **VE-FPVIPGH** **EGAGIVESIG** **EGVTSVKP--** **GDKVIPLCIP**

**Anole 7A**  **-ATVGKVIKC** **KAAIIWEVGK** **TPSIEEVEVA** **PPKSHEVRVK** **IVATGICRTD** **YHVLKGFFPK** **LD-YPVIAGH** **EGAGIVESIG** **PGVTCVKP--** **GDKVIPLCLP**

**Anole 7B**  **-ETTGKIIKC** **KAAVAWEIGK** **PLSIEEIEVA** **PPKSHEVRVK** **IIATGICGTD** **DHVLSGSFPK** **VN-YPVIPGH** **EGAGIVESVG** **EGVTGLNP--** **GDKVIPLCLP**

**Anole 7C**  **-ATAGKVIKC** **KAAIIWEVGK** **MPSIEEVEVA** **PPKNHEIRIK** **IVATSICRTD** **DNVLKGIFPD** **LE-YPVIAGH** **EGAGIVESVG** **PGVTYVKP--** **GDKVIPLCVP**

**Rat 6A**  **-DTLGKTITC** **RAAIAWAENS** **PLSIEEVQVE** **PPKSGEVRIK** **MISSGICGSD** **DHMLKGELLA** **-N-FPLIPGH** **EGAGIVESVG** **DGVCSVKP--** **GDKVLTLIIP**

**Mouse 6A**  **-NTLGKTLTC** **RAAIAWAKNS** **PLSIEEVQVE** **PPKSGEVRIK** **MISSGICGSD** **DHVLKGELVV** **-N-FPLIPGH** **EGAGIVESVG** **DGVCSMKP--** **GDKVLILIIP**

**Rat 6B**  **------VITC** **RAAVAWTTNA** **PLSVEEVTVD** **PPKACEVRVK** **IISSGICGTD** **NHILEGKMET** **-P-FPVILGH** **EGAGIVESIG** **QGVTTVKP--** **GDKVLMFPLP**

**Mouse 6B**  **------VIRC** **RAAVAWTTNA** **PLSIEEVEVD** **PPKAGEVRVK** **IIASGICGTD** **NHTLEGKMKT** **-P-FPVILGH** **EGAGVVESVG** **PGVTTVKP--** **GDKVLMFPLP**

**Bovine 6**  **-DTLGKTITC** **LAAIAWKTNS** **SLSLEEVQVE** **PPKAGEVRIK** **MISTGICGSD** **DHAIKGIMPL** **-K-YPFIPGH** **EGAGLVESIG** **EGVSSVKP--** **GDKVLTLIVP**

**Dog 6**  **-SLCPQTITC** **RAAIAWAANS** **TLSIEEVQVE** **PPKAGEVRIK** **LASTGICGTD** **DHAIKGLLSA** **-I-FPFIPGH** **EGAGIVESIG** **KGVTSVKP--** **GDKVLTLIIP**

**Panda 6**  **LSLCQQTITC** **RAAVAWAANS** **TLSIEEVQVE** **PPKAGEVRIK** **LTSTGICGSD** **DHAIKGLLSA** **-V-FPYIPGH** **EGAGIVESIG** **KGVSSVKPDI** **GDKVLTLIIP**

**Horse 6**  **-----QVITC** **WAAIAWTANA** **PLSIEEVEVD** **PPKAGEVRIK** **ILSSGLCGTD** **LHILEGKLRV** **-P-FPMILGH** **EGAGIVESTG** **DGVTSVKA--** **GDKVLMFPLP**

110 120 130 140 150 160 170 180 190 200

....|....| ....|....| ....|....| ....|....| ....|....| ....|....| ....|....| ....|....| ....|....| ....|....|

**Human 3**  **QCGECKFCLN** **PKTNLCQKIR** **----VTQGKG** **LMPDGTSRFT** **CKGKTILHYM** **GTSTFSEYTV** **VADISVAKID** **PLAPLDKVCL** **LGCGISTGYG** **AAVNTAKLEP**

**Rat 3**  **QCGECKFCLN** **PKTNLCQKIR** **----VTQGKG** **LMPDGTSRFT** **CKGKPILHFM** **GTSTFSEYTV** **VADISVAKID** **PSAPLDKVCL** **LGCGISTGYG** **AAVNTAKVEP**

**Mouse 3**  **QCGECKFCLN** **PKTNLCQKIR** **----VTQGKG** **LMPDGTSRFT** **CKGKSVFHFM** **GTSTFSEYTV** **VADISVAKID** **PSAPLDKVCL** **LGCGISTGYG** **AAVNTAKVEP**

**Rabbit 3**  **QCGECKFCLN** **PKTNLCQKIR** **----VTQGKG** **LMPDGTSRFT** **CKGKTILHYM** **GTSTFSEYTV** **VADISVAKID** **PSAPLDKVCL** **LGCGISTGYG** **AALNTAKVEP**

**Chicken 3**  **QCGECKYCKN** **PKTNLCQKIR** **----VTQGKG** **LMPDGTIRFT** **CKGKQIYHFM** **GTSTFSEYTV** **VADISVAKID** **PAAPFDKVCL** **LGCGVSTGYG** **AAVNTAKVEP**

**Turtle 3**  **QCGECKFCLN** **PKTNLCQKIR** **----VTQGKG** **LMPDGTSRFT** **CKGKQIYHFM** **GTSTFSEYTV** **VADISLAKID** **AAAPLDKVCL** **LGCGISTGYG** **AVINTAKVEP**

**Uromastyx 3**  **QCGECKFCLN** **PKTNLCQKIR** **----VTQGKG** **VMPDGTSRFT** **CKGKQVLHFM** **GTSTFSEYTV** **VADISLTKIN** **ASAPLDKVCL** **LGCGVSTGYG** **AALNTAKVEP**

**X.tropicalis 3**  **QCGECKFCLN** **PKTNLCQKIR** **----ITQGKG** **FMPDGSSRFT** **CKGQQIFHFM** **GTSTFSEYTV** **VADISVAKID** **DSAPLDKVCL** **LGCGISTGYG** **AVINTAKVEP**

**X.laevis 3**  **QCGECKFCLN** **PKTNLCQKIR** **----ITQGKG** **FMPDGTSRFT** **CKGQQIFHFM** **GTSTFSEYTV** **VADISVAKIE** **DSAPLDKVCL** **LGCGISTGYG** **AVINTAKVEP**

**Anole 3**  **QCGECKFCLN** **PKTNLCQKIR** **----VTQGKG** **VMPDGTSCFT** **CKGKQVLHFM** **GTSTFSEYTV** **VADISLAKID** **ASAPLDKVCL** **LGCGVSTGYG** **AALNTAKVEP**

**Marmoset 2**  **HCRKCKFCLS** **PLTNFCGKIS** **NLKNPATHQQ** **LMEDKTSRFT** **CKGKPIYHFL** **GISTFSQYTV** **VSDINLVKID** **DDANLERVCL** **LGCGFSTGYG** **AAINNAKVTP**

**Bovine 2**  **QCGKCKFCLS** **PRTNFCGKLK** **HFKNPMGDQK** **LMEDGTSRFT** **CKGKPIYHFM** **GTSTFSQYTV** **VSDVNLAKLE** **DDANLERVCL** **LGCAFSTGYG** **AVINNAKVTP**

**Human 2**  **LCRKCKFCLS** **PLTNLCGKIS** **NLKSPASDQQ** **LMEDKTSRFT** **CKGKPVYHFF** **GTSTFSQYTV** **VSDINLAKID** **DDANLERVCL** **LGCGFSTGYG** **AAINNAKVTP**

**Rat 2**  **QCKKCKLCLS** **PLTNLCGKLR** **NFKYPTIDQE** **LMEDRTSRFT** **SKERSIYHFM** **GVSSFSQYTV** **VSEANLARVD** **DEANLERVCL** **IGCGFTSGYG** **AAINTAKVTP**

**Mouse 2**  **QCKRCKLCLS** **PLTNLCGKLR** **NFKYPTIDQE** **LMEDRTSRFT** **CKGRSIYHFM** **GVSSFSQYTV** **VSEANLARVD** **DEANLERVCL** **IGCGFSSGYG** **AAINTAKVTP**

**Rabbit 2A**  **HCKKCKFCLS** **PLTNFCEKFC** **KGKNPLIEQE** **LMEDKTSRFT** **CKGKSIYHFF** **GISAFSQYTV** **VKDVNLAKID** **DDANLERVCL** **IGCGFSTGYG** **AAINTAKVTP**

**Rabbit 2B**  **HCRKCKFCQS** **PLTNFCTKFS** **EHKNPIIEQE** **LMDDKTSRFT** **CKGKSIYHFL** **GISAFSQYTV** **VKDINLAKID** **DDANLKRVCL** **IGCGFSTGYG** **AAINDAKVTP**

**Ostrich 2**  **QCGHCKFCLN** **PKTNLCEKIS** **KIKTPISDQE** **VMSDGTSRFT** **CKGKPIYHFM** **GTSTFSEYTV** **VSESSLAKID** **AAAPLDKVCL** **IGCGFSTGYG** **AAINTAQVEP**

**Turtle 2**  **QCGQCKFCLS** **PKTNLCEKIS** **KIKTAISDQD** **LMPDGTSRFT** **CKGKQIYHFM** **GTSTFSEYTV** **VAETSLVKID** **DAAPLEKVCL** **IGCGFSTGYG** **AAINTAKVEP**

**X.tropicalis 2**  **QCGECKFCLN** **PKTNLCEKIS** **KIKTAISDQD** **LMADNTSRFT** **CKGKQIYHFM** **GTSTFSEYTV** **CAETSVAKID** **DAAPLEKVCL** **IGCGFSTGYG** **AAINTAKVEP**

**Human 4**  **QCRECNACRN** **PDGNLCIRSD** **----I-TGRG** **VLADGTTRFT** **CKGKPVHHFM** **NTSTFTEYTV** **VDESSVAKID** **DAAPPEKVCL** **IGCGFSTGYG** **AAVKTGKVKP**

**Rat 4**  **QCRECNPCRN** **PEGNLCIRSD** **----L-TGRG** **VLADGTTRFT** **CKGKPVQHFM** **NTSTFTEYTV** **LDESSVAKID** **AEAPPEKACL** **IGCGFSTGYG** **AAVKTAKVSP**

**Mouse 4**  **QCRECNACLN** **PEGNLCIRSD** **----L-TGCG** **VLADGTTRFT** **CKGKPVQHFM** **NTSTFTEYTV** **LDESSVAKVD** **GAAPPEKACL** **IGCGFSTGYG** **AAVKTAKVTP**

**Human 1B1**  **QCGKCRVCKN** **PESNYCLKND** **----LGNPRG** **TLQDGTRRFT** **CRGKPIHHFL** **GTSTFSQYTV** **VDENAVAKID** **AASPLEKVCL** **IGCGFSTGYG** **SAVNVAKVTP**

**Rat 1**  **QCGKCRICKH** **PESNLCCQTK** **---NLTQPKG** **ALLDGTSRFS** **CRGKPIHHFI** **STSTFSQYTV** **VDDIAVAKID** **AAAPLDKVCL** **IGCGFSTGYG** **SAVQVAKVTP**

**Mouse 1**  **QCGECRICKH** **PESNFCSRSD** **----LLMPRG** **TLREGTSRFS** **CKGKQIHNFI** **STSTFSQYTV** **VDDIAVAKID** **GASPLDKVCL** **IGCGFSTGYG** **SAVKVAKVTP**

**Rabbit 1**  **QCGKCRICKH** **PESNFCLIND** **----LGKPKG** **MLLDGTSRFT** **CKGKPIHHFI** **GTSTFSQYTV** **VDEIAVAKID** **AAAPLEKVCL** **IGCGFSTGYG** **SAVKVAKVTP**

**Ostrich 1**  **QCGECSVCLS** **TKGNLCRKND** **----IGPASA** **LMPDGTSRFT** **CKGKAIHHFA** **GTSTFTEYTV** **LHETAVAKID** **AAAPLEKVCL** **IGCGFSTGYG** **AALQTAKVEP**

**Quail 1**  **QCGECRSCLS** **TKGNLCIKND** **---LSSSPTG** **LMADGTTRFT** **CKGKAIHHFI** **GTSTFTEYTV** **VHETAAAKID** **SAAPLEKVCL** **IGCGFSTGYG** **AVLQTAKVEP**

**Chicken 1**  **QCGECRSCLS** **TKGNLCIKND** **---LSSSPTG** **LMADGTTRFT** **CKGKAIHHFV** **GTSTFTEYTV** **VHETAAAKID** **SAAPLEKVCL** **IGCGFSTGYG** **AVLQTAKVEA**

**Alligator 1**  **QCGECMPCLK** **SNGNLCIRND** **----LGSPSG** **LMADGTSRFT** **CKGKDIHHFI** **GTSTFTEYTV** **VHETAVARID** **AAAPLEKVCL** **IGCGFSTGYG** **AAVKDAKVEP**

**Turtle 1**  **QCGECSSCIS** **PKGNLCTKSD** **----LTTSRG** **LMCDGTSRFT** **CKGKSIHHFI** **GTSTFTEYTV** **VHETAVAKID** **ELAPPEKVCL** **IGCGFSTGYG** **AAINTAKVEG**

**Cobra 1**  **QCGKCRACQS** **PKGNLCTSND** **----LNSGSG** **LMPDGTSRFT** **CKGKSIHHFI** **STSTFTEYTV** **VHENSVVKID** **PSAPLEKVCL** **IGCGFSTGYG** **AAMQTAKVEP**

**Uromastyx 1A**  **QCGKCSSCRS** **TRGNLCTSND** **----LSAATG** **LMPDGTSRFT** **CKGKSLHHFI** **STSSFTEYTV** **VHENSVVKID** **AAAPLEKVCL** **IGCGFSTGYG** **AAVETAKVEP**

**Uromastyx 1B**  **QCGECNSCRH** **PRGNVCKKSE** **----LGPFTG** **LLYDGTSRFT** **YQGKPVYHFV** **RTGTFTEYTV** **APEDSVVKID** **ASAPLEKVCL** **IGCGFSTGYG** **AAINSAKVQP**

**Anole 1A**  **QCGKCSSCQS** **TKGNLCTAND** **----LSSGQG** **LMPDGTCRFT** **CKGKSLHHFI** **STSTFTEYTV** **VHENAVVKID** **DAAPLEKVCL** **VGCGFSTGYG** **AAVQTAKVEP**

**Anole 1B**  **QCEKCSFCIH** **PKGNLCEKAD** **----FGIPTG** **LMYDGTSRFT** **YKGQPIYKFV** **STGTFTEYTV** **VHEDAVAKID** **AAAPPEKVCL** **IGCGFSTGYG** **AAVNSAKVEP**

**Anole 1C**  **QCGRCPPCKN** **PEGNMCQKSD** **----FLTAKG** **VMYDGTSRFS** **YKGKPVHNFV** **NTSTFTEYTV** **VHEDAVVKID** **PAAPIEKACL** **MGCGFSTGYG** **AATKTAKVTP**

**Anole 1D**  **QCGKCRPCKN** **PEGNLCEQSD** **----FFKNTG** **LMYDGTSRFS** **YKGKPIHHFA** **NTSTFTEYTV** **VHEDSVAKID** **PAAPPEKACL** **IGCGFSTGYG** **AATKTAKVTP**

**Anole 1E**  **QCGKCRACKN** **PQRNLCEKSD** **----TFKGTG** **LMYDGTSRFS** **YKGKPVHHFA** **NTSTFTEYTV** **VHEDAVAKVD** **PAAPPEKACL** **IGCGFSTGYG** **AATKTAKVTP**

**Anole 1F**  **QCGKCRTCKN** **PEGNLCEKND** **----LYEFKG** **LMYDGTTRFS** **YKGKPVHNFA** **STSTFTEYTV** **VHEDSLAKID** **PAAPPEKACL** **IGCGFSTGYG** **AAIRSGKVTP**

**Anole 1G**  **QCEECRPCIH** **PRGNLCKLNG** **----IETPLG** **LMPDGTSRFS** **YKGELVHNFV** **STSTFTEYTV** **VHESSVAKID** **ASAPPEKACL** **IGCGFSTGYG** **AAINSAKVEP**

**Anole 1H**  **QCGECSPCIH** **PRGNLCKLND** **----FGTFVG** **LLPDGTSRFS** **YKGELIHSFV** **STSTFTEYTV** **VHESSVAKID** **AAAPPEKVCL** **IGCGFSAGYG** **AAINSAKVEP**

**Rana 1**  **QCGECRCCKN** **PESNLCYKND** **----IGKYDG** **VLLDKTSRFT** **CKGKSIHNFI** **STSTFTEYTV** **LDEIAVAKIH** **EDAPLEKVCL** **IGCGFSTGYG** **SAVNTGKVKP**

**X.tropicalis 1B**  **QCGECRCCTN** **VRSNLCDKHD** **----IGPYRG** **LMLDNTNRFT** **CKGNPVYTFL** **STSTFTEYTV** **LDEICVAKID** **DNAPLDKVCL** **IGCGFSTGYG** **SAVKIAKVEQ**

**X.laevis 1B**  **QCGECRSCTN** **SRSNLCDKHD** **----IGPYIG** **LLLDKTSRIT** **CKGKPVYNFL** **STSTFTEYTV** **LDEICVAKID** **DNAPLDKVCL** **IGCGFSTGYG** **SAVKTAKVEP**

**X.tropicalis 1A**  **QCRECKNCLD** **PKSNVCIKSD** **----IGKLTG** **LMLDNTSRFT** **CKGKQIHHFV** **HTSTFTEYTV** **LDEMAVAKIH** **NDAPLDKVCL** **IACGFSTGYG** **SALNTAKVEP**

**X.laevis 1A2**  **QCGECQNCLN** **PKSNMCFKSD** **----LGKYTG** **MMLDNTSRFT** **CKGKLIHHFM** **HTSTFTEYTV** **LDEIAVAKIQ** **DDAPLDKVCL** **ISCGFSAGYG** **SVLNTAQVKQ**

**X.tropicalis 1C**  **QCGKCLLCLN** **PKSNICEKSD** **----FGKYSG** **LMLDNTSRFT** **CKGKLIYHFV** **RTSTFTEYTV** **LDEISVAKID** **DSAPLDKVCL** **ISCGFSTGYG** **SAVKIAKVEP**

**X.laevis 1C**  **QCGECQNCLN** **PKSNICEKSD** **----FGKFSG** **LMLDNTSRFT** **CKGKLIHNFV** **STSTFTEYTV** **LDEICVAKID** **DNAPLDKVCL** **IGCAFSTGYG** **SALNTAKVEP**

**Rana 8**  **QCGSCRACKS** **SNSNFCEKND** **----MGAKTG** **LMADMTSRFT** **CRGKPIYNLV** **GTSTFTEYTV** **VADIAVAKID** **PKAPLE-SCL** **IGCGFATGYG** **AAVNTAKVTP**

**X.tropicalis 8B**  **QCGQCRACKS** **PNANLCDKND** **----FTNNTG** **LMQDKTSRFT** **CKGKQVYHFV** **GTSTFTEYTV** **VSEISVAKVD** **PAAPLE-VCI** **VGCGFATGYG** **AAVN-SKIAP**

**X.laevis 8B**  **QCRQCRACKN** **PNANLCVKND** **----FGSNSG** **LMHDNTSRFT** **CKGKQIYHFL** **FTSTFTEYSV** **IPEICIAKVN** **PAAPDE-VCI** **IGCGFATGYG** **AAVN-AKIPP**

**X.laevis 8A**  **QCGQCRACKT** **PNCNLCDKND** **----FIAKTG** **LMQDNTSRFT** **CKGKQIYHYM** **CTSTFTEYTV** **VPDICVAKVD** **PAAPVE-GCL** **IGCGFATGYG** **AAVNTAKVTP**

**X.tropicalis 8A**  **QCGQCRACKT** **PNCNVCEKND** **----FTTKKG** **LMQDNTSRFT** **CKGEQVYHFM** **STSTFTEYTV** **VPDICVAKVD** **PAAPVE-GCL** **IGCGFATGYG** **AAVNTAKVTP**

**X.tropicalis 9**  **MCMKCPSCLH** **PDSNFCVKND** **----VGKHVG** **LMLDKTSRFS** **IKGKLIHNFM** **STSTFCEYTV** **VDEFACVKID** **PKAPLHEVCL** **IGCGFSTGYG** **SVLNTAKVQP**

**X.laevis 9**  **MCMKCPSCLH** **PDSNFCVQND** **----VGKNVG** **MMLDKTSRFS** **INGKMIHNFM** **STSTFCEYTV** **LDEFACVKID** **CKAPLYEVCL** **IGCGFSTGYG** **SVLNTAKVQQ**

**X.tropicalis 10B** **QCGKCQCCKD** **PRTNRCLT-R** **----LKRQFG** **LMSDGTSRFT** **CRGKQIYHFM** **NTSTFTEYTV** **VEEMAVAKID** **DNATMDSVCL** **IGCGFSTGYG** **SALNTAKVHP**

**X.laevis 10B**  **QCGQCQCCKD** **PRANRCLTIK** **----LERQYG** **LMADGTSRFT** **CRGKQIYHFL** **NTSTFTEYTV** **VEEIAVAKID** **DTATMDSVCL** **IGCAFSTGYG** **SVINTAKVHP**

**X.tropicalis 10A** **QCGQCICCKD** **PRSNMCIAGK** **----MKKAVG** **LMSDGTSRFT** **CKGKQIYHFM** **NTSTFTEYTV** **AEEMSVAKID** **SGASLDNTCL** **IGCGFSTGYG** **SALNSAKVHP**

**X.laevis 10A**  **QCEQCMCCKD** **PRSNICIATK** **----LTKSVG** **VMSDGTSRFT** **CKGKQIYHFL** **NTSTFTEYTV** **VDEMAVTKID** **GGASLENTCL** **IGCGFSTGYG** **SALNTAKVHP**

**Human 5**  **QCGECTSCLN** **SEGNFCIQFK** **----QSKTQ-** **LMSDGTSRFT** **CKGKSIYHFG** **NTSTFCEYTV** **IKEISVAKID** **AVAPLEKVCL** **ISCGFSTGFG** **AAINTAKVTP**

**Bovine 5**  **QCGECTSCLN** **SADNFCIKLK** **----QAETH-** **LMSDGTSRFT** **CKGKPVYHFG** **NTSTFSEYTV** **MDEISVAKID** **AAAPLEKVFL** **VSCGFSTGYG** **GAINTAKVTP**

**Rabbit 5**  **QCGECSSCLN** **SGENYCIKLK** **----QSKTA-** **LMSDGTSRFT** **CKGKSVKQFA** **ATSTFTEYTV** **VREDSIAKID** **ASAPLEKVCL** **ISCGFSTGFG** **AAINTAKVTP**

**Rat 5**  **QCGECKTCLN** **SKNNICTEIR** **----LSKTH-** **LASEGTSRIT** **CKGKLVHQYI** **ALGSFSEYTV** **LKEISVAKID** **EGAPLEKVCI** **IGCGFATGYG** **AAINSAKVTP**

**Deer mouse 5**  **QCGECNTCLN** **SKNNICKEVR** **----LSGTH-** **LTSEGNSRIT** **CKGKTTYQYI** **TTGTFSEYIV** **IKEISVAKVD** **EDALLEKACI** **IGCGFATGFG** **AAINSAKVSP**

**X.tropicalis 7**  **QCGKCSSCLN** **PNTNCCLKTH** **----LSESQN** **VMPDKTSRFL** **CKGKAAYHFL** **WTSTFSEYTV** **VPVDAVAKID** **DRVPMDKACL** **FGCGFPTGYG** **AVVNTAKVEP**

**Turtle 7**  **QCGECSSCLN** **PDTNCCLKTH** **----LYESQN** **LMPDNTSRFT** **CKGQRIYHFL** **WISTFSEYTV** **MPDSAIAKID** **DDAPLDKVCL** **FGCGFSTGYG** **AAINTAKVKP**

**Chicken 7**  **QCGECSFCLN** **PDSNYCLKSH** **----LTEPQN** **LMPDKTTRFT** **CKGKQIQHFL** **WNSTFAEYTV** **VPEYTLAKID** **AAAPLDKVCV** **FACGFSTGYG** **AAVNTAKVKL**

**Zebra finch 7**  **QCGECSFCQN** **PESNYCQKTH** **----FSEPQN** **LLPDKTSRFT** **CKGKQIHHYL** **WISTFAEYTV** **VPEYAVAKID** **AAAPLDKVCL** **IACGFSTGYG** **AAINTAKVKP**

**Pigeon 7**  **NCGECTFCLN** **PEASYCVKSH** **----FSEPQN** **LMPDKTSRFT** **CKGKQIHHFM** **WVSTFAEYTV** **APETAVAKID** **SAAPLDKVCL** **LGCGFSTGYG** **AAINTAKVKP**

**Anole 7A**  **QCGECSCCRS** **PKDNCCLKTH** **---FCERPQN** **LMPDQTSRFT** **CKGKRIHHLM** **WVSTFSEYTV** **MPDASIVKID** **NNAPLDRVCL** **LGCGFPTGYG** **AAINTAEVAP**

**Anole 7B**  **QCGECSSCLK** **SNTNCCLKTH** **----LNEPQN** **LMPDKTSRFT** **CKGKLIHHFL** **WISTFSEYTV** **MPDSTVVKID** **AAAPLDKVCL** **FGCGFSTGYG** **AALNTAQVMP**

**Anole 7C**  **QCGECACCLS** **PKANCCLKSH** **---ICKPLQN** **LMPDKTSRFT** **CRGKQIHHFL** **WISTFSEYTV** **VPDVSIAKIS** **NDAPLDKVCL** **FGCGFSTGYG** **AAINNAKVEP**

**Rat 6A**  **QCRECDSCLH** **LKGNFCEKQD** **----VLPCSG** **VMLDGTSRFS** **CRGRKIYHSF** **RTSSFTEYTV** **VPEIAVVKID** **DAAPMDKVCL** **ISCGFPTGYG** **AAVNSAKVTP**

**Mouse 6A**  **QCRECDACLH** **PKGNFCYKQD** **----VLPCSG** **VMLDGTSRFS** **CRGQKIYHSF** **RTSSFTEYTV** **VPEIAAVKID** **DAAPMDKVCL** **ISCGFPTGYG** **GAVNSAKVTP**

**Rat 6B**  **ECRECIYCLH** **PKGNFCKKQN** **----ILSPTG** **LMLDGTSRFT** **CRGKKIYHLQ** **GTSTFTEYTV** **VDEIAVAKIN** **DRAPMDTVCI** **ISCEVSTGFG** **AVFNTAQVTP**

**Mouse 6B**  **ECRECFYCLY** **PKGNFCEKEN** **----ILSPTG** **LMLDGTSRFT** **CRGKKIYNIM** **GTSTFTEYTV** **VHEIAVAKIE** **AAAPMDTVCI** **MSCAVPTGFG** **AVFNTAQVTP**

**Bovine 6 QCRECSACLN** **PKGNFCEKQD** **----ILPSSG** **LMLDGTSRFT** **CKGEKIYHSF** **RTSTFSEYTV** **VPEISVVKID** **AAAPMDKICV** **ISCEVPTGYG** **AAVHSAKVTP**

**Dog 6**  **QCRECSSCLH** **PKGNFCEKQD** **----VLPSSG** **LMLDGTSRFT** **CKGKKIYHSF** **RTSTFTEYTV** **VPEIAVAKID** **DAAPMDKVSL** **ISCEVPTGYG** **AAVHSAKVTH**

**Panda 6**  **QCRECSSCLH** **PKGNFCEKQD** **----VLSSSG** **LMLDGTSRFT** **CKGKKIHHSF** **RTSTFTEYTV** **VLEIAVAKID** **DAAPMDKVSA** **ISCEVPTGYG** **AAVHSAKVTR**

**Horse 6**  **ECRECSSCLH** **PKGNLCLKEN** **----VLSPNG** **LMLDGTSRFT** **CRGKKIYHLY** **GTSTFAEYTV** **VHEIAVGKID** **AAAPMDKVCI** **MSCEVPTGFG** **AVFNTAKVTP**

210 220 230 240 250 260 270 280 290 300

....|....| ....|....| ....|....| ....|....| ....|....| ....|....| ....|....| ....|....| ....|....| ....|....|

**Human 3**  **GSVCAVFGLG** **GVGLAVIMGC** **KVAGASRIIG** **VDINKDKFAR** **AKEFGATECI** **NPQDFSKPIQ** **EVLIEMTDG-** **GVDYSFECIG** **NVKVMRAALE** **ACHKGWGVSV**

**Rat 3**  **GSTCAVFGLG** **GVGLAVIMGC** **KVAGASRIIG** **IDINKDKFAK** **AKEFGATECI** **NPQDFSKSIQ** **EVLIEMTDG-** **GVDFSFECIG** **NVKVMRSALE** **AAHKGWGVSV**

**Mouse 3**  **GSTCAVFGLG** **GVGLAVIMGC** **KVAGASRIIG** **IDINKDKFAK** **AKEFGASECI** **SPQDFSKSIQ** **EVLVEMTDG-** **GVDYSFECIG** **NVKVMRSALE** **AAHKGWGVSV**

**Rabbit 3**  **GSTCAVFGLG** **GVGLAAIMGC** **KAAGASRIIA** **VDINKDKFAR** **AKEFGATECI** **NPQDFSKPIQ** **EVLVEKTDG-** **GVDYSFECIG** **NVKVMRAALE** **ACHKGWGVSV**

**Chicken 3**  **GSTCAVFGLG** **GVGLATVMGC** **KAAGASRIIG** **IDINKNTYAK** **AKEFGAAECI** **SPQDFEKPIQ** **EVLVEMTDG-** **GVDYSFECIG** **NVGVMRAALE** **ACHKGWGVSV**

**Turtle 3**  **GSTCAIFGLG** **GVGLAVIMGC** **KVAGASRIIG** **VDLNKDKFAK** **AKEFGATECI** **NPQDFKKPIQ** **EVLVELTEG- GVDYSFECIG** **NVGVMRAALE** **ACHKGWGVSV**

**Uromastyx 3**  **GSTCAVFGLG** **GVGLAVIMGC** **KVAGASRIIG** **IDLNKDKFAK** **AKEFGATECI** **SPADFKKPIQ** **EVLIEMTDG-** **GVDYSFECIG** **NVGVMRAALE** **ACHKGWGVSV**

**X.tropicalis 3**  **GSTCAVFGLG** **GVGLAVIMGC** **KVAGATRIIG** **IDLNKDKFVK** **ATEFGATDCL** **NPADFKKPIQ** **DVLIEMTDG-** **GVDYSFECIG** **NVGVMRAALE** **ACHKGWGTSV**

**X.laevis 3**  **GSTCAVFGLG** **GVGLAVIMGC** **KVAGATRIIG** **IDLNKDKFAK** **ATEFGATECL** **NPADFNKPIQ** **DVLIDMTDG-** **GVDYSFECIG** **NVRVMRSALE** **ACHKGWGTSV**

**Anole 3**  **GSTCAVFGLG** **GVGLAVIMGC** **KVAGASRIIG** **IDLNKDKFTK** **AKEFGATECI** **SPEDSKKPIQ** **EVLVEMTDG-** **GVDYSFECIG** **NVHVMRAALE** **ACHKGWGVSV**

**Marmoset 2**  **GSTCAVFGLG** **GVGLSAVIGC** **KMAGASRIIG** **VDINSEKFAK** **AKALGATECL** **NPRDLHKPVQ** **EVITELTKG-** **GVDFALDCAG** **GSESMKAALD** **CTTAGWGSCT**

**Bovine 2 GSTCAIFGLG** **GVGLSAVMGC** **KASGASRIIV** **VDINSEKFTK** **AKALGATDCL** **NPKDLDKPIQ** **EVIVEMTNG-** **GVDFAFECVG** **GAKIMRAALD** **SVTVGWGVCT**

**Human 2**  **GSTCAVFGLG** **GVGLSAVMGC** **KAAGASRIIG** **IDINSEKFVK** **AKALGATDCL** **NPRDLHKPIQ** **EVIIELTKG-** **GVDFALDCAG** **GSETMKAALD** **CTTAGWGSCT**

**Rat 2**  **GSACAVFGLG** **CVGLSAVIGC** **KIAGASRIIA** **IDINSEKFPK** **AKALGATDCL** **NPRDLDKPVQ** **DVITELTGG-** **GVDFSLDCAG** **TAQTLKAAVD** **CTVVGWGSCT**

**Mouse 2**  **GSTCAVFGLG** **CVGLSAIIGC** **KIAGASRIIA** **IDINGEKFPK** **AKALGATDCL** **NPRELDKPVQ** **DVITELTAG-** **GVDYSLDCAG** **TAQTLKAAVD** **CTVLGWGSCT**

**Rabbit 2A**  **GSTCAVFGLG** **GVGLSAIMGC** **KTAGASRIIA** **IDINSDKFAK** **AKALGATDCL** **NPRELNKPVQ** **DVIVEMTNG-** **GVDFAIDCAG** **GSEVMKATVD** **CATVGWGSCT**

**Rabbit 2B**  **GSTCAVFGLG** **GVGLSAVIGC** **KTAGASRIIA** **VDINSDKFAK** **AKALGATDCL** **NPRELNKPVQ** **DVIVEMTNG-** **GVDFAIDCAG** **GSEVMKATVD** **CTTVGWGSCT**

**Ostrich 2**  **GSTCAVFGLG** **GVGLSAVMGC** **KAAGASKIFG** **IDINKDKFPL** **AKKLGATDCL** **NPQDIRKPVQ** **EIIAEMTNG-** **GVDFAIECIG** **NPDVMKAAFE** **STTVGWGTCV**

**Turtle 2**  **GSTCAVFGLG** **GVGLSAVMGC** **KVSGASRIFA** **IDINKDKFSL** **AKELGATDCL** **NPQDFKKPIQ** **EVITEMNDG- GVDFAIECIG** **NIAVMNAALE** **CTTVGWGTCV**

**X.tropicalis 2**  **GSSCAVFGLG** **GVGLSAVMGC** **KAAGASRIIG** **IDINSDKFEK** **ALELGATECI** **NPKDYDKPIQ** **QVISEMTGG-** **GVDFSIECIG** **IIDVMKAALE** **CTTVGWGTCA**

**Human 4**  **GSTCVVFGLG** **GVGLSVIMGC** **KSAGASRIIG** **IDLNKDKFEK** **AMAVGATECI** **SPKDSTKPIS** **EVLSEMTGN-** **NVGYTFEVIG** **HLETMIDALA** **SCHMNYGTSV**

**Rat 4**  **GSTCAVFGLG** **GVGLSVVMGC** **KAAGASRIIG** **IDINKDKFQK** **ALDVGATECI** **NPRDFTKPIS** **EVLSDMTGN-** **TVQYTFEVIG** **RLETMVDALS** **SCHMNYGTSV**

**Mouse 4**  **GSTCVVFGLG** **GVGLSVIMGC** **KAAGASRIIG** **IDINKDKFQK** **ALAVGATECI** **SPKDSTKPIS** **EVLSDMTGN-** **TVQYTFEVIG** **RLETMVDALS** **SCHMNYGTSV**

**Human 1B1**  **GSTCAVFGLG** **GVGLSAVMGC** **KAAGAARIIA** **VDINKDKFAK** **AKELGATECI** **NPQDYKKPIQ** **EVLKEMTDG-** **GVDFSFEVIG** **RLDTMMASLL** **CCHEACGTSV**

**Rat 1**  **GSTCAVFGLG** **GVGLSVVIGC** **KTAGAAKIIA** **VDINKDKFAK** **AKELGATDCI** **NPQDYTKPIQ** **EVLQEMTDG-** **GVDFSFEVIG** **RLDTMTSALL** **SCHSACGVSV**

**Mouse 1**  **GSTCAVFGLG** **GVGLSVIIGC** **KAAGAARIIA** **VDINKDKFAK** **AKELGATECI** **NPQDYSKPIQ** **EVLQEMTDG-** **GVDFSFEVIG** **RLDTMTSALL** **SCHAACGVSV**

**Rabbit 1**  **GSTCAVFGLG** **GVGLSVIMGC** **KAAGASRIIA** **VDINKDKFPK** **AKEVGATECI** **NPQDYKKPIQ** **EVIQEISDG-** **GVDFSFEVIG** **RLDTVVAALL** **SCHGACGTSV**

**Ostrich 1**  **GSTCAVFGLG** **GVGLSVVMGC** **KAAGASRIIG** **VDINKDKFAK** **AKELGATDCV** **NPKDFTKPIH** **EVLMEMTGL-** **GVDYSFEVIG** **HTETMAAALA** **SCHFNYGVSV**

**Quail 1**  **GSTCAVFGLG** **GVGLSVVMGC** **KAAGASRIIA** **IDINKDKFAK** **AKELGATECV** **NPKDFKKPIH** **EVLTEMTGK-** **GVDYSFEVIG** **RIETMTEALA** **SCHYNYGVSV**

**Chicken 1**  **GSTCAVFGLG** **GVGLSVVMGC** **KAAGASRIIA** **VDINKDKFAK** **AKELGATECI** **NPKDFKKPIH** **EVLTEMTGQ-** **GVDYSFEVIG** **RIETMTAALA** **SCHNNYGVSV**

**Alligator 1**  **GSTCAVFGLG** **GVGLSTIMGC** **KAAGASRIIG** **IDINKDKFAK** **AKELGATECI** **NPLDCKKPIQ** **EVLSEMTGG-** **GVDYSFEVIG** **RIDTMTAALA** **CCQDNYGTSV**

**Turtle 1**  **GSTCAVFGLG** **GVGLSVVMGC** **KAAGASRIIG** **VDINKDKFAK** **AKELGATECI** **NPQDFTKPIE** **DVLMELTGGD** **GVDYSFEVIG** **RIDTMKAAMA** **SCHKNYGMSV**

**Cobra 1**  **GSICAVFGLG** **GVGLSVVMGC** **KAAGASRIIG** **VDINKDKFAK** **AKEMGATECI** **NPLDFKKPIN** **EVLFDLTGGE** **GVDYSFEVIG** **RTETMISAFT** **SCHQNLGTSV**

**Uromastyx 1A**  **GSTCAVFGLG** **GVGLSAVMGC** **KAAGASRIIG** **VDINKDKFPK** **AKEMGATECV** **NPLDYKKPIN** **EVLFDLTGGE** **GVDYSFEVIG** **RTDTMTAALA** **SCHMDYGTSI**

**Uromastyx 1B**  **GSTCAVFGLG** **GVGLSAVMGC** **KAAGASRIIG** **IDINKEKFPK** **AKELGATECV** **NPLDYKKPIN** **EVLFDMTDGE** **GVEYSFEVIG** **RTDTMTAALA** **SCHNNYGTSV**

**Anole 1A**  **GSTCAVFGLG** **GVGLSAVMGC** **KAAGASRIIG** **VDINKDKFPK** **AKELGATECV** **NPLDFKKPIN** **EVLFDLTGGE** **GVDYSFEVIG** **RIDTMTAALA** **SCHNNYGTSV**

**Anole 1B**  **GSTCAVFGLG** **GVGLSTVMGC** **KAAGASRIIG** **VDINKDKFPK** **AKQLGATECV** **NPLDFKKPIN** **EVLFDMTDGK** **GVDYSFEVIG** **RTDTMMAAMT** **SCNINYGTSV**

**Anole 1C**  **GSTCAVFGLG** **GVGLPVIIGC** **KTSGASRIIG** **IDINKAKFPI** **AKELGATECI** **SPLDFKKPIN** **EVLLDMTDGE** **GVDYSFEVIG** **HTDTMAAALA** **SCNLNYGVSV**

**Anole 1D**  **GSSCAVFGLG** **GVGLAVVMGC** **KAAGASRIIG** **INRSKEKFPK** **AKEVGATECV** **SPLDFKKPIQ** **EVLLDMTDGE** **GVDYSFEVVG** **STETMIAALA** **SCNMNYGTSV**

**Anole 1E**  **GSSCAVFGLG** **GVGLPVVMGC** **KAAGASRIIG** **INRSKDKFPI** **AKELGATECV** **SPLDFKKPIN** **EVLLEMTDGE** **GVDYSFEAIG** **RTDTMIAALA** **SCNMNYGTSV**

**Anole 1F**  **DSSCAVFGLG** **GVGLSVVMGC** **KAAGASRIIG** **IDINKDKFPM** **AKELGATECI** **SPLDFKKPIN** **EVVLEMTEGE** **GVDYSFEVIG** **RTDTMVAALA** **SCNMNYGVSV**

**Anole 1G**  **GSTCAVFGLG** **GVGLSAVMGC** **KAAGASRIIG** **INRSKERFPK** **AKELGATECV** **SPLDFEKPIN** **EVLFDMTDGD** **GVDYSFEATG** **CTDTMAAALA** **SCNRNHGISV**

**Anole 1H**  **GSTCAVFGLG** **GVGLSAVMGC** **KAAGASRIIG** **VDINKDKFPK** **AKEVGATECV** **NPLDFEKPIN** **EVLHDMTEGD** **GVDYSFEVVG** **HTDTMIAALT** **SCNMNYGTSV**

**Rana 1**  **GSTCAVFGLG** **GVGLSVIIGC** **KVAGASRIIG** **VDLNSDKFTT** **AKECGATECI** **NPKDYNIPIH** **EVLAKMTDD-** **GVDYAFEVIG** **NTTVMTSALS** **SSHFGCGKTV**

**X.tropicalis 1B**  **GSSCAVFGLG** **GVGLSVLIGC** **KVAGASKIIG** **VDTNSDKFAK** **AKELGATECI** **NPNDYNEPIH** **EVLAKISDG-** **GLDYTFECIG** **NTKVMESALK** **ATHFGCGTSV**

**X.laevis 1B**  **GSSCAVFGLG** **GVGLSVLIGC** **KVAGAAKIIG** **VDTNPDKFAK** **AKELGATECI** **NPNDYKEPIH** **EVLAKMSNG-** **GLDYTFECIG** **NTKVMESALL** **ATHFGCGTSV**

**X.tropicalis 1A**  **GCTCAVFGLG** **GVGLSVIIGC** **KVAGAAKIIG** **VDTNSDKFAK** **AKEVGATECI** **NPNDYKEPIH** **KVLEKMTDG-** **GLDYSFECIG** **NTRVMASALL** **STKFACGTSV**

**X.laevis 1A2**  **GSTCAVFGLG** **GVGLSVVIGC** **RVAGAAKIIG** **VDTNSDKFPK** **AKEIGATECV** **NPNDYKEPIH** **KVLEKMTEG-** **GLDYSFECVG** **NTSVMASALL** **STNYSYGTSV**

**X.tropicalis 1C**  **GSTCAVFGLG** **GVGLSGIIGC** **KAAGASRIIG** **VDTNSKKFTA** **AKNVGATECI** **NPNDYKEPVH** **EVLTKMTGL-** **GTDYSLEFVG** **DTNVMLSALL** **STNFASGTTV**

**X.laevis 1C**  **GSTCAVFGLG** **GVGLSGVIGC** **KAAGASKIIG** **VDTNSNKFTA** **AKKLGATECI** **NPNDIKEPVH** **EVLTKMTGL-** **GVDYSFEFVG** **DTNVMLSALL** **STNFASGTTV**

**Rana 8**  **GSTCAVFGLG** **GVGFSAIVGC** **KAAGASRIIG** **VGTHKDKFPK** **AIELGATECL** **NPKDYDKPIY** **EVICEKTNG-** **GVDYAVECAG** **RIETMMNALQ** **STYCGSGVTV**

**X.tropicalis 8B**  **GSTCAVFGLG** **GVGFSALIGC** **KISGAGRIIG** **VGSHKDKFPK** **AIELGATECL** **SPKDNDKPIQ** **EVIRDMTNG-** **GVDFAFECSG** **NIETLKTAFE** **STYIGSGVTV**

**X.laevis 8B**  **GSTCAVFGLG** **GVGFSAMIGC** **KIAGAGRIIG** **VGSHKDKFPK** **AIELGATECL** **SPKDYDKPIQ** **EVIREKTNG-** **GVDFAFECSG** **NIDTMKAAFE** **STYIGNGVTV**

**X.laevis 8A**  **GSTCAVFGLG** **GVGFSTIVGC** **KVAGAGRIIG** **VGSQKDKFEK** **AIELGATECL** **SPKDYDKPIQ** **EVIRDMTNG-** **GVDFAFECTG** **YIETMKTAFE** **STYIGNGVTV**

**X.tropicalis 8A**  **GSTCAVFGLG** **GVGFSALIGC** **KIAGAGRIIG** **VGSHKDKFPK** **AIELGATECL** **SPKDYDKPIQ** **EVIRDMTNG-** **GVDFAFECTG** **YIETMKTAFD** **STYLGNGVTV**

**X.tropicalis 9**  **GSACAVFGLG** **GIGMSVVMGC** **KVAGACRIIG** **VDINKGKFEI** **AKKLGCTECL** **DPNDYDKPIH** **EVIANMTDG-** **GVDYSFECVG** **NVDLMACVIQ** **SCHYSYGCAT**

**X.laevis 9**  **GSSCAVFGLG** **GIGMSAVMGC** **KVAGASRIIG** **VDINKGKFEI** **AKKLGCTECL** **DPNDYDKPIH** **EVIAEMTNG-** **GVDYSFECVG** **KVELMASVIQ** **ACHFSFGCAT**

**X.tropicalis 10B** **ESTCAIFGLG** **GIGLAVIMGC** **KIAGAARIIG** **VDINPDKFDI** **AKELGATECI** **NPNDYDKPVA** **EMILEQTGG-** **GVDYAFECVG** **HAETMLAALH** **SSHFAFGTTV**

**X.laevis 10B**  **GSTCVIFGLG** **GIGLAVIMGC** **KIAGAGRIIG** **VDVNPDKFDK** **AKELGATECI** **NPKDYDKPVA** **QVIVEQTGG-** **GVDYAFECVG** **HAETMLAALH** **SSHFAYGTTV**

**X.tropicalis 10A** **GSTCAIFGLG** **GIGLAVIMGC** **KIAGAARIIG** **VDINPDKFNI** **AKELGATECI** **NPKDYDKPVP** **QVILEQTGG-** **GVDYAFECVG** **HIETMLAALN** **SSHFAYGTTV**

**X.laevis 10A**  **GSTCVIFGLG** **GIGLAVIMGC** **KIAGAGRIIG** **VDVNPDKFDK** **AKELGATECI** **NPKDYDKPVA** **QVIVEQTGG-** **GVDYAFECVG** **HIETMLAALN** **SSHFAYGTTV**

**Human 5**  **GSTCAVFGLG** **GVGLSVVMGC** **KAAGAARIIG** **VDVNKEKFKK** **AQELGATECL** **NPQDLKKPIQ** **EVLFDMTDA-** **GIDFCFEAIG** **NLDVLAAALA** **SCNESYGVCV**

**Bovine 5 GSTCAVFGLG** **GVGLSVIMGC** **KAAGATRIIA** **VDINKDKFEK** **AKEVGATECI** **NPQDYEKPIQ** **EVLFDLTGD-** **GVDFSFEVIG** **NPETVAAALA** **SCRESHGVCV**

**Rabbit 5**  **GSTCAVFGLG** **GVGLSVIMGC** **KAAGAARIIG** **VDINKDKFKK** **AEEVGATECI** **SPQDFKKPIQ** **EVLFDMTGA-** **GVEFCFEVIG** **NPDTVAAAVA** **SCNESHGVCV**

**Rat 5**  **GSTCAVFGLG** **GVGLSVIIGC** **KAAGAARIIA** **VDINKDRFAK** **AKTVGATDCV** **DPRDFEKPIE** **EVLSDMIDG-** **GVDFCFEVTG** **NTEAVGAALG** **SCHKDHGVCV**

**Deer mouse 5**  **GSTCAVFGLG** **GVGLSVIMGC** **KAAGAARIIA** **VDTNKDKFAK** **AKTVGATECI** **DPQDFEKPIQ** **QVLFDMMND-** **GADFTFEVTG** **NPETVETALA** **SCHKDHGVCV**

**X.tropicalis 7**  **GSTCAVFGLG** **GIGLSAVIGC** **KSAGAAIIIA** **VDINSAKFDI** **AKVFGATECI** **NPLDYSKPIQ** **EVITEMTNG-** **GVHYSFECIG** **NTDTMKAALE** **CCHMGYGTSV**

**Turtle 7**  **GSTCAVFGLG** **GVGLSVVMGC** **KSAGAARIIG** **IDINKNKFVK** **AKELGATECI** **DPQDLKKPVQ** **EVLVEMTDH-** **GVDYSFEVIG** **RVDTMTAALE** **SCHMGSGTCV**

**Chicken 7**  **GSTCAVFGLG** **GVGLSVVMGC** **KAAGASRIIA** **IDINKDKFAK** **AKEMGATECI** **NPQDFKKPIQ** **QVLTEMTGH-** **GVDYSFEAIG** **TADTLIAALA** **SCNMNTGVCV**

**Zebra finch 7**  **GSTCAIFGLG** **GVGLSVVMGC** **KAAGAARIIA** **VDINKDKFAK** **ARELGATDCI** **NPRDFNKPTQ** **EVLTEMTGQ-** **GVNYSFEAIG** **HVDTMIAALA** **SCNASTGVCV**

**Pigeon 7**  **GSTCAVFGLG** **GVGLSVVMGC** **KVAGASRIIA** **IDINKDKFAK** **AKELGATDCI** **NPQDFNKPIQ** **EVVTEMTGH-** **EVDYSFEVIG** **RADTMIAALA** **SCNMNTGVFV**

**Anole 7A**  **GSSCAIFGLG** **GVGLSIIMGC** **KAAGASRIIG** **IDINKNKFAK** **AKELGATECL** **SPDDFKKPIN** **EVLVEITGL-** **GVDYTFEAIG** **LVETTVAALA** **SSHMAHGVCV**

**Anole 7B**  **GSTCAIFGLG** **GVGLSVVMGC** **KAAGASRIIG** **IDINKDKFLK** **AKELGATECL** **NPQDYKKPIQ** **ELLVEMTGH-** **GVDYAFEVVG** **CLDTLTAALA** **SCHLGCGVCV**

**Anole 7C**  **GSSCAIFGLG** **GVGLSVIMGC** **KAAGASRIIG** **VDINKDKFTK** **ARELGATECI** **SPEDFEKPIN** **EVLMEMTGL-** **GVDYAFEVIG** **RVDIEAAALA** **SCHLGHGVCV**

**Rat 6A**  **GSTCVVFGLG** **GVGSAIVMGC** **KASGASRIIG** **VDINEQKFPR** **ARALGVTDCL** **NPKKLEKPVQ** **EVVKEMTGV-** **GVDFAFEAIG** **QVDTMAAAWN** **SCNHSYGVCL**

**Mouse 6A**  **GSTCVVFGLG** **GVGSAIVMGC** **KASGASRIIG** **VDINEEKFPR** **ARALGVTDCL** **NPNKLEKPVQ** **EVVMEMTGV-** **GVDFAFEAIG** **LVDTMVAAWN** **SCNNSYGVCL**

**Rat 6B**  **GSTCVVFGLG** **GIGSAIVMAC** **KASGASRIIR** **VDTDEQKFPR** **ARALGVTDCL** **NPKKLEKPVQ** **KVVKEMTGV-** **GVDFAFEAIG** **LIETMVSALK** **SCNRSSGVCV**

**Mouse 6B**  **GSSCVVFGLG** **GIGSAIVMAC** **KASGACRIIG** **VDINEEKFPR** **ARALGVTDCL** **NPNKLKKPVQ** **EVVKEMTGV-** **GVDFAFEAIG** **LIETMVAALK** **SCNRSYGVCV**

**Bovine 6 GSTCVVFGLG** **GIGSAIVMGC** **KASGASRIIG** **VDINEEKFPR** **ARALGVTDCL** **NPSNLKKPVQ** **EAVKEMTGT-** **GVDFAFEAIG** **LAETMVAAWD** **SCHVSHGVCI**

**Dog 6**  **GSTCVVFGLG** **GIGSAIVMGC** **KASGASRIIG** **VDINEEKFPR** **ARALGVTDCL** **NPQKFKKPVQ** **QVVMEMTGV-** **GADFAFEAIG** **LSDTMLAAWD** **SCHRSYGVCL**

**Panda 6**  **GSTCVVYGLG** **GIGSAIVMGC** **KASGAARIIG** **VDINEEKFPR** **ARALGVTDCL** **NPRKLKKPVQ** **QVVMEMTGV-** **GADFAFEAIG** **LPDVMLAAWD** **SCHLSHGVCL**

**Horse 6**  **GSTCVVFGLG** **GIGSAVVMAC** **KASGASRIIG** **VDINEEKFPR** **ARALGVTDCL** **NPQKLKKPVQ** **QVVVEMTGF-** **GVDFAFEAVG** **LIDTMVASLE** **SCHLSYGVCV**

310 320 330 340 350 360 370 380

....|....| ....|....| ....|....| ....|....| ....|....| ....|....| ....|....| ....|....| ....|....

**Human 3**  **VVGVAAS-GE** **EIATRPFQLV** **TGRTWKGTAF** **GGWKSVESVP** **KLVSEYMSKK** **IKVDEFVTHN** **LSFDEINKAF** **ELMHSGKSIR** **TVVKI----**

**Rat 3**  **VVGVAAS-GE** **EISTRPFQLV** **TGRTWKGTAF** **GGWKSVESVP** **KLVSEYMSKK** **IKVDEFVTGN** **LSFDQINKAF** **DLMHSGNSIR** **TVLKL----**

**Mouse 3**  **VVGVAAS-GE** **EISTRPFQLV** **TGRTWKGTAF** **GGWKSVESVP** **KLVSEYMSKK** **IKVDEFVTGN** **LSFDQINQAF** **DLMHSGDSIR** **TVLKM----**

**Rabbit 3**  **VVGVAGA-GE** **EISTRPFQLV** **TGRTWKGTAF** **GGWKSVESVP** **KLVSEYMSKK** **INVDEFVTNT** **LSFDQINEAF** **ELMHSGKSIR** **TVVKI----**

**Chicken 3**  **IVGVAAA-GQ** **EISTRPFQLV** **TGRTWKGTAF** **GGWKSVDSVP** **KLVNDYMAKK** **IKVDEFVTHT** **LPFDKINEAF** **DLLHKGKSIR** **TVLKF----**

**Turtle 3**  **IVGVAAA-GQ** **EIATRPFQLV** **TGRTWKGTAF** **GGWKSVESVP** **KLVTEYMSKK** **IKVDEFVTHT** **LPFDRINEAF** **ELMHAGKSIR** **SVLKF----**

**Uromastyx 3**  **IVGVAAA-GQ** **EIATRPFQLV** **TGRTWKGTAF** **GGWKSVESVP** **KLVDEYMSKK** **MKVDEFVTHT** **LPFEQINEAF** **ELMHAGKSIR** **SVLKF----**

**X.tropicalis 3**  **IVGVAAS-GQ** **EIATRPFQLV** **TGRVWKGTAF** **GGWKSVDSVP** **KLVSEYMAKK** **IKVDEFVTHT** **LPFNSINEAF** **ELMHAGKSIR** **GVLNY----**

**X.laevis 3**  **IVGVAAS-GQ** **EIATRPFQLV** **TGRVWKGTAF** **GGWKSVDSVP** **KLVSEYMAKK** **IKVDEFVTHT** **LPFDSINEAF** **ELMHAGKSIR** **SVLNY----**

**Anole 3**  **IVGVAAA-GQ** **EIATRPFQLV** **TGRTWKGTAF** **GGWKSVESVP** **KLVAEYMSKK** **MKVDEFVTQN** **LPFEKINEAF** **ELMHAGKSIR** **TVLKF----**

**Marmoset 2**  **FVGVAPG-NK** **GLTLFPETLI** **IGRTINGTFF** **GGWKS-DSIP** **KLVTDYKNKK** **FDLDALVTHT** **LPFDKLSEAF** **DLMNQGKSIR** **TILIF----**

**Bovine 2 IIGVNVG-DN** **GLNVSAMELL** **MGRTLTGTSF** **GGWKGVTSVP** **KLAADYKNKK** **LDLDALVTHT** **LPFDKVNEAF** **DLMYQGKSIR** **TVLLF----**

**Human 2**  **FIGVAAG-SK** **GLTIFPEELI** **IGRTINGTFF** **GGWKSVDSIP** **KLVTDYKNKK** **FNLDALVTHT** **LPFDKISEAF** **DLMNQGKSVR** **TILIF----**

**Rat 2**  **VVGAKV---D** **EMNISTVDMI** **LGRSVKGTFF** **GGWKSVDSVP** **NLVTDYKNKK** **FDLDLLVTHA** **LPFDKINDAI** **DLMNQGKSIR** **TILTF----**

**Mouse 2**  **VVGAKV---D** **EMTIPTVDVI** **LGRSINGTFF** **GGWKSVDSVP** **NLVSDYKNKK** **FDLDLLVTHA** **LPFESINDAI** **DLMKEGKSIR** **TILTF----**

**Rabbit 2A**  **FVGVNLA-DK** **GLTISPIELI** **LGRTLKGTNF** **GGWD-AETVP** **KLVSDYKNGK** **FDLDALVTHT** **LPFDKINEAL** **NLLDQGKSIR** **TVLIF----**

**Rabbit 2B**  **FVGVNVN-DK** **GLTISPVELI** **LGRTLRGSSF** **GGWD-VDTVP** **KLVSDYKNGK** **FNLEALVTHT** **LPFEKINEAL** **DLLKQGKSIR** **TILIY----**

**Ostrich 2**  **IVGVAVG-EQ** **SIPFSPMQLI** **MGRKIKATFF** **GGWKSVKSVP** **KLVSDYMAKK** **FDLDALVSHT** **LPLDKINDAF** **DLMNAGKSNR** **TILVF----**

**Turtle 2**  **IVGVAVG-GQ TIAVSPMQLI** **MGKKINATFF** **GGWKSIDSVP** **KLVSDYMAKK** **FNLDALVTYT** **LPFEKINKAF** **DLMCEGKSTR** **TVLVF----**

**X.tropicalis 2**  **IVGVSLD-EH** **GLPVAPFHLL** **MGRTLKATFF** **GGWKSVDNVP** **KLVEDYLGNK** **FDLDSLVTFT** **LPFDKINEAF** **DLMRDGKSIR** **TVLVF----**

**Human 4**  **VVGVPPS-AK** **MLTYDPMLLF** **TGRTWKGCVF** **GGLKSRDDVP** **KLVTEFLAKK** **FDLDQLITHV** **LPFKKISEGF** **ELLNSGQSIR** **TVLTF----**

**Rat 4**  **VVGAPPS-AK** **MLSYDPMLLF** **TGRTWKGCVF** **GGWKSRDDVP** **KLVTEFLEKK** **FDLGQLITHT** **LPFHNISEGF** **ELLYSGQSIR** **TVLTF----**

**Mouse 4**  **VVGAPPS-AK** **MLTYDPMLLF** **TGRTWKGCVF** **GGWKSRDDVP** **KLVTEFLEKK** **FDLDQLITHT** **LPFNNINEGF** **ELLYSGKSIR** **TVLTF----**

**Human 1B1**  **IVGVPPA-SQ** **NLSINPMLLL** **TGRTWKGAVY** **GGFKSKEGIP** **KLVADFMAKK** **FSLDALITHV** **LPFEKINEGF** **DLLHSGKSIR** **TVLTF----**

**Rat 1**  **IVGVPPS-AQ** **SLSVNPMSLL** **LGRTWKGAIF** **GGFKSKDAVP** **KLVADFMAKK** **FPLEPLITHV** **LPFEKINEAF** **DLLRAGKSIR** **TVLTF----**

**Mouse 1**  **VVGVPPN-AQ** **NLSMNPMLLL** **LGRTWKGAIF** **GGFKSKDSVP** **KLVADFMAKK** **FPLDPLITHV** **LPFEKINEAF** **DLLRSGKSIR** **TVLTF----**

**Rabbit 1**  **IVGVPPD-SQ** **SLTVNPMLLL** **SGRTWKGAIF** **GGFKSKDSVP** **KLVADFMAKK** **FSLDPLITNV** **LPFEKINEGF** **DLLRSGKSIR** **TILTF----**

**Ostrich 1**  **IVGVPPA-AE** **KLSFDPMLLF** **SGRTWKGSVF** **GGWKSKDSVP** **KLVADYMEKK** **FVLDPLITHT** **LPFHKINEGF** **DLLRTGKSIR** **SVLLF----**

**Quail 1**  **IVGVPPA-AQ** **KISFDPMLIF** **SGRTWKGSVF** **GGWKSKDAVP** **KLVADYMKKK** **FVLDPLITHT** **LPFTKINEGF** **DLLRTGKSIR** **TVLVL----**

**Chicken 1**  **IVGVPPA-AQ** **KISFDPMLIF** **SGRTWKGSVF** **GGWKSKDAVP** **KLVADYMKKK** **FVLDPLITHT** **LPFTKINEGF** **DLLRTGKSIR** **SVLVL----**

**Alligator 1**  **IVGVPPA-SE** **KITFNPMMLF** **TGRTWKGSVF** **GGWKSKESVP** **KLVADYMEKK** **INLDGLITHT** **LPFDKINEGF** **ELLRTGKSIR** **SVLTF----**

**Turtle 1**  **IVGVPPS-AS** **QISFNPMLLF** **SGRTWKGSVF** **GGWKSKDSVP** **KLVADYLGKK** **INLDALITHT** **LPFDKINEGF** **ELLRTGKSIR** **TVLIF----**

**Cobra 1**  **VVGVPPN-AS** **MITYNPLMLF** **TGRTWKGCVF** **GGWKSKDSVP** **KLVSDFMQKK** **FVLDPLITHT** **LPFEKINEGF** **DLLRSGKSIR** **TVLIF----**

**Uromastyx 1A**  **IVGLPPS-AS** **EITFSPGLIF** **TGRTWKGSVF** **GGWKSKDSVP** **RLVSDFMQKK** **FSLDPLITHT** **MPFDKINEGF** **ELLRAGKSIR** **SVLLF----**

**Uromastyx 1B**  **IVGVPPS-AS** **QIAFDPLLLF** **TGRTWKGSVF** **GGWKSKDAVP** **RLVSDFMGKK** **FILDPLITHT** **MPFEKINEGF** **ELLRSGKSIR** **TVLTF----**

**Anole 1A**  **IVGVPPS-AA** **QISLDPMLLF** **TGRTWKGSVF** **GGWKSKDSVP** **KLVSDFMGKK** **FILDPLITHT** **MPFEKINEGF** **ELLRSGKSIR** **TVLTF----**

**Anole 1B**  **IVGVPPS-AS** **EITFSPGLIF** **SGRNWKGSLF** **GGWKSKDSVP** **RLVSDFMQKK** **FVLDPLITHT** **MPFDKINEGF** **ELLRSGKSIR** **TVLVF----**

**Anole 1C**  **MVGVPPS-AA** **EMTLSPRLIF** **TGRTWKGSVF** **GGLKSKDDIP** **KLVLAGMEKK** **FNLDPLVTHV** **LPFEKINEGF** **ELLHKGQSIR** **TVLKM----**

**Anole 1D**  **VVGAPPS-AS** **EMTLSPTLIF** **TGRTWKGSVL** **GGLKSKDDVP** **VLVSELMAKK** **FNLDPLITHV** **LPFDKINEGF** **ELLRNGKCIR** **TVLKM----**

**Anole 1E**  **MVGLPPS-EA** **EMTLSPTLIF** **TGRTWKGSLF** **GDMRGKEDVP** **KLVSELMAKK** **FNLDPIITHV** **LPFDKINEGF** **ELLRNGKCIR** **TVLKM----**

**Anole 1F**  **IVGAAPS-AS** **EVTLSPTLIF** **TGRTWKGSVF** **GGLKSKDAVP** **KLVLELMSKK** **FILDPLITHV** **LPFDKINEGF** **ELLHKGESIR** **TVLKM----**

**Anole 1G**  **MLGFSPA-LS** **KISFSPALIL** **TGRTWKGCLF** **GGWKSKDAVP** **KLVLDFMQKK** **FTLDPLITHT** **MPFDKINEGF** **ELLREGKSIR** **SVLVF----**

**Anole 1H**  **MVGAPAT-GS** **EITLPPGLII** **TGRVWKGTVF** **GGWKSRDSIP** **RLVSDFMQKK** **FTLDPLITHT** **MPFDKINEGF** **ELLQAGKSIR** **SILIF----**

**Rana 1**  **IVGLAPS-SA** **VMSFDPLLIL** **TGRILTGAVF** **GGWKSKDDVP** **KLVRDYLNKK** **FDFDPLITHY** **MPFEKINEGF** **ELLRNGKSIR** **TILTF----**

**X.tropicalis 1B**  **IVGLAPA-SA** **RVSVDPMEML** **TGRTLKGALF** **GGWKSRDEVP** **QLVADFLAKK** **FELDGLITHR** **STLDKINEGF** **DLLRKGDSIR** **TILQISQ--**

**X.laevis 1B**  **IVGLAPA-SA** **RVLVDPMEML** **TGRTLKGALF** **GGWKSRDDVP** **KLVADFMAKK** **FELDGLITHR** **STLDKINEGF** **DLLRKGDSIR** **TILQISQ--**

**X.tropicalis 1A**  **IVGVAPS-TA** **ELNLDPMVIL** **TGRTLKGSLF** **GGWKSKDCVP** **KLVADFMENK** **FELDGLISHK** **LPLQKINEGF** **DLLHKGTSLR** **TVLYF----**

**X.laevis 1A2**  **IVGVAPL-AA** **ELNLNPMEIL** **TGRTLKGTLF** **GGWKGRDCVP** **KLVADFMAKK** **FELDGLITHR** **LPLQNINEGF** **DLLHKGESLR** **TVLYFC---**

**X.tropicalis 1C**  **IVGLAHY-TA** **KMNFNPMVLL** **TGRTLKGGLL** **GGCKSKD-IP** **KLVSDLMAKK** **FDLDGLITHK** **MPLAKINEAY** **DVLTKGESLR** **TILMMSSCE**

**X.laevis 1C**  **IVGLAHA-TA** **KMSFDPMVLL** **TGRTLKGSLL** **GGFKSKD-IP** **KLVCDLMAKK** **FDLDGLISHK** **MPLAKINEAY** **DLLNKGESLR** **TILIM----**

**Rana 8**  **VLGLASP-NE** **RLPLDPLLLL** **TGRSLKGSVF** **GGFKG-EEVS** **KLVDDYMKKK** **INVNFLVSTK** **LTLDQINKAF** **ELLSSGQGVR** **SIMIY----**

**X.tropicalis 8B**  **LLGVAGP-ND** **KLCFHPGEVM** **MGRTIKGLPY** **GGFKGRDDIP** **KLVGDYMANK** **FNLNFMVSER** **MPLEKINEAF** **ELMASGKGLR** **NLIIF----**

**X.laevis 8B**  **VLGVAGP-ND** **KLCFHPGEVM** **MGRTIKGLPY** **GGFKSRDDIP** **KLVCDYMAKK** **FNLEFMVSQR** **LPVEKINEAF** **ELMASGKGLR** **NLIIF----**

**X.laevis 8A**  **VLGVAGP-DD** **RLSFHPGELL** **LGRTMKGSAF** **GGFKGRDEVP** **MLVSDYMAKK** **YDIKFLVSEK** **MPLEKINEAF** **ELMKMGKGVR** **NIIIY----**

**X.tropicalis 8A**  **VLGVAGP-DD** **RLSFHPGELL** **FGRTMKGSAF** **GGFKSRDEVP** **MLVSDYMEKK** **FNLDFMVSER** **IPLEKINEAF** **ELMQSGKGVR** **NIIIF----**

**X.tropicalis 9**  **IIGVPPS-TA** **RLCLDLIWLL** **TGRTLKGAFL** **GDYKAKEAFP** **GLVKDAMNKK** **FDIGALVTHR** **VKFDKIMDGF** **ELMRHGKCVR** **AVLDM----**

**X.laevis 9**  **IIGVPPS-TA** **RLSLDLMWLL** **TGRTVKGAFL** **GDWKAKEAFP** **RLVQDAMKKK** **FPLKALVTHR** **VKFDKIMDGF** **ELMRQGKCVR** **AALDM----**

**X.tropicalis 10B** **IIGASA---S** **TLSFDPMILL** **SGRTLKSSSF** **GGWKSRLEVP** **KLVSDYLAKK** **FDLEKLVTHR** **LPFQKISEGF** **DLLHSGKCIR** **TILKF----**

**X.laevis 10B**  **IIGVSA---S** **TLSFDPMILL** **SGRTLKSSML** **GGWKSKDSVP** **KLVSDYLAKK** **FDLEKLVTHR** **LPFQKIDEGF** **DLLLSGKCIR** **TILTFKAVD**

**X.tropicalis 10A** **IVGVSAP-DS** **TISFDPMILL** **TGRTIKGSVF** **GGWKSKNSVP** **QLVSDSLAKK** **FDLEKLVTHR** **LPLNKINEGF** **DLLRSGKSIR** **TILLV----**

**X.laevis 10A**  **IVGVSAK-EA** **TITFDPMILL** **TGRTLKGSVF** **GGWKSKSSVP** **QLVLDSMAKK** **FDLEKLVTHR** **LPLDKINEGF** **DLLHSGKSIR** **TILLV----**

**Human 5**  **VVGVLPA-SV** **QLKISGQLFF** **SGRSLKGSVF** **GGWKSRQHIP** **KLVADYMAEK** **LNLDPLITHT** **LNLDKINEAV** **ELMKTGKCIR** **CILLL----**

**Bovine 5 IIGLIIG--V** **QLNISGHLFF** **TGRSLKGSVY** **GGWKGRDGAS** **KLVSDYMAKK** **INLDALITHS** **LNLDKINEAV** **ELMKTGKCVR** **CVLLL----**

**Rabbit 5**  **IVGLMSA-TD** **QVRIHGNLFF** **SGRTLKGSVF** **GGWKSREHVP** **KLVSDYIEKK** **FNLEPLITHT** **LNLDKINEAS** **ELMKTGKCIR** **CVLLP----**

**Rat 5**  **TVGALASFTS** **TLSIRSHLFF** **SGRILKGSIL** **GGWKTKEEIP** **KLVSDYMAKK** **FNIDPLITHT** **LTLSEANEAV** **QLMKSGQCIR** **CVLLL----**

**Deer mouse 5**  **IVGSLAS-WI** **QLNINSHLFF** **SGRTLKGSVL** **GGWKTKEEIP** **KLVSDYTAKK** **FNLDPLITHT** **LTLDKVNEAI** **QLMKNGQCIR** **CVLLP----**

**X.tropicalis 7**  **IIGEAPS-AA** **QISFDPILLF** **TGRTWKGSIF** **GGWKSKESVP** **RLVDEFMANK** **FNLDGLVSHT** **LPFDQINEGF** **ELLRSGNSIR** **TILMF----**

**Turtle 7**  **MVGTPPA-GS** **RISFDPMLLF** **TGRTWKGSFI** **GGWKTKDSIP** **KLVSGYMEKK** **FNPDVLITHT** **LPFEKISEGF** **ELLCSGKSIR** **SILLF----**

**Chicken 7**  **MVGVLPA-GS** **TVPIDPFLLL** **SGRTCKGTLA** **GGWKMRDSIP** **KLVASYLEKK** **FNSDLLITHT** **LPFAKINEGF** **ELLRAGKSIR** **TVMLF----**

**Zebra finch 7**  **MVGVL---NS** **EISIDPVLLL** **TGRTWKGTLL** **GGWKTRECIP** **KLVSSYLEGK** **FNSDLLITHT** **LPFAKVNEGF** **ELLRAGKSIR** **TVLLF----**

**Pigeon 7**  **MVGVAPS-DA** **VISVDPLLLL** **TGRTHKGTLV** **GGSKGRNFIP** **RLVSSYLEKK** **FNSDLLITHT** **LPFAKVNEGF** **ALLHAGKSIR** **TVLLF----**

**Anole 7A**  **VVGEPPA-GS** **HLSVDPVLLI** **TGRKLLGCCM** **GGFRLKDAIP** **KLVSEYMEKK** **FNLDALRSHV** **LPFEKIGEGF** **ELLCSGKSIR** **SVLLY----**

**Anole 7B**  **MVGVPPL-GS** **RLTFDPMLLF** **TGRTLKGCFI** **GGWKMKDSIP** **QLVSSYMEKK** **FNSDALISHT** **LPFSQISEGF** **NLLHSGKSIR** **IVLVF----**

**Anole 7C**  **LVGAPPA-GS** **QLCISPLQLL** **TGKKLIGSLI** **GGWKLKDALP** **KLVSDYMANK** **FNTDALITHV** **LPFEKIGEGF** **ELLNSGKSIR** **CVLLF----**

**Rat 6A**  **IVGLAPS-DT** **HLSLEASKIL** **SGKTLKGVCL** **GDYKTRDCIP** **QIVTDYLQNK** **INIDPLVTHQ** **LPFSQLHKAL** **ELYHSGKTIR** **CVLLF----**

**Mouse 6A**  **IAGLAPS-DA** **QLSLEAPKIL** **SGKTLKGVCL** **GDYKTRDCIP** **QIVTDYLQNK** **INIDPLVTYQ** **LPFNQLHEAL** **ELFHSGKAIR** **CVLLF----**

**Rat 6B**  **IMGVAPT-GS** **QLSFDPVLLL** **PGRTLKSSVL** **GGYKTRDDIP** **KLVTDYVQXK FNIEPLITHR LPFPEINEGF KLLREGKCIR CVLSMRP--**

**Mouse 6B**  **IMGVAPT-GS** **QLSFDPLVLL** **PGRTLKSSVL** **GGYKTRDDIP** **KLVTDYVQKK** **INIDPLITHR** **LPFPKINEGF** **RLLQEGKCIR** **CVLSMRP--**

**Bovine 6**  **ITGVSPP-NS** **KFSLSAQTVS** **TGRTLKGVCL** **GDYKTKDCFP** **QLVTAYLQNK** **INIDPLITHQ** **LPFDQLHKAF** **KLYHAGKTIR** **CVLLF----**

**Dog 6**  **IVGVAPL-NS** **KLSLDAPMIV** **SGRTMKGVCL** **GDYKTRDCIH** **HLVTDYLQNK** **INIDPLVTHQ** **LPFDQLHKAF** **ELYHAGKTIR** **CILLF----**

**Panda 6**  **IVGVAPL-NS** **KLSLDASVIF** **SGRTLKGVCL** **GDYKTRDCIP** **RLVTDYLQNK** **INIDPLVTHQ** **LPFDQLHKAF** **ELYRAGKTIH** **CLLLF----**

**Horse 6**  **IIGAAPS-ES** **QLSFDPMLIL** **AGRTLKGGIL** **GKYKTRDSIP** **KLVTDYLQKK** **INIDPLITHK** **LPFERINEAF** **ELLQGGKCIR** **CVLSF----**
